# *Tetrahymena thermophila* glutathione-S-transferase superfamily: an eco-paralogs gene network differentially responding to various environmental abiotic stressors and an update on this gene family in ciliates

**DOI:** 10.3389/fgene.2025.1538168

**Published:** 2025-03-07

**Authors:** Ruth Ortega, Ana Martin-González, Juan-Carlos Gutiérrez

**Affiliations:** Department of Genetics, Physiology and Microbiology, Faculty of Biology, Complutense University of Madrid, Madrid, Spain

**Keywords:** glutathione S-transferases, ciliates, *in silico* analysis, qRT-PCR, metal(loid)s, *Tetrahymena thermophila*

## Abstract

Glutathione S-transferases constitute a superfamily of enzymes involved mainly, but not exclusively, in the detoxification of xenobiotic compounds that are considered environmental pollutants. In this work, an updated analysis of putative cytosolic glutathione S-transferases (cGST) from ciliate protozoa is performed although this analysis is mainly focused on *Tetrahymena thermophila*. Among ciliates, the genus *Tetrahymena* has the highest number (58 on average) of cGST genes. As in mammals, the Mu class of cGST is present in all analyzed ciliates and is the majority class in *Tetrahymena* species. After an analysis of the occurrence of GST domains in *T. thermophila*, out of the 54 GSTs previously considered to be Mu class, six of them have been discarded as they do not have recognizable GST domains. In addition, there is one GST species-specific and another GST-EF1G (elongation factor 1 gamma). A structural analysis of *T. thermophila* GSTs has shown a wide variety of β-sheets/α-helix patterns, one of the most abundant being the canonical thioredoxin-folding pattern. Within the categories of bZIP and C4 zinc finger transcription factors, potential binding sites for c-Jun and c-Fos are abundant (32% as average), along with GATA-1 (71% average) in the *T. thermophila* GST gene promoters. The alignment of all MAPEG (Membrane Associated Proteins involved in Eicosanoid and Glutathione metabolism) GST protein sequences from *Tetrahymena* species shows that this family is divided into two well-defined clans. The phylogenetic analysis of *T. thermophila* GSTs has shown that a cluster of 19 Mu-class GST genes are phylogenetic predecessors of members from the omega, theta and zeta classes. This means that the current GST phylogenetic model needs to be modified. Sixteen *T. thermophila* GST genes, together with two clusters including three genes each with very high identity, have been selected for qRT-PCR analysis under stress from eleven different environmental stressors. This analysis has revealed that there are GST genes that respond selectively and/or differentially to each stressor, independently of the GST class to which it belongs. Most of them respond to the two more toxic metal(loid)s used (Cd or As).

## 1 Introduction

Glutathione transferases, also known as glutathione-S-transferases (GSTs, E.C.2.5.1.18), are a family of ubiquitous enzymes present from prokaryotes to uni- or multicellular eukaryotes (including humans) (Allocati et al., 2006; Hao et al., 2021; Park et al., 2020; Strange et al., 2001). This enzyme superfamily contributes mainly to the detoxification of drugs, pesticides, and other xenobiotic compounds considered as environmental pollutants (Hayes et al., 2005; Oakley, 2005), and reducing oxidative stress caused by reactive oxygen species (ROS) (Raza, 2011). The eukaryotic xenobiotic detoxification process consists of three phases: phase I (enzymes that oxidize, reduce or add hydroxyl, carboxyl or amino radicals to the xenobiotic), phase II (enzymes that conjugate molecules such as amino acids, sugars or glutathione to the xenobiotic), in which GSTs are located, and phase III (excretion of the transformed xenobiotic out of the cell) (Ames et al., 1990; Liska, 1998). In phase-II, GSTs covalently conjugate a reduced glutathione (GSH) molecule to the electrophilic region of the hydrophobic xenobiotic, converting it into a more hydrosoluble molecule and facilitating its excretion out of the cell (Armstrong, 1991).

In addition to their important role in xenobiotic detoxification and antioxidant defense, GSTs have other cellular vital functions, such as involvement in S-glutathionylation reactions of diverse proteins (or post-translational protein modifications), cell signaling, or resistance to chemotherapy drugs used in the treatment of tumors (Lv et al., 2023; Mazari et al., 2023; Singh and Reindl, 2021). They are also involved in normal processes of cell development and differentiation (Laborde, 2010; Rowe et al., 1998).

By their origin or cellular location, these enzymes can be divided into four main groups: cytosolic (cGST), mitochondrial (mGST), microsomal or MAPEG (Membrane Associated Proteins involved in Eicosanoid and Glutathione metabolism) and GSTsFosA (bacterial fosfomycin resistance proteins) (Pearson, 2005). Cytosolic GSTs are the most numerous and each species possesses dozens of genes potentially encoding these enzymes. For example,: in mammals 15–23 GST genes have been reported, 40–61 genes in plants (although it can reach more than 300 genes in wheat species), a range of 30–35 in insects and 1–15 genes in bacteria (Hao et al., 2021; Hayes et al., 2005; McGonigle et al., 2000; Tu and Akgul, 2005; Vuilleumier and Pagni, 2002). A given species can have multiple GST isoforms, and in addition, they present intraspecific polymorphisms (Hayes et al., 2005). All these isoforms constitute the GSTome (Mannervik, 2012), within which at least 15 GST classes (named with letters of the Greek alphabet) are distinguished depending on their structural and amino acid sequence similarities (Frova, 2006). Sigma, Alpha, Mu and Pi classes are animal-specific, Delta and Epsilon are insect-specific, Phi, Tau and Lambda are plant-specific, and so on (Edwards and Dixon, 2005; Frova, 2006; Tu and Akgul, 2005; Udomsinprasert et al., 2005). Some organisms have proteins that appear to exhibit GST activity, such as EF1Bγ, Ure2p, MAK16 and others that cannot be included in any previously established (unclassified) class (McGoldrick et al., 2005).

*Tetrahymena thermophila* is a widely used and well-known model eukaryotic microorganism both in molecular biology studies (organism used by Nobel Prize winning researchers) and in studies on the effect of a wide range of environmental toxicants (Ruehle et al., 2016). This makes this microbial model a very useful tool to study genes involved in the cellular response to abiotic stressors.

Our current knowledge about the superfamily of GSTs from ciliate protozoa is considerably scarce. In 1988, a protein (33–35 KDa) with GST activity was isolated and purified from *T. thermophila* (Overbaugh et al., 1988). In *Blepharisma japonicum* (Takada et al., 2004) a cDNA encoding a GST, whose expression is induced by light stimulation, was characterized and considered as a new class of GSTs. Ortega et al. (2007) reported at the 3rd Cell Stress Society International Congress and 2nd Word Conference of Stress (Budapest, Hungary), part of the present study (now expanded and updated). Finally, in 2019, a paper on the *T. thermophila* GST family was published (Dede and Arslanyolu, 2019). In that publication an *in silico* analysis of genes identified as GSTs from *T. thermophila* genome website (www.ciliate.org) is reported, together with an analysis of microarray expression data during growth, starvation and conjugation of this ciliate, previously obtained from other authors (Miao et al., 2009; Xiong et al., 2013) and available in the *Tetrahymena* Functional Genomics Database (TetraFGD; http://tfgd.ihb.ac.cn/, currently replaced by *Tetrahymena* Gene Network Explorer (TGNE microarray https://tet.ciliate.org/common/gne/tet/TGNE_microarray_beta.html).

In the present work, we carried out an update of the *in silico* analyses previously performed (Dede and Arslanyolu, 2019; Ortega et al., 2007) on the *T. thermophila* GST superfamily, together with a comparative analysis with other *Tetrahymena* species whose macronuclear genomes are already sequenced. The *in silico* analysis involves: 1- An update on the number and classes of GSTs from *T. thermophila* (TthGST), 5 years since the last analysis (2019) and after several updates of the *Tetrahymena* genome website. A comparative analysis of GST genes from other *Tetrahymena* and ciliate species with sequenced genomes. 2- A comparative analysis of the main gene and protein structural features from cytosolic and MAPEG GSTs among ten species of the genus *Tetrahymena* and nine other selected ciliates. 3- A structural domain characterization of the different classes of *T. thermophila* GSTs. 4- Analysis of the promoter regions from the *T. thermophila* cGST genes, and 5- A phylogenetic analysis of cytosolic and MAPEG GSTs from *T. thermophila* and other *Tetrahymena* species. In addition, an analysis of the expression (by qRT-PCR) of 16 GST genes selected from *T. thermophila*, under eleven different abiotic stressors (metal(loid)s, drugs, pH, and starvation), 2 or 24 h of exposure, was carried out.

## 2 Materials and methods

### 2.1 Ciliate strain, culture conditions and stress treatments

*Tetrahymena thermophila* strain SB1969, kindly supplied by Dr. E. Orias (University of California, United States), was cultured in PP210 medium (2% proteose peptone (Pronadisa) supplemented with 10 μM FeCl_3_ and 250 μg/ml of both streptomycin sulfate and penicillin G (Sigma) for 24 h at 30 ± 1°C.

Log-phase 50 ml *T. thermophila* cultures (∼2 × 10^5^ cells/mL) were exposed to different stressful conditions. Metals/metalloids, such as 27 μM Cd(II) (CdCl_2_), 80 μM Cu(II) (CuSO_4_·5H_2_O), 604 μM Pb(II) (PbNO_3_)_2_, 30 μM As(V) (Na_2_HAsO_4_ · 7 H_2_O) or 870 μM Zn(II) (ZnSO_4_·7H_2_O) in PP210 medium for 2 or 24 h at 30°C. These metal concentrations correspond to approximately half the LC_50_ values calculated for *T. thermophila* strain SB1969 as previously reported (Díaz et al., 2007) and resulted in negligible cell mortality. The following organic compounds were used as oxidative stress inducers: the herbicide Paraquat (PQ) (1,1′-dime til-4,4′-bipyridyl dichloride) at 7,700 μM in PP210 medium (24 h exposure) (Díaz et al., 2016). Menadione (MD) (2-methyl-1,4-naphthoquinone), a 5 M solution in chloroform was prepared from which a 2,000 μM solution in PP210 medium was the final used concentration (2 h exposure) (Cubas-Gaona et al., 2020). CDNB (1-chloro-2,4-dinitrobenzene), a 10 mM ethanol solution was prepared and from this a 1/5 dilution was made until a 200 μM concentration in PP210 was obtained (2 h treatment). It is a substrate and inducer of GSTs (Armstrong, 1997), which causes superoxide anions and oxidative stress (Nordberg and Arner, 2001). All these compounds were purchased from Sigma-Aldrich. Other abiotic stressors were PP210 medium at basic or acidic pH (pH 9 or pH 5, 24 h exposures). Starvation stress was induced by maintaining the culture in 10 mM Tris-HCl buffer (pH 6.8) for 24 h.

### 2.2 RNA isolation and quantitative RT-PCR

Total RNA was isolated from control and treated *T. thermophila* cultures (∼1–3 × 10^5^ cells/mL) using the commercial RNeasy Mini Kit (Qiagen). RNA samples were treated with DNase I (Roche) at 37°C for 30 min. Subsequently 3 μg of each RNA sample was retrotranscribed to cDNA with the 1st Strand cDNA Synthesis Kit for RT-PCR (AMV) (Roche) by following the manufacturer’s directions. cDNA samples were amplified in duplicate in 96 microtiter plates (Applied Biosystems). Quantitative RT-PCR was carried out in 20 μL reaction mixtures containing 10 μL of SYBR Green PCR master mix 1x (Takara), 5 μL of each primer 0.2 μM (primer sequences, designed using the Oligo Xpress™ software, are showed in Supplementary Table S1 and 5 μL of a 1/10 cDNA dilution from each sample. α-tubulin gene (*TthATUB*) was used as an endogenous control or housekeeping gene. An exception was the *TthGSTM32* gene, which was amplified using a TaqMan probe. Reaction mixtures were made using FastStart TaqMan^®^ ProbeMaster (Roche), using 0.2 μL of the probe plus the corresponding primers (Supplementary Table S1). Probe number 41 from the Roche human probe library (Universal ProbeLibrary Probes Probe#41) was used for the *TthGSTM32* gene, and probe number 8 (Universal ProbeLibrary Probes Probe#8) was used for the α-tubulin gene.

Samples were amplified in an ABI PRISM^®^ 7900 HT Time PCR System thermal cycler using the following thermal cycling protocol: 10 min at 95°C, followed by 40 cycles of 15 s at 95°C, 30 s at 50°C and 20 s at 72°C; and a final step of 1 min at 95°C and 1 min at 50°C. The specificity of each primer pair was confirmed by melting curve analysis. Relative gene expression was quantified according to the delta-delta Ct method (Livak and Schmittgen, 2001). Quantification was done relative to the reference gene (α-tubulin) respective to each stress treatment (treated sample or control) by subtracting the cycle threshold (Ct) of the reference gene from the Ct of the corresponding gene. All non-template controls (NTC) and RT minus control were negative. Amplification efficiency (E) was measured by using 10-fold serial dilution of a positive control PCR template. Efficiency parameters were met for each gene (Supplementary Table S2).

### 2.3 Statistical and *in silico* analysis

Gene expression differences were tested for statistical significance by one-way ANOVA followed by Dunnett’s multiple comparisons test performed with GrapPad Prism 10.3.1.509. P-value was fixed at ≤0.05 for statistically significant values.

The GST sequences from the different ciliates with sequenced genomes were obtained from http://www.ciliate.org (TGD website). Supplementary Table S3 lists the names assigned to the putative 71 cGST genes registered for *T. thermophila* and their corresponding gene identifier (according to the TGD website).

For the detection of different conserved domains in the GSTs sequences, we used the web PROSITE (https://prosite.expasy.org/). The PROMO website was used for the analysis of the GST gene promoter regions (https://alggen.lsi.upc.es/cgi-bin/promo_v3/promo/promoinit.cgi?dirDB=TF_8.3). Sequence alignments and their percent identities were obtained from the BioEdit Sequence Alignment Editor program (Hall, 1999). Phylogenetic analysis was carried out using the web server NGPhylogeny.fr (https://ngphylogeny.fr/), and using the programs MAFFT for multiple alignment, BMGE for alignment curation, PhyML (software based on the maximum-likelihood) for tree inference, and Newick display (to display a phylogenetic tree as SVG) (Lemoine et al., 2019). The PSIPRED web server (http://bioinf.cs.ucl.ac.uk/psipred/) was used to analyze secondary structure prediction, including regions of disorder and transmembrane helix packing; contact analysis; fold recognition; structure modelling; prediction of domains and function. Likewise, the AlphaFold Protein Structure Database (https://alphafold.ebi.ac.uk/) was used to predict the 3D structure of GST proteins.

## 3 Results

### 3.1 Comparative analysis of ciliate cGST basic parameters

Table 1 shows the number of putative cytosolic GSTs assigned to the different classes reported from sequenced ciliate genomes (www.ciliate.org) vs. other selected organisms. Among ciliates from the class Oligohymenophorea, ten *Tetrahymena* species (Order Tetrahymenida), one of the genus *Ichthyophthirius* (Order Ophryoglenida) and one of the genus *Paramecium* (Order Peniculida) are analyzed. From the class Spirotrichea we have analyzed the genera *Stylonychia* and *Oxytricha* (Order Sporadotrichida) one species of each, two species of the genus *Pseudokeronopsis* (Order Urostylida), and one species of the genus *Euplotes* (Order Euplotida). Finally, from the class Heterotrichea, the genera *Stentor* and *Blepharisma* (Order Heterotrichida), one species of each, have been selected for this analysis. Therefore, we show nineteen ciliate species from very different taxa (Table 1). In addition, four species of flagellate parasitic protozoa and one amoeba species have been added. Among the multicellular organisms, two mammals (including humans), two plants and one insect, all of them eukaryotic model organisms, are also analyzed. Two model prokaryotic microorganisms (bacteria) are also included (Table 1).

**TABLE 1:** Number and classes of putative cytosolic GSTs obtained from ciliate genomes present on the websites vs. other selected organisms.

GST class	1	2	3	4	5	6	7	8	9	10	11	12	13	14	15
Theta	5	3	5	5	4	5	5	5	3	7		9	4	7	17
Omega	8	10	11	5	8	8	7	11	8	15				8	4
Zeta	2	1	1	1	1	1	1	1	1	1		5		2	
Alpha												1			
Mu	54	36	35	39	21	42	38	50	30	65	2	7	2	1	8
Sigma													8	7	14
Tau														1	
Pi															
Phi															
Lambda															
Delta-Epsilon														7	3
Beta															
Unclassified	2	2	2	2	2	2	3	1	2	2		2		2	
Total	71^a^	52	54	53	36	58	54	68	44	90	2	24	14	35	46

GST class	16	17	18	19	20	21	22	23	24	25	26	27	28	29	30	31
Theta	12	19	18	4						2	3	4	3	2		
Omega	3		5	2						2	2	4				
Zeta			2							1	1	2		3		
Alpha										6	6					
Mu	2	2	2	1					1	5	6					
Sigma	12	9	18	7	1	1			12	1	1	1				
Tau													28	39		
Pi										1	2					
Phi													13	16		
Lambda													2	1		
Delta-Epsilon	2		2	2								20				
Beta															5	1
Unclassified						6	1	1	6							
Total	31	30	47	16	1	7	1	1	19	18	21	31	46	61	5	1

(1) *Tetrahymena thermophila,* (2) *T. borealis*, (3) *T. canadensis*, (4) *T. elliotti*, (5) *T. empidokyrea*, (6) *T. malaccensis*, (7) *T. paravorax*, (8) *T. pyriformis*, (9) *T. shanghaiensis*, (10) *T. vorax*, (11) *Ichthyophthirius multifiliis*, (12) *Paramecium tetraurelia*, (13) *Blepharisma stoltei* (14) *Euplotes vanus*, (15) *Pseudokeronopsis carnea*, (16) *Pseudokeronopsis flava*, (17) *Stentor coeruleus*, (18) *Oxytricha trifallax*, (19) *Stylonychia lemnae* (20) *Giardia lamblia*, (21) *Plasmodium falciparum*, (22) *Trypanosoma cruzi*, (23) *T. brucei*, (24) *Acanthamoeba castellanii*, (25) *Homo sapiens*, (26) *Mus musculus*, (27) *Drosophila melanogaster* (28) *Arabidopsis thaliana*, (29) *Oriza sativa*, (30) *Escherichia coli*, (31) *Bacillus subtilis*.

^a^ : This number of putative GSTs will be corrected after the analysis carried out in this work. Numbers in blue boxes: GST class with the highest gene number. From 1 to 19: ciliate protozoa, from 20 to 23: parasitic flagellate protozoa, 24: amoebae, 25 and 26: mammals, 27: insects, 28 and 29: plants, 30 and 31: bacteria. Numbers in red: parasitic protozoa.

Among ciliates, the genus *Tetrahymena* has the highest number (58 on average) of putative cGST genes, according to information extracted from their sequenced genomes. *T. vorax* is the species with the highest number (90) of these genes (Table 1). The rest of the studied ciliates present a number ranging from 14 to 71 genes (in *T. thermophila* the definitive number of putative GSTs will be corrected after the analysis carried out in this work). Interestingly, parasitic protozoa, with an average of two genes (Table 1), show the lowest number of putative cGST genes, as it occurs in some bacteria.

The Mu class is the predominant in the *Tetrahymena* species (41 on average), and this class is present in all examined ciliates. In other ciliates, Theta or Sigma classes are predominant. Delta-Epsilon classes only occur in ciliates from the class Spirotrichea. Unclassified cGSTs are detected in protozoa; both parasitic and free-living (Table 1). Supplementary Figure S1 shows the percentage of selected ciliate species with a given cGST class. The cGST classes present in ciliates are ranked as follows: Mu > Theta > Omega > Zeta > Unclassified (UC) > Sigma > Delta-Epsilon (D-E) > Tau = Alpha (Supplementary Figure S1).

To characterize and differentiate the different classes of GSTs present in the ciliates, Supplementary Table S4 lists some parameters of the putative cGST genes and proteins from the 19 selected ciliate species. Among *Tetrahymena* species, the average size of GSTM (Mu class) proteins is in the range 211–248 amino acids (aa). GSTO (Omega class) has a size range of 231–296 aa, that of GSTT (Theta class) is 178–241 aa, GSTZ (Zeta class) is 219–238 aa and that of the unclassified (TGSTN) is 315–350 aa. Thus, the largest are the TGSTN (Supplementary Table S4).

*Tetrahymena thermophila* GST (TthGST) amino acid sequence identity matrices, after multiple alignment by ClustalW, show very diverse values: in TthGSTM the identity values are in the range 10%–99%, those of the TthGSTO class is 15%–91% identity, the range in TthGSTT is 30%–63%, in the two Zeta class (TthGSTZ) is 63% and the unclassified ones have only 16% identity. Among the putative TthGSTMs the most different sequences with respect to the rest are TthGSTM52, 53 and 54. With respect to nucleotide sequences, there are *TthGSTM* genes that are practically identical as well as their protein products, for example, *TthGSTM3*, *4* and *5* with an average homology among them of 97% and an average identity of 95%.

The average percentage of GSTM genes containing introns in *Tetrahymena* species is 22.55%. The Omega class genes show the highest average percentage (75.81%), while the GST genes from the Theta class have practically no introns (except for the *T. vorax* with 14% of its genes). About 50% of unclassified GST genes have introns. By contrast, all Zeta class GST genes have introns. The number of GST genes with introns in the rest of the analyzed ciliates is highly variable between classes and within the same class. Most of the *Tetrahymena* cGST genes with introns have only one intron, but some may have up to eight introns. All the other ciliate cGST genes analyzed have a much lower intron number (from one to three introns) (Supplementary Table S4).

### 3.2 Comparative analysis of domains among cytosolic TthGSTs

To exclude or confirm the true GST entity of the different presumed TthGSTs proteins shown on the TGD web site, regions identified by the PROSITE web server, as GSTs N-terminal (NTER) and C-terminal (CTER) domains (also called Domain-1 or -2, respectively) in the TthGST gene family are listed in Supplementary Table S5. From the 71 putative cGST paralogous genes, collected on the TGD website (http://www.ciliate.org), 59 presents both domains (NTER and CTER), except 6 that includes 3 of the Mu class (TthGSTM1, M2 and M19) and 3 of the Omega class (TthGSTO3, O5 and O8) which only present an NTER domain. In addition, 6 others, all from Mu class (TthGSTM31, M36, M40, M52, M53, and M54) have no recognizable GST domain. The average score of the NTER domains from the TthGSTM class has a value (19.30) approximately twice the value obtained by its CTER domains (9.42). This means that the NTER domain is more conserved than the CTER domain, which is more variable. It is also confirmed by the appearance of conserved motifs in the NTER domain of all TthGSTMs. The GSH-binding motif (G-site) presents a conserved amino acidic sequence (F/Y)PNLP(Y/F)(L/I)xxGD (where x can be one among six different amino acids), which is detected in all TthGSTMs (shaded yellow in Supplementary Table S5).

The average scores of the NTER and CTER domains of the TthGSTTs (Theta class) are very similar, with average values of 16.45 and 15.86, respectively. Conserved motifs are observed in both domains (shaded in yellow and blue respectively in Supplementary Table S5). The conserved SQPSR motif is typical of Theta GSTs and occurs in the NTER domains of all five TthGSTTs. Likewise, two highly conserved domains are detected in the CTER domains of this class of TthGSTs. The average score of the NTER domains of TthGSTO is slightly higher (14.92) than that of the CTER domains (11.00). Two conserved regions are present in the eight TthGSTOs, one of them (CP(Y/F) being the one that could be involved in GSH binding.

Within the NTER domain of the TthGSTZ active site, a highly conserved motif (SWRVRIAL) is detected among members of this GST class. Likewise, a conserved motif (18 aa) is observed in the CTER domain (blue shading). The average scores for both domains are quite similar (22.60 for the N-terminal and 20.02 for the C-terminal) (Supplementary Table S5).

Finally, in contrast to the rest of the putative TthGSTs, the average score of the CTER domain is slightly higher (17.03) than that of the NTER domain (13.78) in the unclassified GSTs (TthGSTNs). However, a conserved motif (IAELAGV) is detected in the NTER sequences, but there appear to be no conserved motifs in the CTER. In addition, in one of them (TthGSTN2) a third domain corresponding to Elongation factor 1 (EF-1) is detected (Supplementary Table S5).

### 3.3 Comparative structural analysis of TthGSTs

Supplementary Table S6 shows the different β-sheets/α-helix patterns, detected by the PSIPRED 4.0 web server (Buchan and Jones, 2019), in the NTER and CTER domains of each of the TthGST sequences registered on the web (http://www.ciliate.org). From the 54 putative TthGST (Mu class), registered as such on the web, there are 6 that do not have a recognizable GST NTER domain (as previously indicated, see Supplementary Table S5), nor do they show (green shaded names in Supplementary Table S6) β-sheets/α-helix patterns or even slightly like that NTER or CTER canonical GSTs. Among the remaining TthGSTs (65 in total), a whole variety of β-sheets/α-helix patterns exist. The most abundant pattern (68%) among all TthGSTs is the one containing the configuration β1α1β2α2α3β3β4α4α5 (light blue shading in Supplementary Table S6). This pattern appears in all cGST classes described in *T. thermophila*. The amounts of each class, from highest to lowest, are as follows: TthGSTM (27) > TthGSTO (2) = TthGSTT (2) > TthGSTZ (1) = TthGSTN (1). Three of them (TthGSTM7, M8 and M9) contain a sixth α-helix (α6) in the Domain-1, and six others (Mu class) are missing the fifth α-helix (α5) (Supplementary Table S6).

**FIGURE 1:**
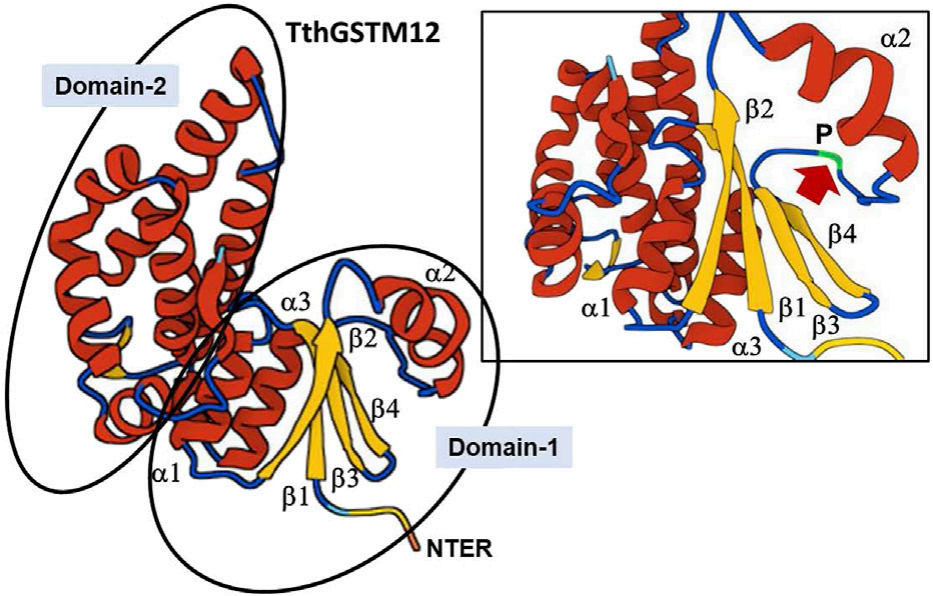
3D structure of the TthGSTM12 protein. The two domains are identified. The upper right box is an enlargement of Domain-1, with the thioredoxin canonical fold. The arrow indicates the cis-Pro-loop (for further explanation see text).

The next most abundant β-sheets/α-helix pattern (21%) present in the TthGSTs is β1α1β2α2β3β4α3α4 (shaded yellow in Supplementary Table S6), which contains the canonical thioredoxin folding pattern (β1α1β2α2β3β4α3). Only TthGSTM12 contains, in its NTER region (Domain-1), the canonical thioredoxin folding pattern without additional α-helixes (Supplementary Table S6). As an example, the inferred 3D structure from the TthGSTM12 amino acid sequence is shown in Figure 1. It shows in Domain-1 the thioredoxin canonical folding, with the three parallel beta-sheets (β1β2β4) and one antiparallel (β3). Between the α2-helix and the β3-sheet there is a loop containing a cis-Proline (P) residue (cis-Pro loop) highly conserved among almost all GSTs. Supplementary Table S5 shows (shaded in green) the location of this Proline (P) and depending on the β-sheets/α-helix configuration, this cis-Pro-loop is located between an α-helix and a β-sheet different from the canonical one.

The third most abundant (16.6%) pattern (shaded green in Supplementary Table S6) is β1α1β2α2α3β3α4α5 and appears in both Mu and Omega classes, and with one more α-helix (α6) in TthGSTO8 (Supplementary Table S6). A smaller number (2–4) of other β-sheets/α-helix patterns (differentiated by a colour code in Supplementary Table S6) are detected in the NTER of different TthGST classes. In the CTER regions of the TthGSTs the average number of α-helices is: 7 (TthGSTMs or TthGSTTs), 8 (TthGSTOs), 5 (TthGSTZs) and 6 (TthGSTNs) (Supplementary Table S6).

### 3.4 Transcription factor binding sites in the promoter regions of cytosolic *TthGST* genes

To expand our knowledge on TthGSTs at the level of transcriptional regulation, the number of potential binding sites for selected groups of transcription factors (TFs) to each of the TthGSTs is shown in Supplementary Table S7. Two classes of TFs have been chosen: bZIP and C4 zinc finger-type (Supplementary Table S7). Within the bZIP class, the average number of potential binding sites per gene for the c-Jun and c-Fos TFs is 3, representing approximately 32%. For the Nrf2/MafK tandem it is between 1 and 2 sites/gene on average (∼18%). For Jun B and Jun D it is between 1 and none (representing about 8%). The TthGST genes with the highest number of potential motifs for these TFs are: *TthGSTM43* and *TthGSTT1*.

Regarding C4 zinc finger-type class, four types of GATA TFs have been chosen (Supplementary Table S7). The highest average number of potential binding sites is shown by GATA-1 (14 per gene), representing ∼71%. GATA-2 and GATA-3 are in second and third place, with 18.6% and 10.9% respectively. Potential sites for GATA-6, in the promoter region of the TthGST genes, are very few (one site in only three genes) (0.2%) (Supplementary Table S7). *TthGSTO6* and *TthGSTT2* are the genes with the highest number of motifs for this type of TFs.

### 3.5 Phylogenetic analysis of cytosolic TthGSTs

**FIGURE 2:**
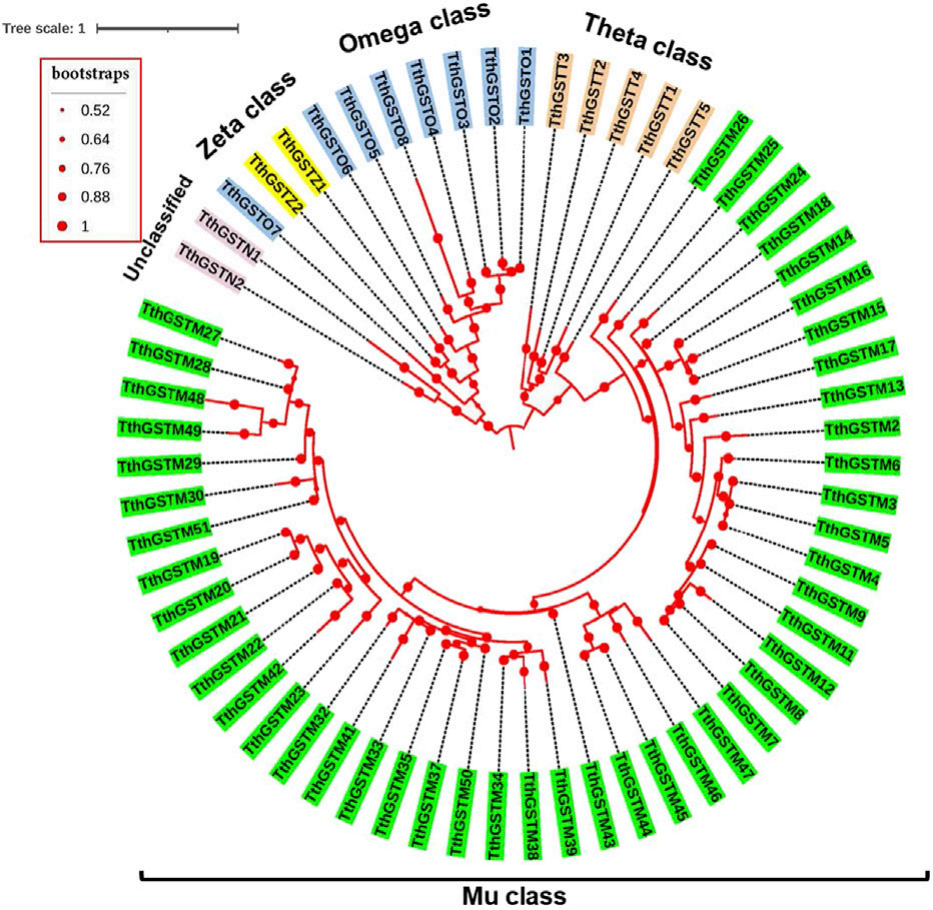
Circular phylogram of the TthGSTs. Each of the five GST classes is distinguished by different colors. Each branch length follows the scale. Calculated bootstrap values, from 2000 replicates, are indicated as spheres of different sizes (values from 0.52 to 1).

Figure 2 shows the circular phylogram of the TthGSTs that excludes the TthGSTM1 and TthGSTM10 sequences because they have too large distances. The 5 classes of TthGSTs are distributed in 5 well-defined groups with a common origin. The two Z class members (TthGSTZ) seem to be connected to the omega class, and the unclassified ones (TthGSTN) have a common origin with the omega and zeta class group (Figure 2). Within each class, many TthGSTs appear to arise from gene duplications (see TthGSTO and TthGSTT classes or some groups from the TthGSTM class). In a non-circular phylogram representation (Supplementary Figure S2) it is best seen that a group of 19 TthGSTM genes are phylogenetic predecessors of members from the omega, theta and zeta classes.

### 3.6 General features of MAPEG GSTs from *Tetrahymena* species

The average number of genes encoding putative MAPEG GSTs in *Tetrahymena* species is 2, ranging from 1 to 4, whereas in the rest of the analyzed ciliates the range of MAPEG genes is much wider (1–11) (Supplementary Table S8). MAPEG genes with introns in *Tetrahynena* species are practically zero, in contrast to the ciliates analyzed, whose average number is 2. In the latter, the average number of introns/gene is 2 (Supplementary Table S8).

The analysis of the molecular structure of the MAPEG from the different *Tetrahymena* species shows that the predominant structures are α-helixes, with an average of 6 from which 4 are transmembrane helixes (Supplementary Table S9). As an example, the inferred 3D structure of TthMAPEG2 is shown in Supplementary Figure S3.

The alignment of all MAPEG protein sequences from *Tetrahymena* species shows that this family is divided into two well-defined groups or clans (Supplementary Figure S4), this is also reflected in the phylogram in Supplementary Figure S5. Clan1 includes 11 sequences (3 from *T. thermophila* and 8 from each of the other species analyzed), while clan2 contains 9 sequences (2 from *T. paravorax* and one from each of the other species except for the MAPEGs from *T. empidokyrea* and *T. shanghaiensis*, which are the only ones in clan1 (Supplementary Figures S4, S5). Both clans have the MAPEG family’s conserved signature motif (RxxxNxxE/D) (shown in Supplementary Figure S4 within a box).

### 3.7 Quantitative expression analysis of selected TthGST genes under different abiotic stressors

**FIGURE 3:**
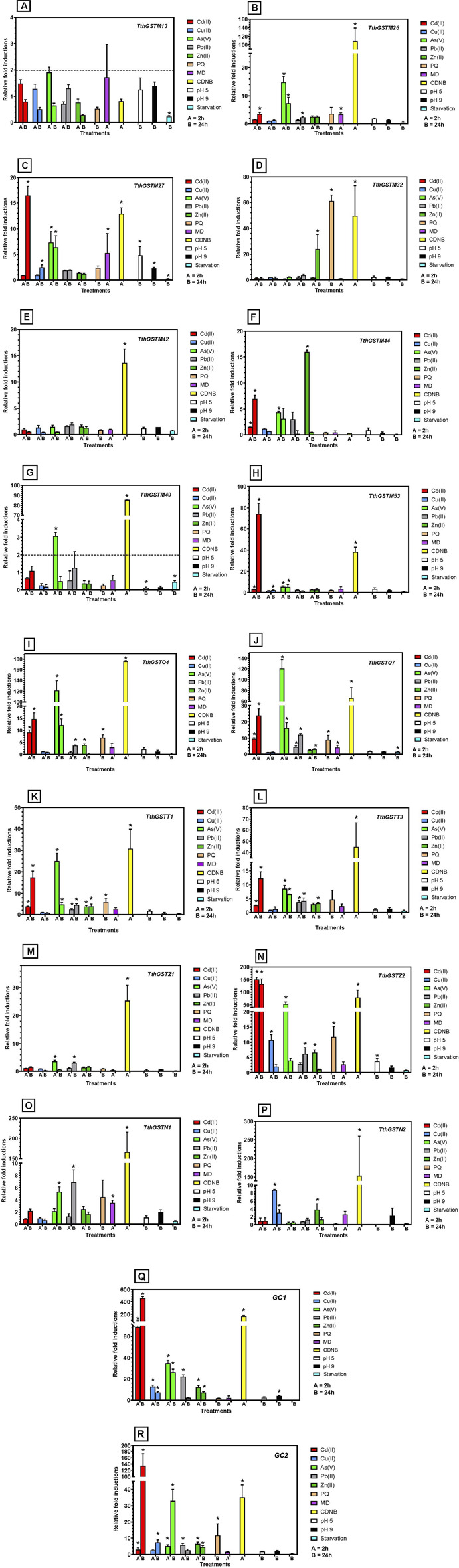
Quantitative expression analysis of selected TthGST genes under different abiotic stressors. Each treatment is identified by color. Stars indicate significant difference from control (p < 0.05). Each panel **(A–R)** represents the results for each TthGST gene analyzed (gene name is indicated in the upper right margin of the panel). For further information see text.

The selection of these *TthGST* genes was random, although some of them had already been analyzed in previous studies. In contrast, the abiotic stressors and concentrations chosen for this analysis were basically the same as those used in previous works, to facilitate experimental conditions for a comparative analysis. The selected genes are: 7 *TthGSTM* genes (M13, M26, M27, M32, M42, M44, M49 and M53), 2 *TthGSTOs* (O4 and O7), 2 *TthGSTTs* (T1 and T3), the 2 *TthGSTZs* (Z1 and Z2), the 2 *TthGSTNs* (N1 and N2) and two gene clusters (GC) including 3 *TthGSTM* genes each (GC1 = M3, M4 and M5, GC2 = M14, M15 and M16) that being practically identical (99%–100% or 96%–97% nucleotide sequence identity, respectively), and due to the impossibility of designing primers to differentiate them, they have been tested together, so their expressions are the total sum from all of them or from one or two of them. Figure 3 shows the results of the relative induction of each of these genes or gene clusters under the stress of various abiotic agents.

The *TthGSTM13* gene (Figure 3A) is the only one that, under all stressors, has induction values below 2, so it is not considered a positive gene induction value. In contrast to the rest of the selected genes, *TthGST42* is only significantly expressed (about 13-fold) with the GSTs inducer (CDNB) (Figure 3E), and there are two genes (*TthGSTM13* and *M44*) that are not significantly expressed with CDNB (Figures 3A, F). All the others (88%) are significantly expressed with this GST inducer. Starvation, in general, does not induce the expression of these genes (always < 2-fold) although in some cases there is a significant difference with respect to the control.

The genes that are significantly induced with a greater number of different stressors (7–11) are *TthGSTM27*, *O4*, *O7*, *T1*, *Z2* and the GC1 and GC2 groups (as several genes are expressed together). In contrast, those that are significantly induced by the fewest number of stressors (1–4) are *TthGSTM32*, *M42*, *M44*, *M49*, *Z1*, and *N2* (Figure 3).

With respect to metal(loid)s, at least 9 *TthGST* genes of the selected ones are significantly overexpressed by Cd(II), or 15 genes if we consider that the 6 from the GCs are all expressed, regardless of exposure time (Figure 3). Four or 12 (considering the GCs) are significantly induced by Cu(II), 12 or 18 (considering GCs) by As(V), 7 or 13 by Pb(II) and 8 or 14 by Zn(II). PQ induces 5 or 8 of the selected genes. MD significantly induces only 4 genes (*TthGSTM26*, *M27*, *O7*, and *N1*), after 2 h treatment. A pH 5 significantly induces (>2-fold) only two genes (*TthGSTM27* and *Z2*), whereas pH 9 induces only one gene (*TthGSTM27*) or 4 (if we consider GC1). Starvation does not induce any of the selected *TthGST* genes (value > 2-fold) (Figure 3).

## 4 Discussion

### 4.1 Comparative analysis of *T. thermophila* cytosolic and MAPEG GSTs

In the *Tetrahymena* complex there are species (such as *T. thermophila*, *T. pyriformis* or *T. vorax*) with a total number of putative cytosolic GST genes (Table 1) that exceeds the average number found in mammals (15–30) or plants (40–60) (Frova, 2006). This is another example of the ciliate macronuclear genome organization in gene families, consisting of numerous paralogous genes (Aury et al., 2006; Eisen et al., 2006; Mozzicafreddo et al., 2021; Swart et al., 2013). A common feature among parasitic protozoa is to have a low number of genes encoding GSTs. Probably, the condition of being an intracellular parasite does not require a specific defense against environmental xenobiotics.

As is generally the case in mammals, in the genus *Tetrahymena*, Mu class GSTs predominate (with a ∼69% average of the total), thus being equally specific to this group of eukaryotic cells. With mammals they also have in common the Theta, Omega and Zeta classes (Frova, 2006). Although less abundant, they are also present in other ciliate species, where the Theta and Sigma classes tend to predominate (Table 1). The percentages of the different known GST classes in the selected ciliates are as follows: the Mu class is present in all of them (100%), the Theta class in 94.7% of them, Omega in 78.9%, 68.4% have the Zeta class, Sigma a 36.8%, Delta-Epsilon 26.3%, Tau and Alpha a 5.2%. In addition to these classes there are others GST which we initially considered as unclassified or not included in any class (UC or N in this paper) which are present in 63% of the analyzed ciliates. Some of these classes are ubiquitous (even among the different kingdoms), while others are taxonomic group or species specific.

A comparative analysis of the domains of the GSTNs has shown (Supplementary Table S10) that there are two classes among the *Tetrahymena* species, and each of them usually presents one of each class. One of the classes contains both GST-NTER and -CTER domains, with a size and an average molecular mass (219 aa and 24.56 KD on average) very similar to canonical GST classes (Koirala et al., 2022). It also has the cis-Pro-loop residue typical of thioredoxin superfamily proteins (Gamiz-Arco et al., 2019), and present in other classes of GSTs (Supplementary Table S5). These GSTNs have several conserved motifs, such as EFxxKxPLG in NTER or DQ(Y/F)(L/I)D in the CTER of GST domains (see alignment in Supplementary Figure S6), which are very different from those found in the known GST classes. Therefore, we can consider that this class (GSTN) is specific to the *Tetrahymena* complex (set of *Tetrahymena* species), just as PteGSTN1 and EvaGSTN1 might be specific to these other ciliates. The presence of unclassifiable GST has also been reported in other organisms, such as marine invertebrates (Park et al., 2020), or insects (Koirala et al., 2022). In the protozoan parasite *P. falciparum* (Hiller et al., 2006), and in the ciliate *B. japonicum* two unclassifiable isoforms have been reported (Takada and Matsuoka, 2008).

The second class of GSTNs in *Tetrahymena* species have, in addition to the two domains GST-NTER and -CTER, a third domain EF1G-CTER (Elongation factor 1 (EF-1) gamma C-terminal domain profile). This makes these proteins larger and with higher molecular mass (420 aa and 47.71 KD on average) (Supplementary Table S10). In this case it is not a new GST class, since there are GSTs with an EF1G domain described in other organisms (Koonin et al., 1994). As can be seen in Supplementary Figure S6, the EF1G domains of the GST-EF1Gs from *Tetrahymena* species have many modules that are almost identical to each other. This separation of the two classes (GSTN and GST-EF1G) is also corroborated in the phylogram shown in Supplementary Figure S7. This circular phylogram shows the two separate classes (GSTN and GST-EF1G) with a common precursor, together with those of other ciliate species which also have one of each type. Regarding the unclassified GSTs from the selected parasitic protozoa, there are those with both domains (NTER and CTER), with a single domain (NTER or CTER) or even with four domains (2NTER and 2CTER) (Supplementary Table S10).

After an analysis of the 54 Mu class GSTs reported in TGD website for *T. thermophila*, we appreciate that 6 of them do not seem to be GSTs, so the total number is reduced to 48 and the total number of GSTs of this species would be 65 instead of 71 (Table 1). The reasons for this statement are as follows: a)- The alignment of all Mu class TthGSTs (Supplementary Figure S8) reflects that there are important differences in the amino acid sequence of these six putative GSTs with respect to the rest. The lengths of their amino acid sequences (596 aa for M31, 415 for M36, 1,400 for M40, 76 for M52, and 199 aa for M53 and M54) are very different from the average range from the rest TthGSTM class (211–248 aa). There are significant differences in the most conserved domains (such as the G-site) with the rest of TthGSTMs (Supplementary Figure S8) or the canonical Mu class GSTs (Park et al., 2020). b)- The PROSITE web server does not identify any GST domains for these 6 sequences unlike the rest (Supplementary Table S5), and c)- They show very different β-sheets/α-helix patterns in their NTER and CTER regions compared to TthGSTMs (Supplementary Table S6).

*In silico* analysis of the 48 cytosolic TthGSTs has revealed their correct inclusion in the different known 4 classes, together with the confirmation of being real GSTs. However, not only do they show similarities with canonical GSTs, but they also show differences to be highlighted. We summarize both in the following points: 1- In contrast to the mean identity percentage (>40%) established to consider a GST protein within the same class, some of the *Tetrahymena* GSTs of the same class present lower identity values between them, with a much wider range. The same is true for other organisms (Frova, 2006). 2- The number of genes with introns in the four cGST classes from *Tetrahymena* species presents the following ranking: Zeta (100%) > GSTN (80%) > Omega (75.8%) >> Mu (22.5%) > Theta (0 or 14%). In general, the number of GST genes with introns is usually low, however there are some organisms in which all their GST genes have introns, as in human GSTs (4–8 introns) (Jowsey and Hayes, 2010).

In vertebrate GST transcripts, the existence of so-called alternative splicing has been reported (Wongsantichon and Ketterman, 2005). This mechanism generates different protein isoforms from the same gene, by differential incorporation of exons into the definitive mRNA. Therefore, this mechanism can generate functional heterogeneity from a limited number of GST genes, mainly in Domain-II (CTER) where the amino acids that bind different xenobiotics reside, so that the variability originated in this domain would allow GSTs to recognize a great variety of xenobiotics. When there is a lack of introns, as in some classes of *Tetrahymena* GSTs (such as the Mu class), and alternative splicing is hindered, an increase in the number of paralogous genes could guarantee greater functional heterogeneity.

3- TthGSTs exhibit a wide variety of β-sheets/α-helixes patterns, up to 8 different ones (Supplementary Table S6). The most abundant one, present in all classes although mostly in the Mu class, is similar, but not identical, to the canonical thioredoxin folding pattern. In these the α2 and α3 helixes are contiguous, and are not separated by two β-sheets, and have two extra α-helixes. The canonical thioredoxin folding pattern is also present in 10 TthGSTs (6 Mu, 2 Ω, 1 Zeta and 1 GST-EF1G or GSTN2), with an additional α-helix (α4) (Supplementary Table S6). Domain II or CTER of TthGSTs present an average of 6 α-helixes, which is within the range (4–7) described for canonical GSTs from many organisms (Frova, 2006).

4- The eight members of the TthGSTO class are Cys-GSTs (GSTs containing Cys in their catalytic site) as occurs in other organisms (from bacteria to mammals). They display the conserved CP(Y/F) motif (Supplementary Figure S9) that also appears in other mammalian, plant and algal Cys-GSTs (Lallement et al., 2014).

5- All five members of the TthGSTT class have a motif (SQPS) that is highly conserved among the Theta class GSTs (Vaish et al., 2020) (Supplementary Figure S10). The two TthGSTZs have a conserved motif [SSxSWRVR(I/L)AL] very similar to those of the Zeta class (SWRVRIAL) (Park et al., 2020), with the serine residue (S) in the active site and two extra serine residues (Supplementary Figure S11).

Microsomal or MAPEG GSTs are ubiquitous proteins, present in mammals, plants, fungi and bacteria (Bresell et al., 2005). The MAPEG family, according to Jakobsson et al. (2000) when comparing humans with other prokaryotic and eukaryotic organisms, can be subdivided into four subgroups or subfamilies; group-I (includes 3 humans), group-II (1 human and 4 between plants and fungi), group-III (2 from bacteria) and group-IV (2 from the 6 humans). In *Tetrahymena* species there are two well-defined groups, clades or subfamilies (Supplementary Figures S4, S5). Most (70%) have two members, one in each clade, except for those with only one member (such as *T. empidokyrea* or *T. shanghaiensis*) which are both in the first group or *T. paravorax* in which both are in the second group. *Tetrahymena thermophila* has four MAPEG GST isoforms, three of them in the first group and one in the second. However, despite the differences in their amino acid sequences, both groups present the conserved MAPEG-GSTs family motif (RxxxNxxE/D) (Jakobsson et al., 1999).

The bZIP (basic leucine zipper) superfamily of transcription factors (TFs) is one of the oldest and most conserved among eukaryotes (Jindrich and Degnan, 2016). These TFs are involved in the cellular response to different environmental stressors, such as heat shock, changes in osmolarity, the presence of toxic compounds or pathogens (De Francisco et al., 2018; Sornaraj et al., 2016). The bZIP superfamily includes about seven families (Yang and Cvekl, 2007), and among them is the AP-1 family, which includes the Jun (v-Jun, c-Jun, JunB, and JunD), Fos/Fra (v-Fos, c-Fos, FosB, Fra1, and Fra2) and CNC (Nrf1, Nrf2 and Nrf3) subfamilies.

Potential binding sites for some of these TFs have been located in the promoter regions from cytosolic TthGST genes. Regardless of TthGST class the average largest number of potential binding sites per gene (from Jun and Fos subfamilies) is for c-Jun or c-Fos (∼32% for each). Binding sites for c-Jun are present in 64% of TthGST paralogous genes, and for c-Fos is about 66%. For each TthGST gene there are the same number of binding sites for c-Jun as c-Fos (Supplementary Table S7) since both upon binding form the early response AP-1 TF. AP-1 dimers are one of the most universal TFs related to the eukaryotic cellular stress response to a wide range of toxins (Wisdom et al., 1999). Four highly conserved AP-1 TFs have been characterized in several *Tetrahymena* species and appear to be involved in the upregulation of *T. thermophila* metallothionein gene expression, which are induced under toxic metals among other environmental stressors (De Francisco et al., 2018). It is therefore not surprising to find potential binding sites for these TFs in the promoters of GST genes, which are also induced by environmental stressors, as has also been previously reported (Daniel, 1993).

Although the average number of binding sites for the Nrf2/MafK tandem is lower (∼18%), the number of TthGST gene promoters possessing it is considerably higher (83%). This TF has also been linked to the oxidative stress cellular response and xenobiotic detoxification (He et al., 2020; Niture et al., 2010), and similarly associated with the overexpression of GST genes (Ikeda et al., 2004; Tonelli et al., 2018). The average number of Jun B or Jun D binding sites is the lowest (∼8%), but 61% of TthGST genes possess it. The latter two have not been usually reported among TFs linked to GST genes, so they are probably in the minority.

The vertebrate family of GATA TFs comprises six types (GATA1-6), which in turn are divided into two subfamilies: GATA-1,2,3 and GATA-4,5,6 (Lentjes et al., 2016). In all TthGST gene promoters, binding sites for GATA-1 and -2 are detected, with averages of 71.6% and 18.6%, respectively. GATA-3 appears less frequently (10.9%) and in ∼77% of the TthGST genes. Binding sites for GATA-6 are virtually absent, except for 3 (4.6%) TthGST isoforms (two Mu and 1 Theta) (Supplementary Table S7). GATA TFs are involved in the cellular response to environmental stress (Abdulla et al., 2024), xenobiotics (Jin et al., 2023), oxidative stress (Hu et al., 2017) or during development and disease (Lentjes et al., 2016). Several studies have associated the GST gene expression upregulation with GATA TFs, for example, human GATA-1 and GSTP1-1 (Schnekenburger et al., 2003), plant GSTs and GATA motifs (Chandra and Leon, 2022), TaGSTU3 and a GATA box (Pandey et al., 2012) or Tau-class GST and GATA boxes (Tiwari et al., 2016). GATA motif clusters in the promoter region of the *T. thermophila* HSP70-1 gene involved in the thermal stress response have been reported (Barchetta et al., 2008). Likewise, a GATA element has been implicated in the gene expression of the *T. thermophila* metallothionein MTT5 under cadmium stress (Formigari et al., 2010). This analysis of the promoter regions of TthGST genes and the presence of similar TFs linked with GST genes from other organisms corroborates the role of these genes in the response to environmental stressors.

### 4.2 Phylogenetic considerations and implications of the presence of Mu-class GSTs in ciliates

Enzymes involved in detoxification processes, such as GSTs, have existed in both prokaryotes and eukaryotes for more than about 2,500 million years (Nebert and Dieter, 2000). GSTs constitute a very ancient protein superfamily, which evolved from an ancestral thioredoxin-like protein in response to oxidative stress (Sheehan et al., 2001).

In many different organisms it has been reported (Harari et al., 2020; Low et al., 2007; Monticolo et al., 2017; Park et al., 2020) that different GST classes and members of the same class arose by successive and extensive gene duplications and subsequent divergence, some conserving a high homology, giving rise to numerous paralogous genes or isogenes located in close clusters on the same chromosome. Something similar has also occurred in the *T. thermophila* GST superfamily, and most likely in other species of this genus. Clear examples of recent gene duplications are shown by members from the TthGSTT and TthGSTO classes (Figure 2 and Supplementary Figure S2). Within the TthGSTM class we have several examples of duplicated genes located in the same cluster on the same chromosome arm, such as TthGSTM3, M4 and M5 cluster on the right arm of the micronuclear metacentric chromosome 2 (2R), the TthGSTM14, M15 and M16 cluster on the right arm of chromosome 1 (1R) or TthGSTM44, M45, M46 and M47 cluster on the telocentric chromosome 5 (Dede and Arslanyolu, 2019).

An evolutionary model for GSTs has been proposed, reviewed by Frova (2006), in which the idea that thioredoxins/glutaredoxins are the ancestors of all soluble (cytosolic and mitochondrial) GSTs predominates. The pathway followed by cytosolic GSTs would start from a monomeric prokaryotic glutaredoxin-like ancestor (such as *E. coli* GRX2), from which Lambda-class GSTs, intracellular chloride channels (ICLCs) and dehydratoascorbate reductases (DHARs) would arise. After a dimerization stage (GSTs act as homo- or heterodimers), Omega- and Beta-class GSTs originated, which maintained cysteine as the active residue. The next evolutionary stage, according to this model, would be to move from cysteine to serine chemistry. The Phi and Tau (plant-specific) and Delta (insect-specific) classes arose later because they were supposedly considered specific to a phylogenetic group of organisms. The next stage marks an evolutionary separation of the mammalian GSTs classes (Alpha, Mu and Pi) and the Sigma class by changing the serine residue to a tyrosine in the catalytic GSH-binding region (G-site).

Ciliate protozoa date back to the Proterozoic (paleo-/meso-Proterozoic) 10^9^ years ago, so they are older than fungi and, of course, vertebrates, so the Mu class GSTs are much older than assumed in the current model. The Mu class is present in most ciliate protozoa, so they are no longer exclusive to mammals, moreover other putative phylogenetic group-specific classes are not, since they also appear in some ciliates (Table 1). According to the currently accepted evolutionary model (Frova, 2006) the catalytic site transition was Cys --> Ser --> Tyr (Omega --> Theta/Zeta --> Mu). But in the putative catalytic sites (or very close to them) in the TthGSTs there are tyrosine (Y) residues in all four classes (Mu, Omega, Theta and Zeta), in addition to cysteine (C) in the Omega or serine (S) in the Theta and Zeta classes (Supplementary Figures S8–S11). In addition, and if we consider the phylogram (Supplementary Figure S2) of the TthGSTs, there is a fraction of TthGSTM molecules that can be considered ancestors of the TthGSTO, TthGSTZ and TthGSTT classes. All this means that, after incorporating the GSTs of ciliate protozoa, we must consider another evolutionary model for GSTs. Interestingly, in marine organisms (rotifer and copepods) a phylogenetic analysis of their GSTs locates the Omega and Signa classes as the most recently diverged, while the Mu class arises early (Park et al., 2020).

### 4.3 On the induction of TthGST gene expression under abiotic stressors

GST gene expression is induced by both endogenous and exogenous factors. Endogenous factors include tissue-specific, sexual factors or different developmental stages, and are involved in signaling pathways, S-glutathionylation of proteins, glycolysis, DNA repair, autophagy and multiple diseases (Eaton and Bammier, 1999; Lv et al., 2023). And among the exogenous or external agents inducing GST gene expression are organic xenobiotics, metal (loid)s, a wide variety of drugs, oxidative stress inducers, etc. (Raza, 2011; Strange et al., 2000; Vaish et al., 2020; Wagner et al., 2002).

Before this study, the expression of some TthGST genes, along with different ones, under stress by metal(loid)s (Alonso et al., 2024; Rodriguez-Martin et al., 2022; Romero et al., 2019) and organic xenobiotics (Díaz et al., 2016; Feng et al., 2007; Kapkac and Arslanyolu, 2021; Miao et al., 2006) has been analyzed. Likewise, some of these TthGST genes have already been detected in gene libraries, transcriptomic (Miao et al., 2009) or proteomic studies (De Francisco et al., 2023), under different treatments.

Table 2 summarizes graphically the expression induction patterns of the 16 TthGST genes analyzed individually or 22 if we add the two clusters of 3 genes each analyzed together by qRT-PCR, under the action of 11 abiotic stressors (2 or 24 h treatments). As expected, most (88%) are significantly induced by CDNB (substrate and inducer of GSTs) except for TthGSTM13 and TthGSTM44. The former could be a pseudogene, since it is not significantly expressed by any of the stressors used (Table 2), although it could be induced by others not yet tested. In a microarray carried out at different *T. thermophila* cell cycle stages (Miao et al., 2009), this gene (M13) appears to be expressed at a middle level during vegetative growth, at the beginning of a starvation period (up to 3 h) and at some late stages of conjugation, therefore it cannot be considered a pseudogene. M13 and M18 are located close together on the same macronuclear chromosomal fragment (Dede and Arslanyolu, 2019), with 92% nucleotide homology between them, and M18 shows low expression levels on the microarray restricted to the vegetative growth phase (Miao et al., 2009). TthGSTM44 is only expressed with Cd (24 h), As (2 h) or Zn (2 h) (Table 2), and in the microarray (Miao et al., 2009) it shows a very low or basal expression throughout the biological cycle. Therefore, it can be considered as metal(loid)s specific, mainly Zn.

**TABLE 2:** Comparative analysis of gene expression induction patterns of selected cytosolic TthGST genes analyzed by qRT-PCR.

Treatments		*TthGST* genes																			
		*M13*	*M26*	*M27*	*M32*	*M42*	*M44*	*M49*	*M53*	*O4*	*O7*	*T1*	*T3*	*Z1*	*Z2*	*N1*	*N2*	GC1	GC2	Total^a^	%
Cd(II)	2h																			8	44.4
	24h																			11	61.1
Cu(II)	2h																			3	16.6
	24h																			5	27.7
As(V)	2h																			11	61.1
	24h																			9	50
Pb(II)	2h																			4	22.2
	24h																			7	38.8
Zn(II)	2h																			7	38.8
	24h																			6	33.3
PQ	24h																			6	33.3
MD	2h																			4	22.2
CDNB																				16	88.8
pH 5	24h																			3	16.6
pH 9																				2	11.1
Starv.																				0	0.0
Total^b^		0	4	7	4	1	3	2	5	8	10	9	8	3	9	3	4	11	10		

Shaded boxes indicate significant (p < 0.05) induction values greater than 2-fold with respect to the control (no treatment).

^a^ The last two columns show the number and percentage of selected TthGST, genes (or clusters) significantly induced by the same stressor.

^b^ The last line shows the total number of significant inductions for the same TthGST, gene (or cluster) originated by the different treatments. GC1: Gene cluster *M3/M4/M5*. GC2: Gene cluster *M14/M15/M16*.

At the opposite extreme we have TthGSTM42 which is only expressed with CDNB (Table 2), and this same gene in the microarray is expressed at medium-low levels in some life cycle stages (mainly during vegetative growth) (Miao et al., 2009). Cd (24 h) and As (2 h) treatments induced the highest number of the chosen TthGST genes (61%), with O4, O7, T1 and T3, together with clusters GC1 and GC2 being significantly expressed by both metal (loid)s (Table 2). At lower concentrations (10 μM) TthGSTO4 and O7 genes are also induced with both As(III) and As(V) (Rodriguez-Martin et al., 2022). TthGSTO7 expression is also induced with selenite (30 μM, 24 h) and selenate (20 mM, 24 h) (Romero et al., 2019), and with the herbicide Paraquat (PQ) (Díaz et al., 2016). A proteomic analysis in a *T. thermophila* strain adapted to elevated Pb concentrations has reported that TthGSTO4 is the most abundant Omega class GST protein (2.9-fold the control), along with TthGSTO3 (2.3-fold the control) (De Francisco et al., 2023). The individual TthGST genes from the Omega and Theta classes seem to be the ones induced by a higher number (8–10) of very different stressors tested, as in the GC1 or GC2 clusters it would be the result of the sum of several of them or any of the three in the cluster (Table 2).

With respect to the Omega TthGSTs, which are Cys-GSTs (Supplementary Figure S9), it could be argued that the presence of the cysteine residue in the catalytic site could give these molecules a similar capacity to thioredoxins and glutaredoxins, protecting the cell from oxidative stress produced by very diverse inorganic or organic compounds (Kim et al., 2017). Although the actual function of these residues in these enzymes, and their potential ability to transfer GSH, is still a mystery (Lallement et al., 2014). On the other hand, TthGSTs from the Theta class have serine residues in their catalytic sites (Supplementary Figure S10) and also exhibit a broad response to many different stressors. Interestingly, neither the omegas (O4 and O7) nor the thetas (T1 and T3) tested are induced by copper (Table 2), which is an oxidative stress agent. Although copper toxicity could be blocked by overexpression of copper-specific metallothioneins (such as the MTT2/MTT4 pair) present in this ciliate (Gutierrez et al., 2024), and being an essential metal, which is less toxic, it would not need to enhance cellular defenses with GSTs.

TthGSTZ2 also exhibits a broad response to many different stressors and has serine residues in its active site (Supplementary Figure S11). This same gene is also induced under other stressors not used here, such as europium oxide (Alonso et al., 2024), Se(IV) and Se(VI) (Romero et al., 2019).

Other analyzed TthGST genes respond to a low number (1–3) of different stressors, such as M42 (already mentioned previously), M44, M49, Z1 or N1 (which only respond to 3 different stressors) (Table 2). The M49 protein is one of the three most abundant GSTs in the control strain with respect to the Pb-adapted strain (De Francisco et al., 2023). M44 does not appear to be expressed during the cell cycle phases studied in the microarray (growth, starvation or conjugation), and Z1 shows a medium expression exclusively during vegetative growth (Miao et al., 2009; Xiong et al., 2013). The stressors that induce a lower number of selected TthGST genes are copper, acid or basic pH and starvation.

The induction value ranking representation of the selected TthGST gene expression against the different stressors (Table 3) shows the following: 1- TthGSTZ2, O7, O4, T1 and T3 are the most expressed genes with the highest levels against the tested metal(loid)s (mainly cadmium, arsenate and lead). 2- Under Zn stress, M44 and M32 with the highest induction values are added to the five mentioned above. 3- TthGSTM27 is the one that reaches the highest induction values under MD and is one of the few that is expressed under acid or basic pH. 4- TthGSTN2 is specifically induced against essential metals (copper and zinc) and CDNB (GST inducer). Likewise, this GST-EF1G gene is the most expressed (2-fold compared to the Pb-adapted strain) under normal (control) vegetative growth conditions (De Francisco et al., 2023), which is consistent with its response to essential metals habitually present in the cell.

**TABLE 3:** Gene expression induction value ranking of the selected cytosolic TthGST genes under different treatments.

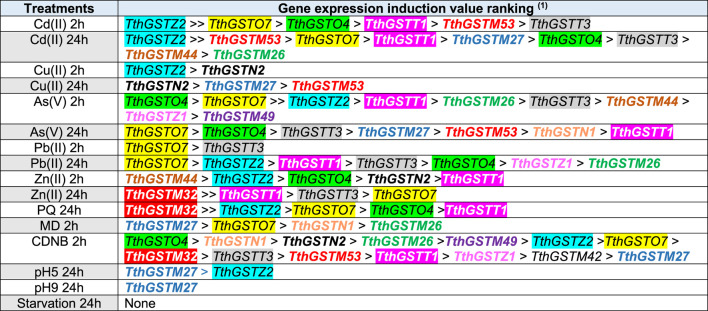

^a^ Only significant (p < 0.05) induction values greater than 2-fold to the control are shown. The occurrence of the same gene two or more times is indicated by a color code for each gene.

Some examples of the relationship between metal(loid)s and GSTs from other organisms are as follows: the yeast *Schizosaccharomyces pombe* has 3 GST genes (I, II and III), and Cd induces the expression of GST-I and -III, the latter is also induced by Hg, Al, Zn or Cu (Shin et al., 2002). In the blowfish *Takifugu obscurus*, the expression of 7 GST genes under Cd stress has been studied, and 5 of them (Mu, Omega, Zeta, Theta and MAPEG classes) are induced after 6 h exposure (Kim et al., 2010). A qRT-PCR study of several GST gene expressions in the marine copepod *Tigriopus japonicus* under Cd revealed that one GST gene (Sigma class) is the most highly expressed after 12 h of treatment (Lee et al., 2008). Mycorrhizal fungi respond to metal stress by inducing GST gene expression (Hildebrandt et al., 2007). In general, metals block animal and plant GSTs, so their inactivation would require more enzyme and an induction of the corresponding gene expression (Dobritzsch et al., 2020).

Oxidative stress originating from PQ or MD induces the expression of GST genes, such as TthGSTO7 and O4 (Table 3). In a *Caenorhabditis elegans* worm transgenic (Burmeister et al., 2008), the GSTO-1 gene (Omega class) was overexpressed, leading to increased resistance to PQ. Likewise, blocking this gene (using iRNA) showed increased sensitivity to PQ. The optimum pH for the GST catalytic activity is around neutrality.

The optimum pH for the GSTs catalytic activity is around neutrality. One of the few cases where the expression of a GST gene (Mu class) under pH stress has been studied is in the white shrimp *Litopenaeus vannamei* (Zhou et al., 2009). Both the expression of this gene (LvGSTM) and its enzymatic activity increased with respect to the control after 12 h exposure to pH 5.6. However, it does not increase its expression at pH 9.3 (Zhou et al., 2009). Our results show that, among the selected genes, TthGSTM27 is the one that is most expressed under acid or basic stress. Genes encoding antioxidant enzymes (superoxide dismutase, catalase, glutathione peroxidase) are overexpressed under acid or basic pH stress (Zhang et al., 2015).

Regarding the expression of the GC1 and GC2 clusters, each of which contains three Mu class genes, there are only three differences between them; under Cu (2 h) and pH 9 only those from GC1 are expressed, while under PQ only those of GC2 are expressed. Both gene clusters are located together on chromosomes 2 and 1 respectively, which may favor a regulation of their expression by the same elements (some of them have similar potential transcription factor binding sites in their promoter regions, Supplementary Table S7). Both gene clusters show very high levels of expression against mainly cadmium and arsenate (Figure 3), but during the cell cycle their expressions are very low (Miao et al., 2009).

The gene evolution by duplication and subsequent divergence is one of the most universal mechanisms for the creation of new genes and the origin of many gene families. The term ecoparalog (Sánchez-Pérez et al., 2008) applies to genes (from the same organism) that are similar in their sequences, but the expression of each paralogous gene is induced by different environmental agents. Ciliate genome sequencing has shown that these microorganisms are a paradigmatic example of genetic redundancy, which is considered to confer to the organism a certain degree of robustness, since it can maintain a stable phenotype under environmental changes. The TthGST superfamily is a good example of genetic redundancy and ecoparalogous genes, thus the TthGSTM27 and M53 pair (with 94% amino acid sequence identity, and located on the same chromosome) are differentially expressed under MD stress and acidic or basic pH, or the TthGSTZ1 and Z2 pair (with 84% amino acid identity and located on the same chromosomal arm 4L) show differential expression patterns for different stressors (Cd, Zn, Cu, PQ or acidic pH).

## 5 Conclusion

1- The TthGST superfamily *in silico* analysis implies fundamental changes in several aspects related to the putative TthGST genes and GSTs in general: a)- the list of potential GST genes in the genome web of this ciliate should be modified. b)- the significant diversity of *T. thermophila* β-sheets/α-helix patterns, and probably from other ciliates, should be considered as other feasible possibilities independent of the canonical thioredoxin pattern. c)- the current GST phylogenetic model should be reconsidered after taking into account the ciliate GSTs, organisms more ancient than vertebrates.
2- The expression results of selected TthGST genes show that an individual differential expression exists depending on the environmental stressor to which it is exposed and independently of the GST class. Many of them respond to the two most toxic metal(loid)s used (Cd or As).

## Data Availability

The original contributions presented in the study are included in the article and supplementary material. Further inquiries can be directed to the corresponding author.
