# Supplementary material for: Tetrahymena thermophila glutathione-S-transferase superfamily: an eco-paralogs gene network differentially responding to various environmental abiotic stressors and an update on this gene family in ciliates
Source: Front Genet. 2025 Mar 7;16:1538168. doi: 10.3389/fgene.2025.1538168 (PMC11925944; doi:10.3389/fgene.2025.1538168)
Supplement: Supplementary file 1 [file DataSheet7.pdf]

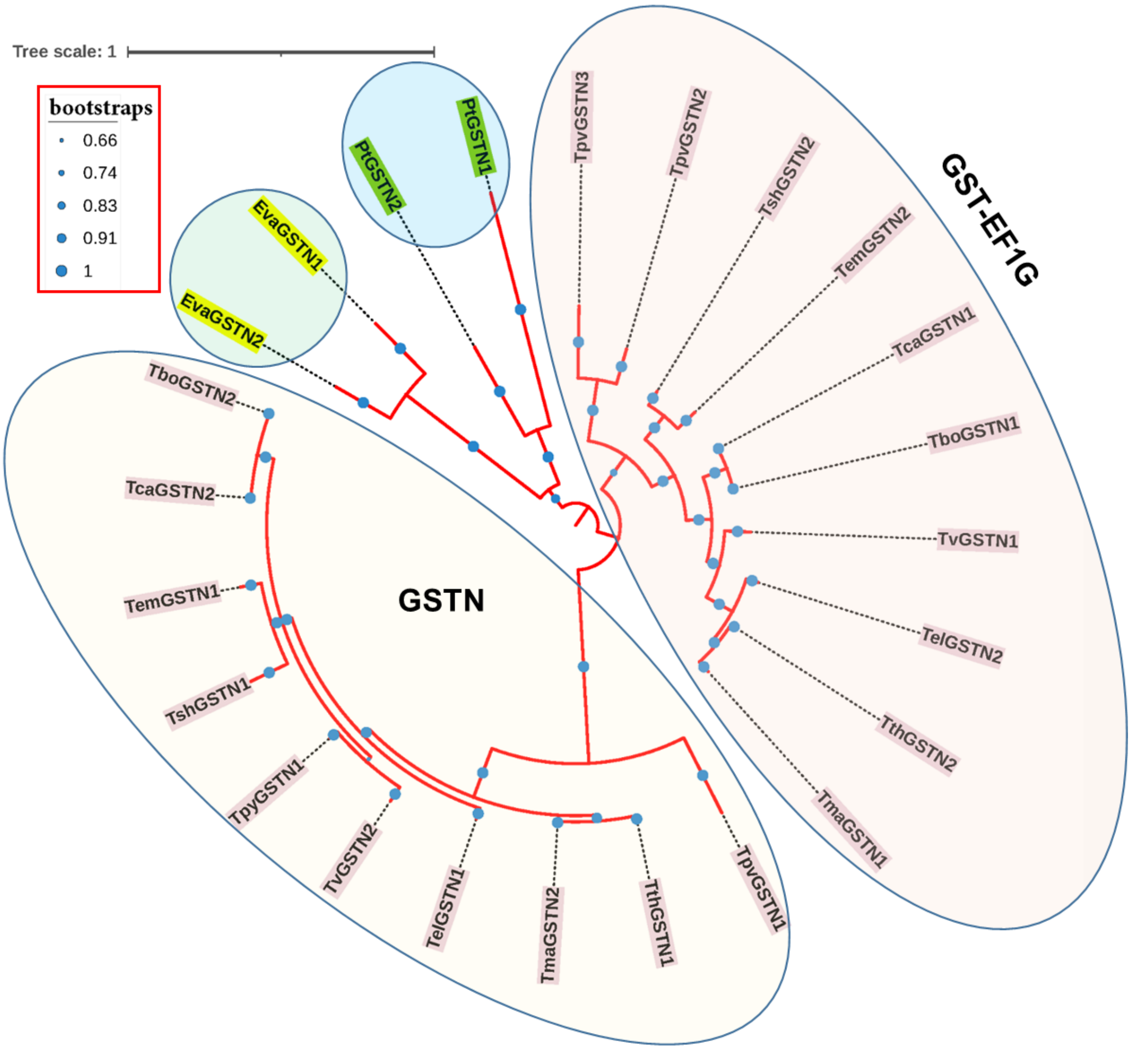

FIGURE S7

Circular phylogram showing the two separate classes GSTN and GST-EF1G from *Tetrahymena* species (see text for further information). Each branch length follows the scale. Calculated bootstrap values, from 2000 replicates, are indicated as spheres of different sizes (values from 0.52 to 1).
