# Supplementary material for: Tetrahymena thermophila glutathione-S-transferase superfamily: an eco-paralogs gene network differentially responding to various environmental abiotic stressors and an update on this gene family in ciliates
Source: Front Genet. 2025 Mar 7;16:1538168. doi: 10.3389/fgene.2025.1538168 (PMC11925944; doi:10.3389/fgene.2025.1538168)
Supplement: Supplementary file 2 [file DataSheet2.pdf]

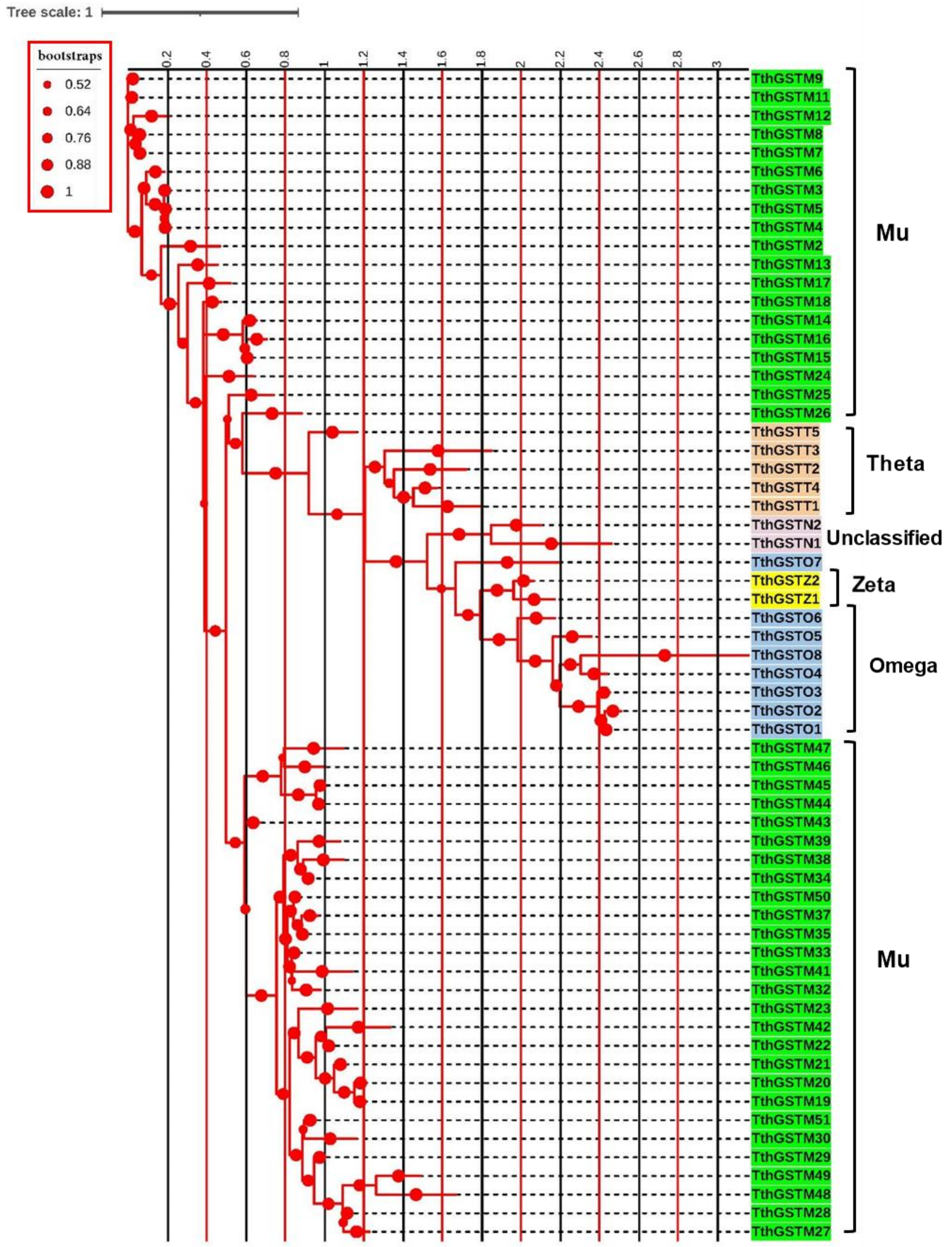

FIGURE S2

Phylogram of the TthGSTs (see Figure 2). Each branch length follows the scale. Calculated bootstrap values, from 2000 replicates, are indicated as spheres of different sizes (values from 0.52 to 1).
