# Supplementary material for: Tetrahymena thermophila glutathione-S-transferase superfamily: an eco-paralogs gene network differentially responding to various environmental abiotic stressors and an update on this gene family in ciliates
Source: Front Genet. 2025 Mar 7;16:1538168. doi: 10.3389/fgene.2025.1538168 (PMC11925944; doi:10.3389/fgene.2025.1538168)
Supplement: Supplementary file 3 [file DataSheet4.pdf]

```
            ....|....| ....|....| ....|....| ....|....| ....|....|
                    10         20         30         40         50
TthMAPEG1   ---------- ---------- ---------- ---------- ---MILIGFL
TthMAPEG2   ---------- ---------M SVVFEVEDHF QLAVLSAVIY SFEMILIGFL
TthMAPEG3   MTFQHFYEDP APTANAPVDN QSVISVDKMY GLVVISALTL CFQCAVYGYF
TboMAPEG1   ---------- --------MA SVIFEVDEHF QLALLSACIY SFQMIMIGFA
TcaMAPEG2   ---------- --------MA SVIFEVDEHF QLALLSACIY SFQMIMVGFA
TelMAPEG2   ---------- ---------M TVVFEVEDHF QLAVLSAVIF SFEMILIAFI
TemMAPEG1   ---------- --------MV TSVFQVDEHF QLALLSATIF SFQMVVVGFL
TmaMAPEG1   ---------- ---------M SVIFEVEDHF QLAVLSAVIY SFEMILIGFV
TpyMAPEG2   ---------- --------MT TTVFEVEEHF QLALLSACIF SFEMIMIGFA
TshMAPEG1   ---------- --------MV TAVFEVDEHF QLALLSAAIY SFQMFLIGFI
TvMAPEG1    ---------- --------MT TTVFEVEEHF QLAVLSAVIF SFEMIFIGFA

TthMAPEG4   MSQKNLQAEK IKEQHVNEDK EVKKSKSKKL GYINQAVATI CTAIVLICFY
TboMAPEG2   MKQQ--QIHQ QTPQPQNDNA -KKP--SKMM GIINKLVAIL GASVAVILFT
TcaMAPEG1   MKQQ--QIHQ QTPQAQNDNA -KKP--SKMM GIINKLVAIL GASVAVILFT
TelMAPEG1   MSNKGQKAEQ IHEQHVIAEK DTKKSKNKKL GIINQVVATL CVGIVLFFFI
TmaMAPEG2   MSQKNLQAEK IKEQHIKENK DIKKSNSKKL GYINQAVATI CTAVILVCFN
TpvMAPEG1   ---------- ---------M DQKKESKKSK ALLIKAVAAL GLVIGVSLSL
TpvMAPEG2   ---------- ------MSSR PEEAKNENKV PFYRVRFFFQ GISIPVGFIM
TpyMAPEG1   MSSQ--KQQQ QKPQQTPEGK -EKTKGSKTI GIINKLVALV GATVAVVFFS
TvMAPEG2    MSST--KTSA TSEKLKTEEK -KQKGGSKTI NIINKIVATL GATAGIFFFN

            ....|....| ....|....| ....|....| ....|....| ....|....|
                    60         70         80         90        100
TthMAPEG1   LPGRIRSKVF NKDFLQAHFG EEHRQSLGTE IDKTFGYPDM GHGRYSDKLS
TthMAPEG2   IPGQMRGKVF TSEFMRENFG KEHLDNTGLP VDNSQGYPDM GHGRYSDKLP
TthMAPEG3   LVGRYRSKIF NKSFLEAEFG QLHKDVTQRS IEAG-GYPDM GSGVYAQRLD
TboMAPEG1   LPGSVRSKVF TKDFMRENFA KEHLEHTGQD VENTMGYPDM GHGRYSDKLP
TcaMAPEG2   LPGSVRSKVF TKDFMRENFA KEHLEHTGQD VENTMGYPDM GHGRYSDKLP
TelMAPEG2   IPGKLRDRVF TPEFMRENFG KEHLENTGLP IDKSSGYPDM GHGRYSDKLP
TemMAPEG1   VPGQMRSKVF TKEFMREHFG KEHQEATGQD VENSMGYPDM GQGRYSDKLP
TmaMAPEG1   IPGKMRGKIF TEEFMRDNFG KEHLENTGLP VDKSQGYPDM GHGRYSDKLP
TpyMAPEG2   IPGQIRGKVF TKDFMRENFG QEHRDNTGTD VESTQGYPDM GHGRYSDKLP
TshMAPEG1   IPGQMRSKVF TKEFMRENFG KEHLAATGQD VENTAGYPDM GQGRYSDKLP
TvMAPEG1    IPGKIRSKIF TKEFMRTHFG KEHQDHTGTD VEKTEGYPDM GHGRYSDKLP

TthMAPEG4   QFCNYQENQV KLYETKLDLI KRFDNESKQY IVLKIGYMLA TFILILTFSV
TboMAPEG2   TILSYQERQV RLYEDRIDLI QKFDNEAKLY AVLKVGFMIA IFMLITTFSV
TcaMAPEG1   TILSYQERQV RLYEDRIDLI QKFDNEAKLY SVLKVGFMIA IFMLITTFSV
TelMAPEG1   QFCDYQESQI KQNEPNLDLI NQFTSESKQY IVLKVGYMLA TFLLIVTFSV
TmaMAPEG2   HLCNYQENQV KLYEEKLDLI KRFDDEGKQY IVLKIGYMLA TYVLILTFSV
TpvMAPEG1   YLFNALGAII PKYEPKMGII NEFQLEGKFY ILTRIGFFLS VFLLQVTMFT
TpvMAPEG2   IAALQQQYLR IQTWMGIENS DLGNFEQKFK ILLKIIFPLA LLVFIVIQMT
TpyMAPEG1   AIYNYQERQV KLYETKQDLI ATFTEEAKLY ALLKIGFMVA IFLIICTLSV
TvMAPEG2    FFFDYQDSCV KLYEEKVDLI NKFDDQAKLY VVLKIGFMVA IYLILCTFSV
```

```
            ....|....| ....|....| ....|....| ....|....| ....|....|
                   110        120        130        140        150
TthMAPEG1   YKDWVYFGKA QRAHYN---- ------FLEA WGPQTLFIII GALKYPLFS-
TthMAPEG2   YAKWLQFAKS QRVHYN---- ------FLEN WGPQTLFIIV GALKFPIFS-
TthMAPEG3   YYSWLLFNKA QRIHSN---- ------FQET LPISVFFLLV AGLQFPITA-
TboMAPEG1   YNSWIQFAKA QRAHYN---- ------FLEN WGPQTLFIIV GALKYPIFS-
TcaMAPEG2   YNSWIQFAKA QRAHYN---- ------FLEN WGPQTLFIIV GALKYPIFS-
TelMAPEG2   YAKWLQFAKS QRVHYN---- ------FLEN WGPQTLFIIV GALKYPVFS-
TemMAPEG1   YNNWLSFAKA QRVHYN---- ------YLES WGPQTLFIII GALKYPVFS-
TmaMAPEG1   YAKWLQFAKS QRVHYN---- ------FLEN WGPQALFIIV GALKYPVFS-
TpyMAPEG2   YNLWIQFAKA QRVHYN---- ------FLES WGPQTLFIIV GALKYPVFS-
TshMAPEG1   YNDWLAFAKS QRVHYN---- ------YIEA WGPQTLFIII GALKYPVFS-
TvMAPEG1    YNDWIQFGKA QRVHYN---- ------FLES WGPQTLFIII GGLKYPIFS-

TthMAPEG4   MVIRIATGRS NPISFQDPKI IITLNRIIQN SLEQGFIFML NYAFFIYFN-
TboMAPEG2   MGIRVISGRS NPIKYQDPAI ILALNRIIQN TLEQSFIFLL NYSFFIYFN-
TcaMAPEG1   MGIRVISGRS NPIKYQDPAI ILALNRIIQN TLEQSFIFLL NYSYFIYFN-
TelMAPEG1   MSIRVITGRS NPISFQDPKI IITLNRIIQN TLEQGFIFMF NFAFFIYYN-
TmaMAPEG2   MLIRITTGRS NPISFQDPKI IITLNRIIQN TLEQGFIFML NYAFFIYFN-
TpvMAPEG1   MLCRVLTGKS NPVQVQDPLI IQVCNRVIQN SVEQILVFAM SFAYYVFWV-
TpvMAPEG2   MRLRVQNRKG NPVAEQDPMI ITLHNRIIQN TIEHSFVFII NLAYLIFVTS
TpyMAPEG1   MVIRIVTGRS NPIKFQDPAI IIALNRIIQN TIEQSFILLL NYAYFVYFA-
TvMAPEG2    MGIRVITGRS NPIKYQDPAI IIALNRIIQN TVEQSFIFLL NYAYFVYFT-

            ....|....| ....|....| ....|....| ....|....| ....|....|
                   160        170        180        190        200
TthMAPEG1   ---------- ---AILGFVA ILGRLLYSVG YMLQAGSSNP IRSIGAVTGD
TthMAPEG2   ---------- ---AVLGFVA ILGRLLYTVG YMLPQGSSNF IRLFGAIAGD
TthMAPEG3   ---------- ---SVFGFIQ VFARLLS-LA YISNQGATHP LRRISSLLIL
TboMAPEG1   ---------- ---AVLGFVA IAGRLSYTIG YMLPQGSSNK LRLMGALVGD
TcaMAPEG2   ---------- ---AVLGFVA IVGRLSYTIG YMLPQGSSNK LRLIGALVGD
TelMAPEG2   ---------- ---AVLGFVA ILGRLLYTVG YMLPQGSSNY VRLFGAITGD
TemMAPEG1   ---------- ---ATLGFVA IFARLCYTVG YMLKQGSSNN IRMIGAILGD
TmaMAPEG1   ---------- ---AVLGFIA ILGRLLYTVG YMLPQGSSNF IRLFGAIAGD
TpyMAPEG2   ---------- ---ATLGFIA IFARLCYTIG YMLPAGSSNI IRVTGAILGD
TshMAPEG1   ---------- ---TVLGFIA TVARLLYTIG YMQKQGSTSL IRILGAITGD
TvMAPEG1    ---------- ---ATLGFVA IFARLCYSIG YMLPAGNFNI LRMLGAIAGD

TthMAPEG4   ----CDQIKA LKSFVLGCLF IKARYIFLFS YVIGAFTGVT SFRAIGFTLN
TboMAPEG2   ----CDQSKI FKAFVLGALF VKARYIFLFT YAIGAAINIT SLRSIAFAIN
TcaMAPEG1   ----CDQSKI FKAFVLGALF VKARYIFLFT YAIGAAINIT SLRSIAFAIN
TelMAPEG1   ----SNQQKA LKSFILGILF IKARYIFLFS YVIGAFTGIT SFRAIGFTIN
TmaMAPEG2   ----CDQLKA FKSFILACLF IKARYIFLFS YVIGAFTGVT SFRAIGFTLN
TpvMAPEG1   ----STN--P VQAYKIGIIF VLSRCIFFVT YIVGIYIQIP SLRSVGFSMN
TpvMAPEG2   ERIEDEQSRM KQILSHGVAF VISRFIFYLS YIIGVQAGLP TLRAFGLLPS
TpyMAPEG1   ----SDQAKF YKGFVLASLF IKARYIFLFT YAVGALTGIT SLRSVAFTLN
TvMAPEG2    ----SDQAKF YKAFVLASLF IKARYIFLVT YSIGAVTGVT SFRSIAFTLN
```

```
             ....|....| ....|....| ...
                     210        220
TthMAPEG1    IVLLISFILS CIACISAYSN ---
TthMAPEG2    IVMLISFILS FIACVQAYYN ---
TthMAPEG3    VSILTTFVLA FIKGVYVMKN HHQ
TboMAPEG1    AVMLISFVLS FIACVNAYTR ---
TcaMAPEG2    VVMLISFVLS FIACVNAYTH ---
TelMAPEG2    IVMLISFVLS FIACVNAYYN ---
TemMAPEG1    IVMLISFVLS FIACVDAYYN ---
TmaMAPEG1    IIMLISFILS FIACVQAYYN ---
TpyMAPEG2    IVMIISFVLS FISCVNAYYS ---
TshMAPEG1    LVMITSFVLT FITCVNAYYH ---
TvMAPEG1     IVMLISFVLS FIACVSAYYN ---

TthMAPEG4    VIIQVAFALE NFGIPVLNIY I--
TboMAPEG2    VIIQVTFLLE NMGISVINVH F--
TcaMAPEG1    VIIQVTFLLE NMGISVINVH Y--
TelMAPEG1    VVIQITFALE NFGVSIINTY I--
TmaMAPEG2    VIIQITFALE NFGIPILNIY I--
TpvMAPEG1    IVVQLVLLIE NLGFPILKSY ---
TpvMAPEG2    HIVQIKLLIH NLGIYSL--- ---
TpyMAPEG1    VIIQITFLLE NLGVPVINVH V--
TvMAPEG2     VIIQIIFLLE NLGTSVINVR I--
```

FIGURE S4

MAPEG GST alignment from *Tetrahymena* species. The sequences in Clans 1 and 2 are separated into two different boxes. Highly conserved blue shaded amino acids are part of the conserved MAPEG family motif. Identical amino acids are shaded in yellow.
