# Supplementary material for: Tetrahymena thermophila glutathione-S-transferase superfamily: an eco-paralogs gene network differentially responding to various environmental abiotic stressors and an update on this gene family in ciliates
Source: Front Genet. 2025 Mar 7;16:1538168. doi: 10.3389/fgene.2025.1538168 (PMC11925944; doi:10.3389/fgene.2025.1538168)
Supplement: Supplementary file 4 [file DataSheet6.pdf]

GST-NTER domain

```
                ....|....| ....|....| ....|....| ....|....| ....|....|
                         10         20         30         40         50
TboGSTN1        ---------M TYQLYAPAGN -FRANMILTV AELTGVKVEL VHTEYSTIKT
TboGSTN2        --------MS GLQILGPLGN -IHINIAQIV AEFVGVPLKH VVVEHKEATG
TcaGSTN1        ---------M TYQLYAPAGN -FRANMILTV AELTGVKVEL VHTEYSTIKT
TcaGSTN2        --------MS GLQILGPLGN -IHINIAQIV AEFVGVPLKH VVVEHKEATG
TelGSTN1        --------MS ELQILGPLGN -IHINIALIV AEFVGVPLKH VVVEHKEATG
TelGSTN2        ---------M TYQLLAPAGN -FRANMLLTI AELAGVKVEH VHTEYASTKT
TemGSTN1        --------MS SLQILGPLGN -IHINIALIV AEFVGVPIKH VVVEHKEATG
TemGSTN2        ---------M SIQLFSPTGS -FRGNMIAVS AELAGVKVEW VHTDMSTIKT
TmaGSTN1        ---------M TYQLLAPAGN -FRANMLLTI AELVGVKLEL VQTEYSETKT
TmaGSTN2        --------MS ELQILGPLGN -ININIALII AELVGVPLKH VVVEHKEATG
TpvGSTN1        ---------M VLQLIGPAGH -IHVNVALIL AELLGVPLDY TKMDFKEMTG
TpvGSTN2        ---------M SVTLHAPHGN -FRANMVRIA GALTGVEVKL HQTEYSETKT
TpvGSTN3        ---------M SIVLYAPHGN -FRANMVRIA GALTGIEVKL HQTEYAESRN
TpyGSTN1        --------MS GLQILGPLGN -IHINIALII AEFAGVPLKH VVVEHKEATG
TshGSTN1        ---MS---QD KIQILGPLGN -IHINIALIV GEFAGVNIEH VVIDHKIATG
TshGSTN2        ---------M SIQLFAPTGH -FRGNMIAVA AELAGVALEW VHTEMSTIKT
TthGSTN1        --------MS ELQILGPLGN -IHINIALII AELAGVPLKH VVVEHKEATG
TthGSTN2        ---------M TYQLLAPAGN -FRANMLLTI AELVGVKLEL VHTEYAATKT
TvGSTN1         ---------M TYQLLAPAGN -FRANYLLTV AELAGVKVEF VHTEYTSIKT
TvGSTN2         --------MS GLQILGPLGN -IHINIALII AEFGGVPLKH VIVEHKEATG

                ....|....| ....|....| ....|....| ....|....| ....|....|
                         60         70         80         90        100
TboGSTN1        PEFLQKNPLG KVPVLVTPEG -PIFESTAII RHLART--SG KLYGESKYE-
TboGSTN2        KEFVKKYPLG IIPILITPEN ETILTPVAII KYIARA--GN LLLGATPLE-
TcaGSTN1        PEFLQKNPLG KVPVLVTPEG -PIFESTAII RHLART--SG KLYGESKYE-
TcaGSTN2        KEFVKKYPLG IIPILITPEN ETILTPVAII KYIARA--GN LLLGATPLE-
TelGSTN1        KEFVKKYPLG LIPILITPDK ETILTPVAII KYIARA--GK QLLGSTPLE-
TelGSTN2        PEFKQKNPLG KVPVLITPEG -PIFESNAIA RHLART--SG KLYGANQHE-
TemGSTN1        KEFVKKYPLG LIPILITPQN ETILTPVAII KYIARS--GK QLLGATPLE-
TemGSTN2        PEFLKKNPLG KVPVIVTPEG -PIFESSAIL RHIARV--SG KLYGASAYE-
TmaGSTN1        AEFKQKNPLG KVPVLVTPEG -PVFESNAIA RHLART--AG KLYGANHHE-
TmaGSTN2        KEFVKKYPLG LIPILITPDR ETILTPVAIF KYIARA--GK QLLGSSPLD-
TpvGSTN1        KQFTKKHPLG IVPVLITEDQ EAIYTPVAIF KYIARA--GK KLLGSNPAE-
TpvGSTN2        AEFKKLNPLG KLPVIVTAEG -PVFETNAIL RHLART--SG KLYGSNNYE-
TpvGSTN3        PEMLAKNPMG YVPIIDVAEG -SLYETNAIL RHIARQ--AK QLYGSTPFE-
TpyGSTN1        KEFVKKYPLG LIPILITPEN ETILTPVAII KYIARA--GN LLLGSTPLE-
TshGSTN1        KEFVKKYPLG LLPILITPQN ESILTPVAII KYIARAG-GK LLLGSTPLE-
TshGSTN2        PEFLKKNPLG KVPVIVTAEG -PIFETSAIL RHIARV--SG KLYGASAYES
TthGSTN1        KEFVKKYPLG LIPILITPDR ETILTPVAIF KYIARA--GK QLLGSSPLD-
TthGSTN2        PEFKQKNPLG KVPVLITPEG -PVYESNAIA RHLART--AG KLYGANQHE-
TvGSTN1         PEFLKKNPLG KVPVLITPEG -PIFESNAIA RFLART--SG KLYGANQYE-
TvGSTN2         KEFVKKYPLG LIPILITPQN ETILTPVAII KYIARA--GN LLLGSTPLE-
```

GST-CTER domain

```
                  ....|....| ....|....| ....|....| ....|....| ....|....|
                         160        170        180        190        200
TboGSTN1          ---------SA LVDQYIDMAV TELLPGLTTT LYAIFGFRPA ERDVLKAAKE
TboGSTN2          ---------ET KIDQFLDIIL GNLHKSYDEI TTSIYGYREY DENSVKNAKK
TcaGSTN1          ---------SA LVDQYIDMAV TELLPGLTTT LYAIFGFRPA ERDVLKAAKE
TcaGSTN2          ---------ET KIDQFLDIIL GNLHKSYDEI TTSIYGYREY DENSVKNAKK
TelGSTN1          ---------ET KIDQFLDIIL GNLHKSYDDI TTSIYGYREY DEISVKNSKK
TelGSTN2          ---------AA LVDQYLDLAT LELLPSLTTT LYAIFGFKPA DKEVLKAAKQ
TemGSTN1          ---------ET KIDQFLDIIL GNLFKAYDDI TTSIYGYREY DENSVKNAKK
TemGSTN2          ---------SS LVDQYLDMTT TELLPALTTI LYSIFGFRPV EREVIKTAKT
TmaGSTN1          ---------AA LVDQYLDMAA NELLPALTTT LYAIFGFKPA DKEILKAAKQ
TmaGSTN2          ---------ET KIDQFLDIIL GNLHKSYDDI TTSIYGYREY DETSVKNAKK
TpvGSTN1          ---------ET KIDQFIDIGL TYLYKSYDDF SAHHFGFREF NETAVKNAKK
TpvGSTN2          ---------SA LVDQYLDMCS CELMPAAMAV LLPIFGYVEY NHETVKTAKE
TpvGSTN3          ---------TS QVDQYLDVVL SEFIPAIFNA VLPTFGYQEY DAATVKKGRE
TpyGSTN1          ---------ET KIDQFLDIIL GNLHKSYDDI TTSIYGYREY DENSIKNSKK
TshGSTN1          ---------ET KIDQFLDIIL GQLCKSYDDI TTSIYGYREY DETSVKNAKK
TshGSTN2          HIQMCIRDSS LVDQYLDMNT TELMPALSTI LYSIFGFRPV DKEVIKAAKT
TthGSTN1          ---------ET KIDQFLDIIL GNLHKSYEDI TTSIYGYREY DETSVKNAKK
TthGSTN2          ---------AA LVDQYLDMAV TELLPSLTTT LYAIFGFKPA DKEVLKAAKQ
TvGSTN1           ---------SG IVDQFLDTAV TELLPSLTTT LFAIFGFRPA DKEILKAAKQ
TvGSTN2           ---------ES KIDQFLDIIL GNLHKSYDDI TTSIYGYREY DENSIKNAKK
```

GST-EF1G domain

```
                  ....|....| ....|....| ....|....| ....|....| ....|....|
                         310        320        330        340        350
TboGSTN1          KA-------- KEAPKKDAPK KDAPKKEA-- PKKKEEVEEE -----EEVPT
TcaGSTN1          KA-------- KEAPKKDAPK KDAPKKEA-- PKKKEEVEEE -----EEVPT
TelGSTN2          KP-------- KEAPK----P KEAPK----- -KKEEKVEEE E---KEETNE
TemGSTN2          KAPPKTEGPK KEAPK----- KDAPKKEA-- PAKEEKVSKE E----EEVPS
TmaGSTN1          KA-------- KEAPK----P KEAAK----- PKKEEKVEEE E---KEEEST
TpvGSTN2          QA-------- TKTEKPAAEK KETKKVEKKE EKKEEKV--E E----EEAPA
TpvGSTN3          KK-------- ---------- AEPKKEEKKP ETKKEEVEAE E----EEAPS
TshGSTN2          KAATKTEAPK KDAPKKDAPK KEAPKKEA-- PKKEEQEEKA E----EEVPA
TthGSTN2          KA-------- KEAPK----P KEAPK----- -KKEEKVEEE E---KEEQPA
TvGSTN1           KA-------- KDAPK----P KEAPKKEA-- PKKEEKVEEE -----EEAPT
```

```
                  ....|....| ....|....| ....|....| ....|....| ....|....|
                         360        370        380        390        400
TboGSTN1          G---PAKWNL YDFKTLYSNA KDKEEAVQDL VKN-FDKENM CIYHLHYQKY
TcaGSTN1          G---PAKWNL YDFKTLYSNA KDKEEAVQDL VKN-FEKENM CIYHLHYQKY
TelGSTN2          G---PAKWNL YDYKTLYVNA KNKEEAIQNL VEN-FDPKTM CVYHLHYQKY
TemGSTN2          G---PAKWNL YDFKTLYTNA KNKEDAITDL VAN-YKEGEY CLYYIHYQKY
TmaGSTN1          G---PAKWNL YDYKTLYVNA KNKEEAIQNL VEN-FDAKTM CIYHLHYQKY
TpvGSTN2          G---EAKWNL YDWKTLYTNA KDKNEAIKNL IEN-FKEGEL CLYHLHYQKY
TpvGSTN3          G---PAKWNL YDWKTLYTNA PNKIDAIKNL QEN-TKEGEI CIYHLHYQKY
TshGSTN2          G---PAKWNL YDFKTLYTNA KNKEEAIQNL VEN-FKEGEY CIYHLHYQKY
TthGSTN2          -----SGWNL YDYKTLYVNA KNKEEAIQNL VEN-FDAKTM CIYHLHYQKY
TvGSTN1           G---PAKWNL YDYKTLYANA KDKEEAIQDL VKN-YDAKEM CIYKLHYQKY
```

```
                  ....|....| ....|....| ....|....| ....|....| ....|....|
                         410        420        430        440        450
TboGSTN1          DGDGKVLYQF NNMKNNFL-Q RCDPA-RKKA FGTYSIYGDE PNLDISGVWL
TcaGSTN1          DGDGKVLYQF NNMKNNFL-Q RCDPA-RKKA FGTYSIYGDE PNLDISGVWL
```

```
TelGSTN2    DGDGKVLYLF NNMKNNFL-Q RCDPA-RKVA FGTYSIYGEE PNLDISGVWL
TemGSTN2    EGEGKIVYQF NNMKNGFI-Q RCDPA-RKRA FGTYSIYGDE PNLELSGVWL
TmaGSTN1    DGDGKVLYLF NNMKNNFL-Q RCDPA-RKVA FGTYSIYGEE PNLEISGVWL
TpvGSTN2    EGEGEVLHIF NNLKNGFI-Q RCEEA-RKKA FGTYSIYGEE PKLDISGVWL
TpvGSTN3    EGEGEVLHIF NNLKNGFI-Q RCEEA-RKKA FGTYSIYGEE PKLDISGVWL
TshGSTN2    EGEA------ ----TGFI-Q RCDPA-RKRA FGTYSIYGDE PNLEISGVWL
TthGSTN2    TGDGQVLYLF NNMKNNFL-Q RCDPA-RKVA FGTYSIYGEE PNLEISGVWL
TvGSTN1     DGDGKVLYQF NNMKNNFV-Q RCDPA-RKKA FGTYSIYGDE PNLEISGVWL

            ....|....| ....|....| ....|....| ....|....| ....|....|
                  460        470        480        490        500
TboGSTN1    FQGAEIPKEM NENPAFEYHD LKKLDISKPE DLQTLREYWS NTKEDESVVG
TcaGSTN1    FQGAEIPKEM NENPAFEYHD LKKLDISKPE DLQTLREYWS NTKEDESVVG
TelGSTN2    FMGATIPPQM NENPSFEYHD LKQLDITKPE DLQILRNYWT NTEEDTSVVD
TemGSTN2    FQGATIPVEM NENPSFEYHD LKKLDISKPE DLNIVRQYWN NTVEDESVAE
TmaGSTN1    FMGATIPPQM NENPSFEYHD LKQLDITKPE DLQILRNYWT NTEEDSSVVD
TpvGSTN2    FIGADIPQEM KNNPQFEYHD LKKLCLNKPE DLETIKAYWC NTTDDESVVN
TpvGSTN3    FIGSDIPQEM KNNPQFEYHD LKKLDLNKPE DFEIVKNYWT QVTDDESVVN
TshGSTN2    FQGATIPVEM NENPSFEYHD LKKLDITKAE DLEVLRQYWS NTVEDESKVE
TthGSTN2    FMGATIPPQM NENPSFEYHD LKQLDITKAE DLQILRNYWT NTEEDTSVVD
TvGSTN1     FLGAEIPKEM NENPAFEYHD LQKLDITKAE DLNLIRDYWT KITEDESVVE
```

FIGURE S6

Unclassified *Tetrahymena* GSTN and GST-EF1G alignments. Shaded in yellow: identical amino acid residues. Inside red boxes: conserved motifs in the GST-NTER and -CTER domains. Shaded in green: cis-Proline-loop (see text). Red line delimits the extension of the EF1G domain.
