# Supplementary material for: Tetrahymena thermophila glutathione-S-transferase superfamily: an eco-paralogs gene network differentially responding to various environmental abiotic stressors and an update on this gene family in ciliates
Source: Front Genet. 2025 Mar 7;16:1538168. doi: 10.3389/fgene.2025.1538168 (PMC11925944; doi:10.3389/fgene.2025.1538168)
Supplement: Supplementary file 5 [file DataSheet9.pdf]

```
           ....|....| ....|....| ....|....| ....|....| ....|....|
                   10         20         30         40         50
TthGSTO1   ----MSQ--L TLYGFLICPY VQRVRFALEN IGVKYDYKEV DLYKFKHKEQ
TthGSTO2   ----MSQ--L TLYGFLICPY VQRARFALEN IGVKYDYKEI DIYKFKHREQ
TthGSTO3   ----MSQ--L TLYGFLICPY VQRVRFALEK IGVKYDYKEI DLFKLKQKEQ
TthGSTO4   ----MSQPHL KLYGNIICPY VQRVRFALEA LKLQYDYVEI DLLAKKHLQE
TthGSTO5   ----MSK--L IFYGYLICPF AQRVRFALEA LKIDYEFVHI DLHAGAQKEE
TthGSTO6   ----MKKP-- ILYGCIVCPY TNKVRFALEM LKIDYKYQEI DILTGKNKNN
TthGSTO7   MDITATNEPV RFYCFVTCPY AIRVRTALEL LQVPYEYNEI DLLT------
TthGSTO8   --MDLVQKKI KIYQSLSCPF CQKVKLAMEL LSVPYFQYEI DIANFEHEQE
                                G-site

           ....|....| ....|....| ....|....| ....|....| ....|....|
                   60         70         80         90        100
TthGSTO1   AYLDINPFGK VPSIS-FNNQ IIYESLPLLE FLENEFG-GV FPQDNIRKTQ
TthGSTO2   AYLDINPFGK VPSIS-FNNQ IIYESLPLLE FLENEFG-GV FPQDNIRKTQ
TthGSTO3   AYLDINPFGK VPTIV-INNQ IVYESLPLLE FLENEFG-SV FPQDNIRKTQ
TthGSTO4   EYLAINPLGC VPTIN-INNS NVYESLVLLE FLEEQFG-NV FPKDTIKRAQ
TthGSTO5   AYLKINPFGK LPSIQ-IDGK VVYESLILLE FLGDHFG-GV FPKDDFAKAE
TthGSTO6   SYLQINPQGR VPSLTTINNK NLYDSQVLLQ YIEDEYK-GL FPQDNYAKGL
TthGSTO7   ---------- ---------- -ISESLVCLE YLNDKYQPGL LPQDSFQRAQ
TthGSTO8   WFKQINPEQT VPALILTDNT PAFESFPFIK KLDSENVNVF SKQNGENYET
```

FIGURE S9.

Partial alignment of Omega class TthGSTs. Shaded in yellow: identical amino acid residues. Inside red boxes: conserved motifs in the GST-NTER domains. Shaded in green: cis-Proline-loop (see text). Shaded in blue: G-site cysteine residues. Gray shading tyrosine residues (Y).
