# Supplementary material for: Tetrahymena thermophila glutathione-S-transferase superfamily: an eco-paralogs gene network differentially responding to various environmental abiotic stressors and an update on this gene family in ciliates
Source: Front Genet. 2025 Mar 7;16:1538168. doi: 10.3389/fgene.2025.1538168 (PMC11925944; doi:10.3389/fgene.2025.1538168)
Supplement: Supplementary file 6 [file Supplementaryfile1.docx]

**Table S1**. Primers and probes used in the qRT-PCR analysis.

| **Target gene** | **Oligonucleotide name** | **Sequence**  **(5’ → 3’)** |
| --- | --- | --- |
| *TthATUB* | TtATUB1 | TGTCGTCCCCAAGGAT |
|  | TtATUB2 | GTTCTCTTGGTCTTGATGGT |
| *TthATUBS* | TtTUBS1 | ATAACTCTCCGTTGCTGAA |
|  | TtTUBS2 | ATCACACTTAGCCATCATGT |
|  | Probe#8 | CTGCCTTC |
| *TthGSTM27* | TtGSTM27A | CTATAGGAGCTGGGATCACT |
|  | TtGSTM27B | GAAAAAGCACCATGATACC |
| *TthGSTM53* | TtGSTM53A | TTGGTATTTACTATGACGATCC |
|  | TtGSTM53B | AGAGCAGCTCTTGCTTAATT |
| *TthGSTM32* | TtGSTM32A | GGGGAATTTAAGCTTACTGA |
|  | TtGSTM32B | CAGCTCTTTTGTATTTCCTG |
|  | Probe#41 | CTTCAGCC |
| *TthGSTM42* | TtGSTM42A | GGAATCTATCCCGAAGAATT |
|  | TtGSTM42B | CAAAACGGGCTTGGA |
| *TthGSTM44* | TtGSTM44A | ATTTTCTCTGAGCCTGAATAGG |
|  | TtGSTM44B | CTATATACTTGATTACAAC |
| *TthGSTM3/4/5* | TtGSTM3A | GCAAACTATTTGATCCAGTTAAC |
|  | TtGSTM3B | TGCCAGTTCATCGCATACAT |
| *TthGSTM13* | TtGSTM13A | CTGTTAAGGATAAAGAC |
|  | TtGSTM13B | TCGATGACATAATTTACAAC |
| *TthGSTM14/15/16* | TtGSTM14A | TATGTCTGTGATGGGTTGAT |
|  | TtGSTM14B | AATGAGTATAGATCAGG |
| *TthGSTM26* | TtGSTM26A | TCATCGCTCAAGTCCTTAC |
|  | TtGSTM26B | ATTTGAGTGAAGACATC |
| *TthGSTM49* | TtGSTM49A | TGAGCTTATGTTCATCGC |
|  | TtGSTM49B | GATAACGAATGATTTGTTGTATC |
| *TthGSTO7* | TtGSTO7A | ATATTACTGCTACTAACGAACCT |
|  | TtGSTO7B | TCATATGGTACTTAGAGGAG |
| *TthGSTO4* | TtGSTO4A | ATGTCATAACCCCACC |
|  | TtGSTO4B | CATAATCGTATTAAAGTTTCAAGGC |
| *TthGSTT3* | TtGSTT3A | GAGTATTAAAAACTTATCTTAGC |
|  | TtGSTT3B | GAGAAAATATCAACAGTCATG |
| *TthGSTT1* | TtGSTT1A | ATGACAACAAAACCAAGCA |
|  | TtGSTT1B | TTTAATTCTAACTTCTACCAAC |
| *TthGSTZ1* | TtGSTZ1A | TTAAAGTATGGAGACGAGGTTA |
|  | TtGSTZ1B | TCAATCTTATTAAGCACCCTCA |
| *TthGSTZ2* | TtGSTZ2A | CAGTGCTATTCATCCCTAT |
|  | TtGSTZ2B | TCATATTGCCTTCTTTTTC |
| *TthGSTN1* | TtGSTN1A | ATTATAGATTTTAGGTCCTTTAG |
|  | TtGSTN1B | CAACTACTACATGCTTCAAA |
| *TthGSTN2* | TtGSTN2A | GCAATTAGATATCACCAAGG |
|  | TtGSTN2B | GGTGTTGGTCCAGTAGTTTC |

**Table S2**. Quantitative RT-PCR standard-curve parameters. Amplification efficiency (E), slope (S), correlation coefficient (R^2^).

| Gene | E (%) | S^(1)^ | R^2^ |
| --- | --- | --- | --- |
| *TthATUB* | 1.94 (94.17) | -3.47 | 1.00 |
| *TthATUBS* | 1.95 (95.29) | -3.44 | 1,00 |
| *TthGSTM27* | 2.11 (110.6) | -3.09 | 1.00 |
| *TthGSTM53* | 2.02 (101.7) | -3.28 | 1.00 |
| *TthGSTM32* | 2.00 (99.66) | -3.33 | 1.00 |
| *TthGSTM42* | 1.96 (95.45) | -3.41 | 0.99 |
| *TthGSTM44* | 1.89 (88.90) | -3.62 | 1.00 |
| *TthGSTM3/4/5* | 1.95 (95.29) | -3.44 | 1.00 |
| *TthGSTM13* | 1.90 (89.91) | -3.59 | 1.00 |
| *TthGSTM14/15/16* | 1.93 (93.07) | -3.50 | 0.94 |
| *TthGSTM26* | 1.99 (98.43) | -3.36 | 1.00 |
| *TthGSTM49* | 1.95 (95.29) | -3.44 | 0.99 |
| *TthGSTO7* | 2.10 (109.6) | -3.11 | 1.00 |
| *TthGSTO4* | 1.89 (89.57) | -3.60 | 1.00 |
| *TthGSTT3* | 1.86 (86.01) | -3.71 | 1.00 |
| *TthGSTT1* | 1.84 (84.48) | -3.76 | 0.98 |
| *TthGSTZ1* | 1.87 (86.95) | -3.68 | 1.00 |
| *TthGSTZ2* | 1.90 (89.91) | -3.59 | 1.00 |
| *TthGSTN1* | 1.88 (87.92) | -3.65 | 1.00 |
| *TthGSTN2* | 1.93 (93.43) | -3.49 | 0.95 |

(1): A slope of - 3.32 indicates 100% amplification efficiency.

**Table S3**. *T. thermophila* cytosolic GST names used in this work and their corresponding gene identifier (according to TGD website).

| **TtGST name** | **Gene identifier** |
| --- | --- |
| **Mu class** | |
| TthGSTM1 | TTHERM_00405440 |
| TthGSTM2 | TTHERM_00211500 |
| TthGSTM3 | TTHERM_00895770 |
| TthGSTM4 | TTHERM_00895760 |
| TthGSTM5 | TTHERM_00895750 |
| TthGSTM6 | TTHERM_00630260 |
| TthGSTM7 | TTHERM_00405500 |
| TthGSTM8 | TTHERM_00211510 |
| TthGSTM9 | TTHERM_00405470 |
| TthGSTM10 | TTHERM_00405400 |
| TthGSTM11 | TTHERM_00405450 |
| TthGSTM12 | TTHERM_00630270 |
| TthGSTM13 | TTHERM_00602860 |
| TthGSTM14 | TTHERM_00490870 |
| TthGSTM15 | TTHERM_00490880 |
| TthGSTM16 | TTHERM_00490900 |
| TthGSTM17 | TTHERM_00490890 |
| TthGSTM18 | TTHERM_00602870 |
| TthGSTM19 | TTHERM_00569200 |
| TthGSTM20 | TTHERM_00569160 |
| TthGSTM21 | TTHERM_00569210 |
| TthGSTM22 | TTHERM_00274620 |
| TthGSTM23 | TTHERM_01289030 |
| TthGSTM24 | TTHERM_00332090 |
| TthGSTM25 | TTHERM_00151420 |
| TthGSTM26 | TTHERM_00794370 |
| TthGSTM27 | TTHERM_00516440 |
| TthGSTM28 | TTHERM_00518469 |
| TthGSTM29 | TTHERM_00516420 |
| TthGSTM30 | TTHERM_00129770 |
| TthGSTM31 | TTHERM_00672290 |
| TthGSTM32 | TTHERM_00661620 |
| TthGSTM33 | TTHERM_00661640 |
| TthGSTM34 | TTHERM_00661650 |
| TthGSTM35 | TTHERM_00169100 |
| TthGSTM36 | TTHERM_00169110 |
| TthGSTM37 | TTHERM_00166100 |
| TthGSTM38 | TTHERM_00661660 |
| TthGSTM39 | TTHERM_00661570 |
| TthGSTM40 | TTHERM_00661580 |
| TthGSTM41 | TTHERM_00661630 |
| TthGSTM42 | TTHERM_00463010 |
| TthGSTM43 | TTHERM_00661538 |
| TthGSTM44 | TTHERM_00077530 |
| TthGSTM45 | TTHERM_00077550 |
| TthGSTM46 | TTHERM_00689990 |
| TthGSTM47 | TTHERM_00077560 |
| TthGSTM48 | TTHERM_00473230 |
| TthGSTM49 | TTHERM_00205200 |
| TthGSTM50 | TTHERM_000169119 |
| TthGSTM51 | TTHERM_000672289 |
| TthGSTM52 | TTHERM_00405480 |
| TthGSTM53 | TTHERM_00518470 |
| TthGSTM54 | TTHERM_000516450 |
| **Omega class** | |
| TthGSTO1 | TTHERM_00572000 |
| TthGSTO2 | TTHERM_00400730 |
| TthGSTO3 | TTHERM_00572010 |
| TthGSTO4 | TTHERM_00572020 |
| TthGSTO5 | TTHERM_00630450 |
| TthGSTO6 | TTHERM_00034950 |
| TthGSTO7 | TTHERM_00924320 |
| TthGSTO8 | TTHERM_00569310 |
| **Theta class** | |
| TthGSTT1 | TTHERM_00499540 |
| TthGSTT2 | TTHERM_000161459 |
| TthGSTT3 | TTHERM_00729240 |
| TthGSTT4 | TTHERM_00499550 |
| TthGSTT5 | TTHERM_00260690 |
| **Zeta class** | |
| TthGSTZ1 | TTHERM_00575360 |
| TthGSTZ2 | TTHERM_00575390 |
| **Unclassified** | |
| TthGSTN1 | TTHERM_00041590 |
| TthGSTN2 | TTHERM_00402120 |

**Table S4**. Several parameters of putative ciliate cytosolic GST genes and proteins.

| Ciliate | GST class | Number of genes with introns (%) | Intron number  /(gen) | Average protein size (aa) | Average molecular mass (KD) |
| --- | --- | --- | --- | --- | --- |
| *T. thermophila* | Mu | 10 (18.5%) | 1(8), 2(1), 6(1) | 248 | 28.98 |
|  | Omega | 7 (87.5%) | 1(7) | 257 | 30.28 |
|  | Theta | 0 | 0 | 230 | 21.67 |
|  | Zeta | 2 (100%) | 2(2) | 219 | 25.20 |
|  | UC | 1 (50%) | 1(1) | 315 | 35.63 |
| *T. borealis* | Mu | 6 (16.6%) | 1(5), 5(1) | 238 | 27.86 |
|  | Omega | 9 (90%) | 1(8), 2(1) | 259 | 30.61 |
|  | Theta | 0 | 0 | 226 | 26.62 |
|  | Zeta | 1 (100%) | 2(1) | 220 | 25.14 |
|  | UC | 1 (50%) | 1(1) | 318 | 36.00 |
| *T. canadensis* | Mu | 6 (17%) | 1(5), 5(1) | 231 | 27.11 |
|  | Omega | 9 (81.8%) | 1(8), 2(1) | 260 | 30.64 |
|  | Theta | 0 | 0 | 224 | 26.55 |
|  | Zeta | 1 (100%) | 1(1) | 238 | 27.52 |
|  | UC | 1 (50%) | 1(1) | 318 | 36.01 |
| *T. elliotti* | Mu | 9 (23%) | 1(6),2(1),4(1),6(1) | 211 | 24.89 |
|  | Omega | 4 (80%) | 1(3), 2(1) | 266 | 31.17 |
|  | Theta | 0 | 0 | 237 | 28.06 |
|  | Zeta | 1 (100%) | 2(1) | 220 | 25.20 |
|  | UC | 1 (50%) | 1(1) | 317 | 35.84 |
| *T. empidokyrea* | Mu | 7 (33%) | 1(6), 5(1) | 225 | 26.29 |
|  | Omega | 5 (62.5%) | 1(4), 2(1) | 260 | 30.81 |
|  | Theta | 0 | 0 | 228 | 26.90 |
|  | Zeta | 1 (100%) | 1(1) | 220 | 25.00 |
|  | UC | 1 (50%) | 1(1) | 321 | 35.95 |
| *T. malaccensis* | Mu | 7 (16%) | 1(5), 2(1), 5(1) | 216 | 25.47 |
|  | Omega | 6 (75%) | 1(4), 2(2) | 250 | 29.46 |
|  | Theta | 0 | 0 | 230 | 27.33 |
|  | Zeta | 1 (100%) | 2(1) | 220 | 25.32 |
|  | UC | 1 (50%) | 1(1) | 317 | 35.78 |
| *T. paravorax* | Mu | 5 (13%) | 1(3), 2(1), 5(1) | 226 | 26.48 |
|  | Omega | 4 (57%) | 1(4) | 262 | 30.98 |
|  | Theta | 0 | 0 | 178 | 21.02 |
|  | Zeta | 1 (100%) | 2(1) | 219 | 25.16 |
|  | UC | 0 | 0 | 342 | 39.12 |
| *T. pyriformis* | Mu | 19 (38%) | 1(15), 2(2), 3(1), 5(1) | 218 | 25.59 |
|  | Omega | 9 (81.8%) | 1(8), 2(1) | 262 | 30.77 |
|  | Theta | 0 | 0 | 231 | 27.24 |
|  | Zeta | 1 (100%) | 2(1) | 220 | 25.08 |
|  | UC | 1 (100%) | 1(1) | 221 | 24.61 |
| *T. shanghaiensis* | Mu | 8 (26.6%) | 1(6), 5(1), 8(1) | 237 | 27.81 |
|  | Omega | 5 (62.5%) | 1(4), 3(1) | 296 | 34.95 |
|  | Theta | 0 | 0 | 229 | 27.00 |
|  | Zeta | 1 (100%) | 2(1) | 220 | 25.15 |
|  | UC | 1 (50%) | 1(1) | 350 | 39.27 |
| *T. vorax* | Mu | 22 (33.8%) | 1(20), 3(1), 5(1) | 215 | 25.30 |
|  | Omega | 12 (80%) | 1(10), 2(2) | 231 | 27.20 |
|  | Theta | 1 (14.2%) | 1(1) | 241 | 28.48 |
|  | Zeta | 1 (100%) | 2(1) | 220 | 25.11 |
|  | UC | 1 (50%) | 1(1) | 318 | 35.93 |
|  | | | | | |
| *Ichthyophthirius multifiliis* | Mu | 0 | 0 | 248 | 29.84 |
| *Paramecium tetraurelia* | Mu | 7 (100%) | 1(5), 2(2) | 226 | 26.53 |
|  | Alpha | 1 (100%) | 1(1) | 710 | 85.72 |
|  | Theta | 9 (100%) | 1(2), 2(2), 3(5) | 387 | 45.06 |
|  | Zeta | 4 (80%) | 1(1), 3(3) | 196 | 22.92 |
|  | UC | 2 (100%) | 2(1), 3(1) | 296 | 34.44 |
| *Blepharisma stoltei* | Mu | 0 | 0 | 219 | 25.28 |
|  | Theta | 0 | 0 | 224 | 26.24 |
|  | Sigma | 0 | 0 | 209 | 24.30 |
| *Euplotes vanus* | Mu | 0 | 0 | 247 | 28.68 |
|  | Omega | 4 (50%) | 1(1), 2(3) | 342 | 39.78 |
|  | Theta | 4 (57%) | 1(2), 2(2) | 233 | 19.16 |
|  | Zeta | 1 (50%) | 1(1) | 188 | 21.37 |
|  | Sigma | 7 (100%) | 1(6), 2(1) | 232 | 26.95 |
|  | Tau | 1 (100%) | 1(1) | 202 | 23.52 |
|  | D-E | 4 (57%) | 1(3), 2(1) | 223 | 26.15 |
|  | UC | 0 | 0 | 330 | 37.44 |
| *Pseudokeronopsis carnea* | Mu | 6 (75%) | 1(5), 2(1) | 199 | 23.10 |
|  | Omega | 0 | 0 | 259 | 30.20 |
|  | Theta | 3 (17.6%) | 1(1), 2(2) | 225 | 26.01 |
|  | Sigma | 4 (28.5%) | 1(4) | 196 | 22.66 |
|  | D-E | 0 | 0 | 197 | 22.17 |
| *Pseudokeronopsis flava* | Mu | 2 (100%) | 1(2) | 223 | 25.83 |
|  | Omega | 2 (66.6%) | 1(2) | 232 | 26.53 |
|  | Theta | 5 (41.6%) | 1(5) | 204 | 23.80 |
|  | Sigma | 7 (58.3%) | 1(5), 2(2) | 188 | 21.76 |
|  | D-E | 0 | 0 | 221 | 25.52 |
| *Stentor coeruleus* | Mu | 0 | 0 | 216 | 24.90 |
|  | Theta | 0 | 0 | 222 | 25.86 |
|  | Sigma | 0 | 0 | 217 | 25.50 |
| *Oxytricha trifallax* | Mu | 1 (50%) | 1(1) | 205 | 24.13 |
|  | Omega | 0 | 0 | 275 | 32.11 |
|  | Theta | 0 | 0 | 240 | 26.68 |
|  | Zeta | 2 (100%) | 2(2) | 190 | 21.85 |
|  | Sigma | 0 | 0 | 239 | 17.93 |
|  | D-E | 0 | 0 | 254 | 29.92 |
| *Stylonychia lemnae* | Mu | 0 | 0 | 221 | 25.40 |
|  | Omega | 0 | 0 | 265 | 30.72 |
|  | Theta | 1 (25%) | 1(1) | 239 | 27.85 |
|  | Sigma | 0 | 0 | 211 | 24.56 |
|  | D-E | 1 (50%) | 1(1) | 318 | 37.06 |

UC: Unclassified, D-E: Delta-Epsilon.

**Table S5**. Domains detected in putative cytosolic GST proteins from *Tetrahymena thermophila*.

| TthGST | Domain | Sequence | Score |
| --- | --- | --- | --- |
| TthGSTM1 | GST-NTER | KELIIGYLENASRGQTVRYILDLVGFPYTEHKYTSTsTEWEKKKFELgLDFPNLPYLIYGDFSISESQNIVNYLIELTNQQY | 16.56 |
| TthGSTM2 | GST-NTER | KELIIGYLENASRGQTVRYILDLVGYPYAEHKYTSSsTEWEKKKSELgLDFPNLPYLIHGDFSISESQNIVNYLIELTNQQY | 17.09 |
| TthGSTM3 | GST-NTER | DNLVLGYWGLPLRGQPLRYILELANYPYTETKYTLSqaTDWFGkDKQELeLDFPNLPYLIHGDFSITESSNIANYLIQLTNQQY | 17.09 |
|  | GST-CTER | EGQDKYRVDNIRYVCDELTAKIFSSTLQ----KEEEKKNQLETQILPKIQQLQKVLG-SQTSFFKKLTLADIYAYTALAYFKKtYSKEYQQFASDFDPFLKRFEEIPRIKNYHQSERYKKL- | 9.49 |
| TthGSTM4 | GST-NTER | DNLVLGYWGLPLRGQPLRYILELANYPYTETKYTLSqaTDWFGkDKQELeLDFPNLPYLIHGDFSITESSNIANYLIQLTNQQY | 17.09 |
|  | GST-CTER | EGQDKYRVNNIRYVCDELTAKIFSSTLQ----KEEEKKNQLDTQILPKIQQLQKVLG-SQTSFFKKLTLADIYAYTALAYFKKtYSMEYQQFASDFDPFLKRFEEIPRIKNYHQSERYKKL- | 8.48 |
| TthGSTM5 | GST-NTER | ANLVLGYWGLPLRGQPLRYILELANYPYTETKYTLSqaTDWFGkDKQELeLDFPNLPYLIHGDFSITESSNIANYLIQLTKQYY | 17.71 |
|  | GST-CTER | EGQDKYRVDNIRYVCDELAAKIFSSTLQ----KEEEKKNQLDTQILPKIKQLQKVLGT-QTSFFKKLTLADIYAYTALAYFKKtYSKEYQQFSSDFDPFLKRFEEIPRIKNYHQS-QRYKKL | 8.88 |
| TthGSTM6 | GST-NTER | EKVVLGYWAFPLRGQPIRYILELANYPYTETKYTPStaTEWFGkDKLELgLDFPNLPYLIHGDFSITESQNIVNYLIQLTNQQS | 18.76 |
|  | GST-CTER | EGELKYKVDNIRYVCDDLISKIFLSTRKN----DEEKKTDLNTQIIPKIKQLQKVLG-NKNSFFNKLTLADIFGYTAINNFKKtYPQEYEEFASTFDPLLKRFEEIPIIKKYHQSdRyFK---- | 9.22 |
| TthGSTM7 | GST-NTER | QTINLGYWGFPLRAQPIRYIFELAQYPYQQTNYTFEgaKDWFEkDKKDLgLDFPNLPYLIHGDFKITESQNIINYALDVTKQHH | 17.63 |
|  | GST-CTER | YGLTKFKIDNVRFLCDELIAKTFLASTK----TGEEKKAEINSSVTPKFKSLQAVLGNKKFFFEDKLTLADIYAYTAINALKHlLNDEYKQFAQTFDPFMKNFEEVPLIKAYHSSNRYPKFA | 9.74 |
| TthGSTM8 | GST-NTER | QTINLGYWGFPLRAQPIRYIFELAQYPYQQTNYTFEgaKDWFEkDKKELgLDFPNLPYLIHGDFKITESQNIISYALDITKQHH | 17.00 |
|  | GST-CTER | FGLIRFKIDNIRFLCDELIAKTFLASTK----TGEEKKAEINNSVIPKFKSLQAVLGNKKFFFEDKLTLADIYAYTAINAFQHlLNDEYKQFAQAFGPFMKNFEEVPLIKAYHSSNRYPKFT | 9.31 |
| TthGSTM9 | GST-NTER | LTLNLGFWALPLRAQPIRYIFELAQYPYQQTNYTFKesKEWFEkDKKELgLDFPNLPYLIHGDFKITETQNIINYALDITKQQN | 15.74 |
|  | GST-CTER | YGLTRYKVDNVRFLCDELIAKIFLASTK----TGEEKKAEINNSVNPKLKYLQGALGNKQFFFEDKLTLADIYVYTAVNGLQHlLNDEYKQFVQTFGPFMKNFEEIPLIKAYHSSNRYPKLS | 10.09 |
| TthGSTM10 | GST-NTER | QTINLGFWALPLRAQPIRYIFELAQYPYQQTNYTLEesKEWFEkDKKELgLDFPNLPYLIHGDFKITESQNIVNYALDATKQHH | 17.14 |
|  | GST-CTER | HLLGSGLIRFKIENVRFLCDELTAKTFQASRKTGEEKNTEINNTVIPKFKFLSVVLGNKKFFFEDKLTLADIFAYTAIKTLQHvLNDEYKQFAQVFDPFMKNFEEIPQIKAYHSS-DRYPKF | 9.78 |
| TthGSTM11 | GST-NTER | LTLNLGYWAYPLRGQPIRYLLELAQYPYQQTNYTFEgaNDWFEkDKKELgLDFPNLPYLIHGDFKITESQNIVDYVLDLTKQHH | 18.14 |
|  | GST-CTER | SGLTKFQIDNVRFLCNELISKIFLASTK----TGEEKKAEINNSVTPKFKRLQAVLGNKKFFFEDKLTLADIYAYTAINALQHlLNDEYKQFAQAFGPFMKNFEEIPLIKAYHSSTrypRFK--- | 10.42 |
| TthGSTM12 | GST-NTER | NTITLGYWALPLRGQPIRYIFELAKFPYQQTLYTSAtaSNWFGkDKQELgLDFPNLPYLIKGDFKITESQNIVNYAIDITKQQH | 15.25 |
|  | GST-CTER | TGQKTFKIDNIRFFCDELTSKVFLATRKS----GEEKTTEINNVLIPKFKYLHKQLGNNVYFFDNKLSLADIFAYTAINVFKLkFNEEYQQFSSTFDPFMKNFEEIPLIKEYHNSdRYPKMP- | 11.09 |
| TthGSTM13 | GST-NTER | EKIVLGYWAVHLQGQPARYVLELAGIPYEDRLYTMQnrADWFEkDKQTLgFDYPNLPYIIHGDFKITESQNVVNYVIEVTNQQK | 19.46 |
|  | GST-CTER | ITESQNVVNYVIEVTNQQKLLGegkdkyrVGHVRYVcQEILGKLFGAIMKENAEeKQNAIQNDVLPKarlvQTYLGSQNkfcseLTIADIYAYVFFFNLKKkAPEAYAEFAAQIDPLLQNFESIPNIKKYQESerfaKiNN---- | 6.86 |
| TthGSTM14 | GST-NTER | KQLLLGYWNIPLRAQPIRYILELAQYPYSEKKYSQKeaQEWFGnDKQNLgLEFPNLPYIFHGDYHLTEASNIANYVLEITCQQY | 13.72 |
|  | GST-CTER | YVLEITCQQYLLGCGDDKFRIGNIRYVC-----DGLIIDVFKtlKMTPeEKQKLLENNIIpDlySlKEALGEKtyffgrLTVADIYAYCAFVNFKLfFPNEYKQFASSFNNLIINFEAIPEIQAYQKSdRFPKFQL | 6.83 |
| TthGSTM15 | GST-NTER | KQLLLGYWDIPLRAQPIRYLLELGHFPYTEKRYAQKdaQEWFGkDKQNLgLEFPNLPYITHGDYHLTEAANIANYVIEITCQQN | 14.07 |
|  | GST-CTER | CGDDKYRIGNIRYVCDGLIIDVFKTLKM----TAEEKQKLLENNIIPDLYSLNEALGEKTYFFGR-LTIADVYAYCAFVNFKLfFPNEYKQFASSFDHLIINFEAIPEIQAYQKSdRFPKFQL | 7.00 |
| TthGSTM16 | GST-NTER | KQVTLGYWNIPLRAQPIRYLLELGHYPYVEKKYTQQdaSEWFGkDKQKLgLEYPNLPYLIQGDFHITEASNIANYVIEITHQQR | 15.78 |
|  | GST-CTER | YVIEITHQQRLLGCGDNKFRVGniryvcdglIIDVFK----TLKMTPEEKQKLLEnNILPDLYSLKEalGeKTYFFGN-ITIADVYAYCALVNFKLffPNEYQKFANSSFDRIIHNFEAIPEIKAYQKSdRFPKFQF | 7.82 |
| TthGSTM17 | GST-NTER | DTLILGYWAQPVRAQPIRYILEIGKYPYKENQYKTPAEWFEkDSMSLgLQFPNLPYIIKGDLKITESHNVAQYAIEVSNQCN | 15.05 |
|  | GST-CTER | TGQQKYKIENVRLVADEVMMKVFGAIKLS----GEAQTNEFKTNIIPKLTLLQKHLGNKVYFFDNKLSVADIYTYSGLYILKNkFPEQYQPFAATFDPFIKNFESIPQIKEYQSSdRFPKLR- | 10.11 |
| TthGSTM18 | GST-NTER | SELILGYWGLPLKGQPIRYLLELGKQAYQDKKYSNKDEWFQqDKLNLgLDFPNLPYIIHGDVKMTESQNIVAYIIDLTKQTN | 16.92 |
|  | GST-CTER | ADKTKFKIQNIVLFCNDLIGS-FKPISM----KQGEERTQEIEKILPKIKLLEKDLNKKAHFFNNSLTLADIYAYCIIDNFQQlGGDSYKPFEESFKAFLDAFRSIPEIKAYIES-DRCTRF | 11.80 |
| TthGSTM19 | GST-NTER | DKIVLGYWAGPGKAQPSRYLLEISGVKYEEVRYTNPADWFGkDKYALgLSFPNLPYLLDGDVKITESETIFDYLIHRLNKTE | 17.69 |
| TthGSTM20 | GST-NTER | YKIVLGYWAGPGKAQPARYLLEISGVKYQDVRYSKPADWFGkDKYALgLPFPNLPYLIDGDVKITESETIFDYLIHKLNKTQ | 19.72 |
|  | GST-CTER | YLIHKLNKTQLLGQDNDKYTVDtLRNLIGDigtrlQMLTQKEGDDKTKflnEQVLPKIKDIHKFLGHKEYLLGY-FTAADLYFLSFARNLKKfYPE  TYQEFALTFDGLVTRLEAIPQIAAYISEKRHP--- | 6.81 |
| TthGSTM21 | GST-NTER | DKIILGYWNTPGKAQPSRYLLELSGVKYEEVRYSYPaAEWFGrDKYALgLPFPNLPYLLDGEVKITESETIFDYLIQRLNKVE | 18.49 |
|  | GST-CTER | ELLGQGNDKYIVDNLKNLFSDIgTRMYIYS---QKEGEEQTKFLsEQILPKIRDIHKFLGQKEYLLGY-FTAADLYFLCFAKGFKNtLPDSYNEFAATFNPLIQRLENIPQIAKYISEgRHP---- | 6.86 |
| TthGSTM22 | GST-NTER | QKITIGYWKGPGKVQPSRYLLEISGVEYQETLYTDPAQWFGkDKYSLdLPFPNLPYLIDGDVKLTESETIFDYLVNKLNMSE | 19.43 |
|  | GST-CTER | KESDRFIVDNLRNIFSDLCTS-LFQVFQK---QGEEKLKIFNCQILPKANDIHKFLKNKEYLLGY-FTVADLYFLSFARNFKSfMPEQYEEFAGTFDALKEKIESIPQIASYISEgRHPIL-- | 6.72 |
| TthGSTM23 | GST-NTER | SKIIIGQWASAGKLVPIKLLLELAGAQYEVVNYSKPDEWYAkDKLILgLPFPNLPYLIEGDIKLTETETIFDYLVYKLKKND | 17.12 |
|  | GST-CTER | QEKDKFIVGNIRNLFSDLYAK-FSQFIQ----KQGDEKQQILnEQIIPKIKDIHKYLNKKDYLLGY-FTVADLYLFPAALLLKQnLPQVYSEFASSFDPFLIRIQQIPQIAAYLSDNSKV--- | 8.26 |
| TthGSTM24 | GST-NTER | NKLTLGYWEGYGKAQPARYLLELTKTPYNNVQYVEDDKWFKqDKLNLgLDFPNLPYVIDGEFKLTEFVAIFDYLSEKYGKPE | 20.07 |
|  | GST-CTER | YLSEKYGKPEWLGKGNDKYVVSslrslfdeLASLNYHgsLKKTEEEKKQHILDNIVPKYRYLSKFIGKKEYLLGY-FTVADLYFLSVINAFKAtlPQDSYAEFAPVFDPIIHRIASIPEIAAYIASdRHPKQ-- | 8.31 |
| TthGSTM25 | GST-NTER | MILGYWEIPGKCQAIRFLLEILKVEYTEKRYTFKnsREWFEeDKLKIgLDFPNLPYFIDGNIKLSESNSIVTYILDKYSKQE | 20.31 |
|  | GST-CTER | TEKRYTFKNSREWFEEDKLKIGldFPNLPYFidgniklsesnsivtYILDKYSKQEWKGknEDIYkvDELRFLFTDLQslgylsskgDqdafkNylkkldfvyQFIKGESkfllgyFTIADLYSWECITRLKVqWPEYYQKYSDKFDQLIQNIENIPELYSYIQSdrclKiQPLKK- | 6.83 |
| TthGSTM26 | GST-NTER | TDLVLGYWPFAGRNMPVIFMLEILNIPYQVNIFDQNTWFGKEKEDLnLDFPNLPFLIDNSngMKITEIHNIVNYILYKYEKGR | 16.68 |
|  | GST-CTER | NPIDDFKIDEIRFILNDVFTQIsSATAKFPQENQIKYAQDTIVPKLNKLIKFIEQKNQaTtSytQYFVMGYLSVVDCYFYIISKYFQKYFPSLYDDYSHVFDLVlNSFENIPEIKKYINSdKYPTLG- | 9.41 |
| TthGSTM27 | GST-NTER | MIILGYWNLRGYAQPIRLLLEYLQVDYKEKLYNQDgEEWLNvDKQQLkTNFPNLPYIIDGDIVVTESKVIPIYLAKKFKNYE | 23.04 |
|  | GST-CTER | NEITFLQILEILKELRDSLLNSAKVPSF-----KEEKDQIFNEKFNITFEKIKKQLGENKYLLGN-LSFIDFYFYEVLK---- FFQFFYPKLSIFTDYIDRIENIPQIKNYLETKenkifildRMKSYF | 8.82 |
| TthGSTM28 | GST-NTER | MIVLGYWNLRGYAQPIRLLLEYLQVEYKDKLYHENgEEWFNtDKQELkTNFPNLPYLIDGDVVVTESIVIPIYLAKKFKKYE | 24.06 |
|  | GST-CTER | FNQNEIIFLEILSILKDLRDTLnsSARVPS----FKQEKDKIFNEKFNVTFEKIKKQLGENKFLLGN-LSYIDFYFYEVLK----IFKFFYPNLSIFTDYIDRIENIPQIKNYLETKenkiflldRMKSLY | 7.59 |
| TthGSTM29 | GST-NTER | MIILGYWNLRGYGQSIRLLLEYLQVEYQDKLYHENgEEWFGtDKKNLnTNFPNLPYVIDGDVVVTESKVIPIYIIKKFKRFD | 20.89 |
|  | GST-CTER | NEITYLQLQEILKELLDKLEAQARIPSF-----KEEKEKIFNEQFNITFEKIKKQLGNQEYLLGN-LTYVDLYFYEVLRNFQ----LFYPKIQIFTEYLKRIENIPQIKEYIQTKENKTFI | 8.90 |
| TthGSTM30 | GST-NTER | MIILGYWTHRGFAQPIRLLLEYLEVGYQEKLYAEGgDEWYNkDKRELkSNFPNLPYLLDGDNVITESKVIPIYLIKKFKRFD | 21.08 |
|  | GST-CTER | FNLDEITVLQLIEVAKDLIDQLntQARVPS----FKEEKMKIFNEKFQITFEKFKKQLDGRDFLVGK-FTYADLYFYNILKYFH----FFYPEISIFTEYIQRIENIPQIKKYLQTKdnqvylleRLKKLF | 9.93 |
| TthGSTM32 | GST-NTER | MITLGYWNVRGLGQSIRFLLAYLGVEYNSKVYSTAEEWFGkDKNNLgLEFPNIPYIIDGEFKLTESSAIPIYLLRKYKRAD | 23.81 |
|  | GST-CTER | YSEREVRVAQLIGVIKDIYKE-TIPVCFS--PDFDKIKDQAFAKGEVLLKKLVSFLGD-KEFLLSTLTYADFLLYEVLCYY--KYIYPQAITPTLTAYMNRFENLPGIKQYIA-NpsINLKAF | 10.58 |
| TthGSTM33 | GST-NTER | MITLGYWNARGLGQSIRFLLAYLGVEYTNKAYSTPEEWFGkDKNNLgLEFPNLPYIIDGEFKLTESQAIPIYLLKKYKRVD | 23.30 |
|  | GST-CTER | NSEREVRVAQLTGVIKDIYKE-TLPVCFS--PDFDKIKDQAFAKGEVLLKKLVSFLGD-KEFLLTTLTYADFLLYEILCYY--KYIYPQAITPTLTAYMNRFENLPGIKQYIANpninlKAFLPPF | 11.13 |
| TthGSTM34 | GST-NTER | QMTTLGYWGIRGLAQPIRFLLAYLGVQYTNKAYANPEEWFGkDKNELgFDFPNIPYLIDGDLKLTESSAIPIYLIRKHKRNE | 23.43 |
|  | GST-CTER | YSEKEVRVAQIVGVIRDLFKE-LTGLCFN--PDFKNIKEKLYtEKLELLIKRLGAYLGDKEFIVGT-LTYADFLFYEALSYI--RHIYPQAICATLTAYINRFENLPGIKEYIAShAQELKVF | 10.75 |
| TthGSTM35 | GST-NTER | MITFGYWNIRGFGQPIRFLLAYLGVKYTNKTYASLEEWFGkDKDNLgLEFPNLPYLIDGDVKLTESFAIPVYLIKKYKKFQ | 22.58 |
|  | GST-CTER | STNKEVKVTQLMGVIRDIAKEINLTCFRP--DFYEVKEKVYTEKVEYMFKKLTNFLG-NKLFLLESLTYADFHFYELVNHVQYIYPQAMTG--ALTAYLKRFENLPGIKEYIANpninlKAHLPGF | 9.55 |
| TthGSTM37 | GST-NTER | MITFGYWNIRGFGQPIRFLLAYLGVKYTNKTYASLEEWFGnDKDNLgLEFPNLPYLIDGDVKLIESFAIPVYLIKKYKQFQ | 21.93 |
|  | GST-CTER | YLIKKYKQFQLLGLQADGSSTNkevkvtqLMGVIRDiakeinltcFRPDFYEVKEKVYtEKVEYMFKKLTNFLG-NKLFLLERLTYADFHFYELVNRVQYIYPQAMTA--ALTAYLKRFENLPGIKEYIANpninlKAHLPGF | 9.74 |
| TthGSTM38 | GST-NTER | MATLGYWGFRGLAQPIRFLLAYLGVQYTDKHYTKGEDWFEnDKKNLgMDFPNIPYFIDNDIKISESSAIPFYIIKKYKKPE | 23.37 |
|  | GST-CTER | FNEKEIKVQQVIGVIKDINKE-LMGLCFN--PDFANAKDKVYnEKLSVGLKKLNDFLGNKDFLLGT-LTYADFLFYETLSYF--KHIFPQSITPTLANYLNRFQNLPGVKEYIA--KPSVDL | 11.37 |
| TthGSTM39 | GST-NTER | MQGTFGYWWVRGSAQPIRFLLAYLKIEHNSKIYTNFDDWFAiDKQNLgLDLPNIPFWIDKDVKLTESAAIPVYLIKKCNRHD | 19.45 |
|  | GST-CTER | FNYKEVQVQQLLGYFRDVNRE-FTSNIC-----PNKDFENIKDKLLneKMQNMLDKIVKirGNqQYLLGE-ITYADFVFYEILNYYKYIYPQTINK--SLQDYINNFESLPGIKEYLLLpdidlKqFLPSQF | 8.75 |
| TthGSTM41 | GST-NTER | MITLGYWGVRGLGESVRYLLAYLNVDYKHQAYYNPQDWFAkDKAQLkIEFSNLPYLIDGEQKITDSYAISIYIIRKYHRED | 22.28 |
|  | GST-CTER | YNEREVKIAQLIGVTRDIFQQ-IVHICFS--PQFDKIKDQAFEKGQIFLNQLTDFLGDKQFLLGH-LSLADFLFYEALTYY--QVLHSQSITQRHEEYMHQFESLPGIKQYLNYaSKNFNSF | 8.05 |
| TthGSTM42 | GST-NTER | NQITLGYWNIRGLGQLSRYLLEYTGLKYKEKRYQKLEEWFQkDKQGLgIEFANLPYLIDGDLKLTESHAVNLYIIRKSGKNE | 22.85 |
|  | GST-CTER | NLIEESKIRELIGYLEDFFRQ-ILTLCFN-PQFSIIKQQKYNDDFSLRLQRLENQLTkDNrKWLNGQSLSLPDFIFYEISQYIKGIYPEEFKKLPKIQAFQARFEEIEQIQDYMMSEeYIYAPF | 16.49 |
| TthGSTM43 | GST-NTER | DKLILGYWPFRGVAQTIRYLLEYLEVPYEQKSYMTYEEWFGkDKKELgADFPNLPYIKQGDFILTESYAIIIYLCKKYNRLD | 22.22 |
|  | GST-CTER | DLIRETKVHQVASAIKDTGKT-IGDLCFN--PKFHSVKDEVYtQKLSQMLKKTTDFLGDKQFLLGE-LTITDFMFYEQLQYF--KNIFPESITPQLQQFIERIENIPKIKEFLNSdrifKkqFLPPTF | 11.96 |
| TthGSTM44 | GST-NTER | SKLIFGYWNGRGRGQQIRFLLEYVEADYEEKTYIFSepeqDEWFKkDKKALKPFPNLPYIIDGDFYLSEHDVVIKYIVKKHPKYH | 21.05 |
|  | GST-CTER | GPNDEFIVDQLVSVINDIRAT-IKDLCF-----NPKVQEVKKEVLATTHTKFNQLIEfKKtNTFLLPYLTIADFKLIEVLLYYKAlDADQFDANLNVFNPYIYHFHSLPRISEYLKTdRyKSNTVF | 9.12 |
| TthGSTM45 | GST-NTER | SKLVYGYWNTRGRGQQIRFLLEYVEASYEEKIYHFNnpDEWFQkDKKTLKPFPNLPYIIDGDFYLSEHDSVIKYIVKKHPKYQ | 21.24 |
|  | GST-CTER | GPNDEFVIDQLISVINDLRQT-IRDFYF-----NPKILEIRKDVLPNTHPKFNQLIEfkGKNTFLLPYLTIADFKLVEVLLYYKClDEELFNTHLSVFEPYIQSFYSLPRISEYVKTdTyRSYSEF | 9.63 |
| TthGSTM46 | GST-NTER | DKIILGYWDYRGRPQPLKFLLEYMGIPFEQKYYSYDnpDEWYEkDKKTIKPFSNLPYLQTKDGILTETCSIIKYLLKRFPQFK | 20.10 |
|  | GST-CTER | KEQDEIFVDQMLSVMNDLRDV-LKALHF-----NPDVHKVKRDTLLTILPKMLDFKDirGDKQFLLPYLTIADFELVEILLLFKHlDSELFDQHLSSFEKFLQDFHNLPRIKEYVRSdKyQKLLTF | 8.18 |
| TthGSTM47 | GST-NTER | SQLLFGSWIQRGNAMPIRFLLEYTQTNYNEKIYYSEneSEWFQkDQKQFKQFANLPYIIDGDLKLTDVQTIMRYIAKRNSNYI | 16.50 |
|  | GST-CTER | HGNDEDRINQINLVISEIGLY-VKEQFF-----NPKVHEEKRDFLNRCQKKFGSFVNirGKNKFIFPYVTTADFNLVELLLHYKClDNELFEKNLPSLNEYIQHFYNLPAIKDYTITEryQMHTEF | 7.92 |
| TthGSTM48 | GST-NTER | SKIKLGYWDVRGRGEPIRLLLNYLKLEYEDEIYPLSdrEKWFNFKRNSqELFINLPYVQIESketqsqSIYVESDSISIFICQNFGGEQ | 18.03 |
|  | GST-CTER | GEQLLGKDLVLLSKMRGVTEDVklyLSRYAY--HNDFAEADQLFLqKRLQPILLRLNTYLQNYKYLLGSNISYLDFVLYESLKTLEKIKKEYLQSHTYLVQYIKNIENIPQIKAYLSSERFHKDF | 13.72 |
| TthGSTM49 | GST-NTER | SNLILSYWNLRGRTEPIRMLLNYLQLPYTYKGYDLSsyNQWKQvDKPALqSDFPNLPYLKDGDYVLTESDAIAQYVCVKANRED | 23.86 |
|  | GST-CTER | TIEDRINIARIRGIVNENIYL-IGQLAY-----SPKYKEELKANFQrfsVPFGQLNNYLNNKEYINNGKICYYDFYLYELMFIAHKifKEENVFDVFPNLRSHYYRIQNLPQIQDYLNSDRYNKSF | 14.25 |
| TthGSTM50 | GST-NTER | MITFGYWNIRGFGQPIRFLLAYLGVKYTNKTYASLEEWFGkDKDNLgLEFPNLPYLIDGDVKLTESFAIPVYLIKKYKQFQ | 22.59 |
|  | GST-CTER | YLIKKYKQFQLLGLQADGSSTNkevkvtqLMGVIRDiakeinltcFRPDFYEVKEKVYtEKVEYMFKKLTNFLG-NKLFLLESLTYADFHFYELVNHVQYIYPQAMTA--ALTAYLKRFENLPGIKEYIANpninlKAHLPGF | 9.84 |
| TthGSTM51 | GST-NTER | MFILGYWESRGIAQAIRLLLEYLEVEYTEKQYYETEQWFEkDKKELnTNFPNLPYLIDGEVVVTESIVIPIYLIKKLKRYE | 19.43 |
|  | GST-CTER | FNQNEIIFLQLLTIMKDLRYDIYISTKVP--SFREEKHKLYNEKYFVTLQKLIRQLD-KKQYLLNNFTYADIMFYDLLRYIE----FIYPEKSILPEYKERIENIPQIKKYLDTKenkifilERMKDL | 8.15 |
| TthGSTT1 | GST-NTER | PSTKLYIEWLSQPSRAIVTFCLIENIPHELVEVRIKklenrtPEYKKMFPTAKLPGMSETLENGEqFNLFESHAIMRYLADRYNKSN | 15.36 |
|  | GST-CTER | NIQLKAKVDSYLDWHHTNTRKCapylFDQYFAPvLGIKPQFDVNTLFKEVESVFRFIERVWLDqgkNKYIGNNQqLTIADLSCYSEIIQMKFDDYDFKNKTPILYEWMQRIEALPEIQKTHQVlfKlapqisknKQKAKL | 16.48 |
| TthGSTT2 | GST-NTER | QTLSLYMDWMSQPSRCVAIFCMINKIPVDISEVKILkGQLRSqEYKRINPNMRVPTIKDGKFVLYESHAILKYLIASRAQYI | 15.08 |
|  | GST-CTER | DIKERALVDQYLDWHHTNIRNAgmyIFNYFVLpsLGIQSKQNKETIYKLFIQSLKMIDTIFLaDKPYIASKEkATIADLSCYCEITQVN-LIDFDFSPYPNILKWMQRLtAEYPQLVEGHQPfmKfvQKIKEK | 15.56 |
| TthGSTT3 | GST-NTER | LNYEIFMDWGSQPSRAVMTVVYFLKIPHKINEVRILkkQNVSEQYKKINPDQKIPCIIDNEnFFLNESHSIMRYFCQLYGDNQ | 14.38 |
|  | GST-CTER | NLKKRALIDSYLDWHHSNTRK-MHRMLF-----KKLFEPQLGiqtsiniEELEsdvqKALAFIENNYLNHknkFFFGFDNYTLADISAYCELYQSK-VVNYSFQPYPNILEWMSKMQQINEIKQTHKVydellnKRIQKQS | 16.13 |
| TthGSTT4 | GST-NTER | --MKIFIDWISQPSRAVVTYCLIENIPHEIIQVRV-NALEHRKPEYiqiNPSAKVPAISDRLengeiFNLFESHAIMRYLADRYNKFK | 16.73 |
|  | GST-CTER | NIQLRALINSYLDWHHNNTRKCgayLFDLYSSgvLGIKPKNNIETLYKEIEIILKFIDQIWLqeGKNKFIGNNiqLTIADISCYCEVSQMIIDSYDFKNKTPNLYNWMKRIEQIPEIQQTHQIlfKlAPKMSQ | 16.12 |
| TthGSTT5 | GST-NTER | NKLKLFYNSFSQPSRAVKCLLKIGKVDYEEKFVNL-AKGDQFKPEVkslNWNCQVPFIEDNGFVVFESHTIMRYIHQRFNLDN | 20.70 |
|  | GST-CTER | KLEDKTKIDMYLDWHHSNTRRSanYVTASLLskRTGQPSLYVEDLVhKELLKAVQNLNDNLLNNpsHYIFGFQkPTIADISCYQELTELK-LINFDFKKYPNLDAFMNQMSNIPEIKEVDKDfSDIALKI | 15.03 |
| TthGSTO1 | GST-NTER | SQLTLYGFLLCPYVQRVRFALENIGVKYDYKEVDLYkFKHKEqAYLDINPFGKVPSISFNNQIIYESLPLLEFLENEFGGVF | 18.51 |
|  | GST-CTER | DNIRKTQQRIWANYFDQNFID-KMWAILGiikkkDAEGSKKLANELAEAVRffTKNSKLSERIKlNpNNYFEGDTLTYVDFAVVPHFKMLDAmyraffkvnffdQIENQDELIQNFKTYYQNVISSDSFKRTVT-NPLNLPA | 10.68 |
| TthGSTO2 | GST-NTER | SQLTLYGFLLCPYVQRARFALENIGVKYDYKEIDIYkFKHREqAYLDINPFGKVPSISFNNQIIYESLPLLEFLENEFGGVF | 17.82 |
|  | GST-CTER | DNIRKTQQRIWANYFNSNFID-RMWAIFGiikkkDAEGSKKLANELAEVLRffTKNSKLSERIKlNpNSYFEGETLTYVDFAIGPHFKMLDAmyrvffkvnffdQIENQDELIQNFKIYFQNVVSSDSFKRTVT-NPLNLPA | 9.82 |
| TthGSTO3 | GST-NTER | SQLTLYGFLLCPYVQRVRFALEKIGVKYDYKEIDLFklKQKEQAYLDINPFGKVPTIVINNQIVYESLPLLEFLENEFGSVF | 20.29 |
| TthGSTO4 | GST-NTER | PHLKLYGNILCPYVQRVRFALEALKLQYDYVEIDLLaKKHLQeEYLAINPLGCVPTININNSNVYESLVLLEFLEEQFGNVF | 16.60 |
|  | GST-CTER | DTIKRAQQRIWANYYDQNVIGNiwdVFQVYKS-KDEEGLKKLANKlaEEIRywVKQTKLSERVKaNpKTFYEGEQLTYVDFAVVPHTRYLEDivrvtfnkQLFDLVENDDLIKDFRTYINNVTSSEAYNRIthKLPSLPA | 12.28 |
| TthGSTO5 | GST-NTER | SKLIFYGYLICPFAQRVRFALEALKIDYEFVHIDLHagAQKEEAYLKINPFGKLPSIQIDGKVVYESLILLEFLGDHFGGVF | 20.20 |
| TthGSTO6 | GST-NTER | KKPILYGCIVCPYTNKVRFALEMLKIDYKYQEIDILtgKNKNNSYLQINPQGRVPSLTTINnKNLYDSQVLLQYIEDEYKGLF | 15.08 |
|  | GST-CTER | DNYAKGLQRIWVQYFDNNIFSKYHPALAA----YQENKQDLLNKLQqetfENMKFFSQNSGitqKiklkqNcFYEGGSIPTYADVAIIPHLRIMNIfwkhflnkdlfQSNKNDEDVLSLKQLYENIHTLESCQNVSFQfKQIPTKG | 9.15 |
| TthGSTO7 | GST-NTER | EPVRFYCFVTCPYAIRVRTALELLQVPYEYNEIDLLtiseslvcleylNDKYQpgLLPQDsFQRAQIRKWITYYssidakkwrilgairnknkeeayRILNEIQQNLKFLSSQIDLPI | 8.60 |
|  | GST-CTER | DSFQRAQIRKWITYYSSIDAKKwrILGAIR---NKNKEEAYRILNEIQQNLKFLSSQIDlpirveQNskTFLFGSTFGMGDIAILPVLDQMIIlfetafgkhilkdnLNGKDVDALKALYIWFENTKQQPAYQKSTY-KlqsLPQNPL | 13.11 |
| TthGSTO8 | GST-NTER | KKIKIYQSLSCPFCQKVKLAMELLSVPYFQYEIDIAnFEHEQeWFKQINPEQTVPALILTDnTPAFESFPFIKKLDSENVNVF | 12.27 |
| TthGSTZ1 | GST-NTER | KKITLYSYFRSSTSWRVRIALNLKKIDYNIIPINLLksEQTSEEYTKINPNQGVPALKYGDEVIIESSAILEFLEEVFPEHP | 22.81 |
|  | GST-CTER | DAVKRAQIRGFCQVINTAIHP-LQNLRVLnkiEKEYSQDKIQWLKFWVTKGLTAIEELLKNsHgKYCFGDEITLADLFLVPQVQGVVDRFQFDLTPFPNIAEVLKNLKEIPEFVAASPSKQADNPD | 21.27 |
| TthGSTZ2 | GST-NTER | QNIILYGNYRSSPSWRVRLALGLKKIEYKLVSIDLFknEQKSEVYFKVNPNQRIPALIYGDQTLIESTAIIEFLEENFPQYP | 22.40 |
|  | GST-CTER | DRIKRAQIRGFCQVINSAIHPYqNSNLIGKiEKEGNMNKLEWIKFWVTKGLTAIEELLKKyHgKFCFGDDITMADIFLIPQVSAVVERFGFDLTPFPLILSVVNNLKDLPEFIAASPSNQPDYTE | 18.77 |
| TthGSTN1 | GST-NTER | SELQILGPLGNIHINIALIIAELAGVPLKHVVVEHKEATGKEFVKKYPLGLIPILITPDrETILTPVAIFKYIARAGKQLL | 10.55 |
|  | GST-CTER | SPLDETKIDQFLDIILGNLHKSyedITTSIYGYREYDETSVKNAKKLFEKNLKFINDALKiD-TYLVGQKLSIVDIALAAVLHRTFKiaFDDKFRSENPHTVRHLRYFSSIPVFSKYFGRltlaSgdwAPAKKA | 17.13 |
| TthGSTN2 | GST-NTER | MTYQLLAPAGNFRANMLLTIAELVGVKLELVHTEYAaTKTPEFKQKNPLGKVPVLITPEGPVYESNAIARHLARTAGKLY | 17.01 |
|  | GST-CTER | NQHEAALVDQYLDMAVTELLPSLTTTLYAIFGFKPADKEVLKaAKQEtfSVLRILNERLTHHKYLAGENLTIADIQLATFLNLAFRvtISGEQKKPIAKVVEYFVRVAQLPEFTKYHGRPhFATSEF | 16.94 |
|  | EF1G-CTER | EEEEKEEQPASGWNLYDYKTLYVNAKNKEEAIQNLVENFDAKTMCIYHLHYqKYTGDGQVLYLFNNMKNNFLQRCDPARKVAFGTYSIYGEEPNLEISGVWLFMGATIPPQMNENPSFEYHDLKQLDITKAEDLQILRNYWTNTEEDTSvvdGLRLRSFGSFK | 23.97 |

GST-NTER: Cytosolic glutathione S-transferase N-terminal domain profile. GST-CTER: Cytosolic glutathione S-transferase C-terminal domain profile. EF1G-CTER: Elongation factor 1 (EF-1) gamma C-terminal domain profile. Conserved motifs in the N- and C-terminal domains are shown shaded in yellow and blue, respectively. P (Proline) shaded in green is the cis-Pro-loop (see text).

**Table S6.** β-sheets/α-helixes patterns of putative cytosolic TthGSTs.

| TthGST | β-sheets/α-helixes configuration |
| --- | --- |
| TthGSTM1 | NTER: α1β1α2β2α3α4β3β4α5α6  CTER: 7 α-helixes |
| TthGSTM2 | NTER: α1β1α2β2α3α4β3β4α5α6  CTER: 7 α-helixes |
| TthGSTM3 | NTER: β1α1β2α2α3α4  CTER: 7 α-helixes |
| TthGSTM4 | NTER: α1β1α2β2α3β3β4α4α5  CTER: 7 α-helixes |
| TthGSTM5 | NTER: β1α1β2α2α3β3β4α4α5  CTER: 7 α-helixes |
| TthGSTM6 | NTER: α1β1α2β2α3β3α4α5β4β5α6  CTER: 9 α-helixes |
| TthGSTM7 | NTER: β1α1β2α2α3β3β4α4α5α6  CTER: 7 α-helixes |
| TthGSTM8 | NTER: β1α1β2α2α3β3β4α4α5α6  CTER: 6 α-helixes |
| TthGSTM9 | NTER: β1α1β2α2α3β3β4α4α5α6  CTER: 7 α-helixes |
| TthGSTM10 | NTER: β1α1β2α2α3β3β4α4  CTER: 7 α-helixes |
| TthGSTM11 | NTER: β1α1β2α2β3β4α3α4  CTER: 7 α-helixes |
| TthGSTM12 | NTER: β1α1β2α2β3β4α3  CTER: 6 α-helixes |
| TthGSTM13 | NTER: β1α1β2α2β3β4α3α4  CTER: 8 α-helixes |
| TthGSTM14 | NTER: β1α1β2α2β3β4α3α4  CTER: 7 α-helixes |
| TthGSTM15 | NTER: α1β1α2β2α3α4β3β4α5  CTER: 9 α-helixes |
| TthGSTM16 | NTER: β1α1β2α2α3β3β4α4α5  CTER: 7 α-helixes |
| TthGSTM17 | NTER: β1α1β2α2α3β3β4α4  CTER: 7 α-helixes |
| TthGSTM18 | NTER: β1α1β2α2α3β3β4α4  CTER: 5 α-helixes |
| TthGSTM19 | NTER: β1α1β2α2α3β3β4α4α5  CTER: 7 α-helixes |
| TthGSTM20 | NTER: β1α1β2α2β3β4α3α4  CTER: 7 α-helixes |
| TthGSTM21 | NTER: β1α1β2α2α3β3β4α4α5  CTER: 8 α-helixes |
| TthGSTM22 | NTER: β1α1β2α2α3β3β4α4α5  CTER: 7 α-helixes |
| TthGSTM23 | NTER: β1α1β2α2α3β3β4α4  CTER: 9 α-helixes |
| TthGSTM24 | NTER: β1α1β2α2α3β3β4α4α5  CTER: 7 α-helixes |
| TthGSTM25 | NTER: β1α1β2α2α3β3β4α4α5  CTER: 7 α-helixes |
| TthGSTM26 | NTER: β1α1β2α2β3β4α3α4  CTER: 7 α-helixes |
| TthGSTM27 | NTER: β1α1β2α2α3β3β4α4  CTER: 7 α-helixes |
| TthGSTM28 | NTER: β1α1β2α2α3β3β4α4α5  CTER: 7 α-helixes |
| TthGSTM29 | NTER: β1α1β2α2α3β3β4α4α5  CTER: 9 α-helixes |
| TthGSTM30 | NTER: β1α1β2α2α3β3β4α4  CTER: 7 α-helixes |
| TthGSTM31 | NTER: 8 α-helixes (α1-α8)  CTER: α9β1α10β2α11 + 10 α-helixes (α12-α21) + β3α22 |
| TthGSTM32 | NTER: β1α1β2α2α3β3β4α4α5  CTER: 8 α-helixes |
| TthGSTM33 | NTER: β1α1β2α2α3β3α4α5  CTER: 8 α-helixes |
| TthGSTM34 | NTER: β1α1α2β2α3β3α4α5β4  CTER: 10 α-helixes |
| TthGSTM35 | NTER: β1α1β2α2α3β3α4α5  CTER: 9 α-helixes |
| TthGSTM36 | NTER: 6 β-sheets  CTER: 10 β-sheets + 1 α-helix |
| TthGSTM37 | NTER: β1α1β2α2α3β3β4α4α5  CTER: 9 α-helixes |
| TthGSTM38 | NTER: β1α1β2α2α3β3α4α5  CTER: 9 α-helixes |
| TthGSTM39 | NTER: β1α1β2α2α3β3α4α5  CTER: 8 α-helixes |
| TthGSTM40 | NTER: 17 α-helixes + 4 β-sheets  CTER: 25 α-helixes + 6 β-sheets |
| TthGSTM41 | NTER: β1α1β2α2α3β3α4α5  CTER: 8 α-helixes |
| TthGSTM42 | NTER: β1α1α2β2α3β3α4α5β4β5α6  CTER: 8 α-helixes |
| TthGSTM43 | NTER: β1α1β2α2α3β3α4α5  CTER: 8 α-helixes |
| TthGSTM44 | NTER: β1α1β2α2α3β3β4α4α5  CTER: 8 α-helixes |
| TthGSTM45 | NTER: β1α1β2α2α3β3β4α4α5  CTER: 9 α-helixes |
| TthGSTM46 | NTER: β1α1β2α2α3β3β4α4α5  CTER: 9 α-helixes |
| TthGSTM47 | NTER: β1α1β2α2α3β3β4α4α5  CTER: 10 α-helixes |
| TthGSTM48 | NTER: β1α1β2α2α3β3α4α5  CTER: 7 α-helixes |
| TthGSTM49 | NTER: β1α1β2α2α3β3β4α4α5  CTER: 7 α-helixes |
| TthGSTM50 | NTER: β1α1β2α2α3β3β4α4α5  CTER: 9 α-helixes |
| TthGSTM51 | NTER: β1α1β2α2α3β3β4α4α5  CTER: 9 α-helixes |
| TthGSTM52 | Complete protein: α1β1α2β2α3β3 |
| TthGSTM53 | NTER: **α1β1β2α2β3α3**β4  CTER: β5β6α4β7β8β9β10 |
| TthGSTM54 | NTER: **α1β1β2α2β3α3**  CTER: β4β5α4β6β7α5β8β9β10 |
| TthGSTO1 | NTER: β1α1β2α2α3α4β3β4α5α6  CTER: 9 α-helixes |
| TthGSTO2 | NTER: β1α1β2α2β3β4α3α4  CTER: 8 α-helixes |
| TthGSTO3 | NTER: β1α1β2α2α3β3β4α4α5  CTER: 11 α-helixes |
| TthGSTO4 | NTER: β1α1β2α2α3β3β4α4α5  CTER: 8 α-helixes |
| TthGSTO5 | NTER: β1α1β2α2β3β4α3α4α5  CTER: 8 α-helixes |
| TthGSTO6 | NTER: β1α1β2α2β3α3α4  CTER: 8 α-helixes |
| TthGSTO7 | NTER: β1α1β2α2α3α4  CTER: 7 α-helixes |
| TthGSTO8 | NTER: β1α1β2α2α3β3α4α5α6  CTER: 8 α-helixes |
| TthGSTT1 | NTER: β1α1β2α2β3α3α4  CTER: 6 α-helixes |
| TthGSTT2 | NTER: β1α1β2α2β3α3α4  CTER: 7 α-helixes |
| TthGSTT3 | NTER: β1α1β2α2β3α3α4  CTER: 6 α-helixes |
| TthGSTT4 | NTER: β1α1β2α2α3β3β4α4α5  CTER: α6α7β5 + 5 α-helixes |
| TthGSTT5 | NTER: β1α1β2α2α3β3β4α4α5  CTER: 8 α-helixes |
| TthGSTZ1 | NTER: β1α1β2α2α3β3β4α4α5  CTER: 5 α-helixes |
| TthGSTZ2 | NTER: β1α1β2α2β3β4α3α4  CTER: 5 α-helixes |
| TthGSTN1 | NTER: β1α1β2α2α3β3β4α4α5  CTER: 6 α-helixes |
| TthGSTN2 | NTER: β1α1β2α2β3β4α3α4α5α6  α7α8α9α10  CTER:α11α12α13α14α15β5α16β6β7α17  α18β8β9α19α20β10β11 |

Beta-sheet/alpha-helix configuration of N-terminal (NTER) and C-terminal (CTER) domains. GST name green shading: non GST-domain proteins. β-sheets/α-helixes NTER configuration shaded in yellow: canonical thioredoxin folding. β-sheets/α-helixes NTER configuration shaded in blue: slightly altered canonical thioredoxin folding.

**Table S7**. Number of potential selected transcription factor binding sites in the promoter regions of cytosolic *TthGST* genes.

(A)- bZIP transcription factors superfamily.

| Gene | c-Jun | Jun B | Jun D | c-Fos | Nrf2/MafK | Total |
| --- | --- | --- | --- | --- | --- | --- |
| *TthGSTM1* | 3 | 1 | 1 | 3 | 1 | 9 |
| *TthGSTM2* | 3 | 1 | 1 | 3 | 2 | 10 |
| *TthGSTM3* | 3 | 1 | 1 | 3 | 2 | 10 |
| *TthGSTM4* | 6 | 2 | 2 | 6 | 2 | 18 |
| *TthGSTM5* | - | - | - | - | 1 | 1 |
| *TthGSTM6* | 6 | 2 | 2 | 6 | 4 | 20 |
| *TthGSTM7* | 3 | 1 | 1 | 3 | 1 | 9 |
| *TthGSTM8* | 3 | 1 | 1 | 3 | 2 | 10 |
| *TthGSTM9* | - | - | 1 | - | 2 | 3 |
| *TthGSTM10* | - | - | - | - | 1 | 1 |
| *TthGSTM11* | - | - | - | - | 1 | 1 |
| *TthGSTM12* | 6 | 2 | 2 | 6 | 4 | 20 |
| *TthGSTM13* | 6 | 2 | 2 | 5 | 3 | 18 |
| *TthGSTM14* | - | - | - | - | 1 | 1 |
| *TthGSTM15* | 6 | 2 | 2 | 6 | - | 16 |
| *TthGSTM16* | 3 | 1 | 1 | 3 | 2 | 10 |
| *TthGSTM17* | 3 | 1 | 1 | 3 | 4 | 12 |
| *TthGSTM18* | 3 | 1 | 1 | 3 | 1 | 9 |
| *TthGSTM19* | 9 | 3 | 3 | 9 | 1 | 25 |
| *TthGSTM20* | 3 | 1 | 1 | 3 | 2 | 10 |
| *TthGSTM21* | 3 | 1 | 1 | 3 | 3 | 11 |
| *TthGSTM22* | - | - | - | - | 1 | 1 |
| *TthGSTM23* | - | - | - | - | 1 | 1 |
| *TthGSTM24* | - | - | - | - | 4 | 4 |
| *TthGSTM25* | - | - | - | - | 3 | 3 |
| *TthGSTM26* | 6 | 2 | 2 | 6 | 2 | 18 |
| *TthGSTM27* | - | - | - | - | 2 | 2 |
| *TthGSTM28* | 3 | 1 | 1 | 3 | 3 | 11 |
| *TthGSTM29* | - | - | - | - | 4 | 4 |
| *TthGSTM30* | 3 | 1 | 1 | 3 | 4 | 12 |
| *TthGSTM31* | - | - | - | - | - | - |
| *TthGSTM32* | - | - | - | - | 1 | 1 |
| *TthGSTM33* | - | - | - | - | 1 | 1 |
| *TthGSTM34* | 6 | 2 | 2 | 6 | 2 | 18 |
| *TthGSTM35* | 6 | 2 | 2 | 5 | 1 | 16 |
| *TthGSTM36* | - | - | - | - | 1 | 1 |
| *TthGSTM37* | 3 | 1 | 1 | 3 | 3 | 11 |
| *TthGSTM38* | 6 | 2 | 2 | 6 | 1 | 17 |
| *TthGSTM39* | 6 | 2 | 2 | 6 | 2 | 18 |
| *TthGSTM40* | 6 | 2 | 2 | 6 | 1 | 17 |
| *TthGSTM41* | - | - | - | - | 2 | 2 |
| *TthGSTM42* | - | - | - | - | - | - |
| *TthGSTM43* | 12 | 4 | 4 | 12 | 1 | 33 |
| *TthGSTM44* | 3 | 1 | 1 | 3 | 1 | 9 |
| *TthGSTM45* | - | - | - | - | 6 | 6 |
| *TthGSTM46* | 6 | 2 | 2 | 6 | 2 | 18 |
| *TthGSTM47* | - | - | - | - | - | - |
| *TthGSTM48* | 9 | 3 | 3 | 9 | 2 | 26 |
| *TthGSTM49* | - | - | - | - | 1 | 1 |
| *TthGSTM50* | 3 | 1 | 1 | 3 | 1 | 9 |
| *TthGSTM51* | - | - | - | - | 3 | 3 |
| *TthGSTM52* | 6 | 2 | 2 | 6 | 2 | 18 |
| *TthGSTM53* | 3 | 1 | 1 | 3 | - | 8 |
| *TthGSTM54* | 9 | 3 | 3 | 9 | 2 | 26 |
| *TthGSTT1* | 12 | 4 | 4 | 12 | 2 | 34 |
| *TthGSTT2* | 6 | 2 | 2 | 6 | - | 16 |
| *TthGSTT3* | - | - | - | - | - | - |
| *TthGSTT4* | - | - | - | - | - | - |
| *TthGSTT5* | - | - | 1 | - | - | 1 |
| *TthGSTO1* | - | - | - | - | 2 | 2 |
| *TthGSTO2* | - | - | - | - | 3 | 3 |
| *TthGSTO3* | - | - | - | - | 2 | 2 |
| *TthGSTO4* | - | - | - | - | 1 | 1 |
| *TthGSTO5* | 6 | 2 | 2 | 6 | 1 | 17 |
| *TthGSTO6* | 6 | 2 | 3 | 6 | - | 17 |
| *TthGSTO7* | 3 | 1 | 1 | 3 | 1 | 9 |
| *TthGSTO8* | 6 | 2 | 2 | 6 | 2 | 18 |
| *TthGSTZ1* | 3 | 1 | 1 | 3 | 3 | 11 |
| *TthGSTZ2* | 3 | 1 | 1 | 3 | 3 | 11 |
| *TthGSTN1* | - | - | - | - | 2 | 2 |
| *TthGSTN2* | 6 | 2 | 3 | 6 | - | 17 |
| Average | **3** | **0.7** | **0.8** | **3** | **1.7** | **9** |
| Total % | **32.7%** | **8.4%** | **8.6%** | **32.4%** | **18.2%** |  |

(B)- C4 zinc finger-type transcription factor.

| Gene | GATA-1 | GATA-2 | GATA-3 | GATA-6 | Total |
| --- | --- | --- | --- | --- | --- |
| *TthGSTM1* | 4 | 2 | 1 | - | 7 |
| *TthGSTM2* | 4 | 1 | - | - | 5 |
| *TthGSTM3* | 10 | 3 | 3 | - | 16 |
| *TthGSTM4* | 12 | 3 | 2 | - | 17 |
| *TthGSTM5* | 18 | 4 | 2 | - | 24 |
| *TthGSTM6* | 18 | 7 | 2 | - | 27 |
| *TthGSTM7* | 9 | 2 | - | - | 11 |
| *TthGSTM8* | 5 | 3 | 1 | - | 9 |
| *TthGSTM9* | 12 | 2 | 2 | - | 16 |
| *TthGSTM10* | 4 | 1 | 1 | - | 6 |
| *TthGSTM11* | 18 | 5 | 2 | - | 25 |
| *TthGSTM12* | 6 | 3 | 1 | - | 10 |
| *TthGSTM13* | 16 | 6 | 4 | - | 26 |
| *TthGSTM14* | 10 | 3 | 2 | - | 15 |
| *TthGSTM15* | 10 | 2 | 3 | - | 15 |
| *TthGSTM16* | 32 | 8 | 4 | - | 44 |
| *TthGSTM17* | 24 | 6 | 4 | - | 34 |
| *TthGSTM18* | 15 | 4 | 5 | - | 24 |
| *TthGSTM19* | 6 | 2 | 2 | - | 10 |
| *TthGSTM20* | 16 | 5 | - | - | 21 |
| *TthGSTM21* | 17 | 6 | - | - | 23 |
| *TthGSTM22* | 16 | 6 | - | - | 22 |
| *TthGSTM23* | 18 | 4 | - | - | 22 |
| *TthGSTM24* | 19 | 4 | - | - | 23 |
| *TthGSTM25* | 16 | 3 | - | - | 19 |
| *TthGSTM26* | 16 | 5 | - | - | 21 |
| *TthGSTM27* | 24 | 6 | - | - | 30 |
| *TthGSTM28* | 6 | 1 | - | - | 7 |
| *TthGSTM29* | 14 | 3 | - | - | 17 |
| *TthGSTM30* | 10 | 3 | - | - | 13 |
| *TthGSTM31* | 24 | 7 | 4 | - | 35 |
| *TthGSTM32* | 8 | 2 | - | - | 10 |
| *TthGSTM33* | 10 | 3 | 1 | - | 14 |
| *TthGSTM34* | 18 | 4 | 3 | - | 25 |
| *TthGSTM35* | 14 | 3 | 2 | - | 19 |
| *TthGSTM36* | 5 | 1 | 2 | - | 8 |
| *TthGSTM37* | 20 | 4 | 3 | - | 27 |
| *TthGSTM38* | 14 | 4 | 2 | - | 20 |
| *TthGSTM39* | 20 | 5 | 5 | - | 30 |
| *TthGSTM40* | 10 | 2 | 1 | - | 13 |
| *TthGSTM41* | 12 | 3 | 2 | - | 17 |
| *TthGSTM42* | 26 | 5 | 3 | - | 34 |
| *TthGSTM43* | 18 | 5 | 4 | 1 | 28 |
| *TthGSTM44* | 12 | 2 | 3 | - | 17 |
| *TthGSTM45* | 20 | 4 | 6 | - | 30 |
| *TthGSTM46* | 13 | 4 | 2 | 1 | 20 |
| *TthGSTM47* | 10 | 3 | 5 | - | 18 |
| *TthGSTM48* | 21 | 8 | 4 | - | 32 |
| *TthGSTM49* | 20 | 5 | 3 | - | 28 |
| *TthGSTM50* | 18 | 4 | 1 | - | 23 |
| *TthGSTM51* | 10 | 2 | 2 | - | 14 |
| *TthGSTM52* | 6 | 2 | 1 | - | 9 |
| *TthGSTM53* | 19 | 5 | 4 | - | 28 |
| *TthGSTM54* | 19 | 4 | 3 | - | 26 |
| *TthGSTT1* | 16 | 5 | 2 | 1 | 24 |
| *TthGSTT2* | 33 | 9 | 3 | - | 45 |
| *TthGSTT3* | 16 | 4 | 1 | - | 21 |
| *TthGSTT4* | 10 | 4 | 2 | - | 16 |
| *TthGSTT5* | 16 | 5 | 2 | - | 23 |
| *TthGSTO1* | 6 | 2 | 1 | - | 9 |
| *TthGSTO2* | 8 | 2 | 2 | - | 12 |
| *TthGSTO3* | 8 | 2 | - | - | 10 |
| *TthGSTO4* | 14 | 3 | 3 | - | 20 |
| *TthGSTO5* | 26 | 9 | 7 | - | 42 |
| *TthGSTO6* | 34 | 8 | 7 | - | 49 |
| *TthGSTO7* | 9 | 2 | 1 | - | 12 |
| *TthGSTO8* | 13 | 4 | 5 | - | 22 |
| *TthGSTZ1* | 17 | 3 | 4 | - | 24 |
| *TthGSTZ2* | 21 | - | 4 | - | 25 |
| *TthGSTN1* | 13 | 4 | 2 | - | 19 |
| *TthGSTN2* | 24 | 4 | 8 | - | 36 |
| Average | **14** | **4** | **2** | **0** | **20** |
| Total % | **71.6%** | **18.6%** | **10.9%** | **0.20%** |  |

Names in red non GST-domain proteins.

**Table S8**. Several features of putative ciliate MAPEG-GST genes and proteins.

| **Ciliate** | **Gene number** | **Number of genes with introns (%)** | **Intron number**  **/(gen)** | **Average protein size (aa)** | **Average molecular mass (KD)** |
| --- | --- | --- | --- | --- | --- |
| *T. thermophila* | 4 | 1 (25%) | 2(1) | 186 | 21.06 |
| *T. borealis* | 2 | 0 | 0 | 194 | 22.03 |
| *T. canadensis* | 2 | 0 | 0 | 194 | 22.04 |
| *T. elliotti* | 2 | 0 | 0 | 196 | 22.43 |
| *T. empidokyrea* | 1 | 0 | 0 | 178 | 20.17 |
| *T. malaccensis* | 2 | 0 | 0 | 196 | 22.54 |
| *T. paravorax* | 2 | 0 | 0 | 197 | 22.60 |
| *T. pyriformis* | 2 | 0 | 0 | 195 | 22.01 |
| *T. shanghaiensis* | 1 | 0 | 0 | 178 | 20.11 |
| *T. vorax* | 2 | 0 | 0 | 195 | 22.15 |
|  | | | | | |
| *Ichthyophthirius multifiliis* | 2 | 2 (100%) | 3(1), 9(1) | 358 | 41.18 |
| *Blepharisma stoltei* | 5 | 0 | 0 | 174 | 19.87 |
| *Pseudokeronopsis carnea* | 11 | 4 (36%) | 1(4) | 164 | 18.38 |
| *Pseudokeronopsis flava* | 7 | 1 (14%) | 1(1) | 155 | 17.31 |
| *Stentor coeruleus* | 1 | 0 | 0 | 177 | 19.95 |
| *Oxytricha trifallax* | 11 | 4 (36%) | 1(4) | 214 | 25,56 |
| *Stylonychia lemnae* | 6 | 2 (33%) | 1(1), 3(1) | 290 | 33.48 |

**Table S9.** α-helixes from putative *Tetrahymena* MAPEG-GSTs.

| ***Tetrahymena* species** | **Average number of**  **α-helixes** | **Average number of transmembrane α-helixes** |
| --- | --- | --- |
| *T. thermophila* | 7 | 4 |
| *T. borealis* | 6 | 5 |
| *T. canadensis* | 5 | 4 |
| *T. elliotti* | 7 | 4 |
| *T. empidokyrea* | 7 | 4 |
| *T. malaccensis* | 6 | 4 |
| *T. paravorax* | 6 | 4 |
| *T. pyriformis* | 5 | 4 |
| *T. shanghaiensis* | 5 | 4 |
| *T. vorax* | 6 | 4 |
| **Total average** | **6** | **4** |

**Table S10.** Unclassified (N) GST domains from ciliates and several selected parasitic protozoa.

| GSTN | Domains | Protein size (aa) | Molecular mass (KD) |
| --- | --- | --- | --- |
| TthGSTN1 | GST-NTER, GST-CTER | 221 | 24.79 |
| TthGSTN2 | GST-NTER, GST-CTER, EF1G-CTER | 410 | 46.48 |
| TboGSTN1 | GST-NTER, GST-CTER, EF1G-CTER | 416 | 47.41 |
| TboGSTN2 | GST-NTER, GST-CTER | 221 | 24.60 |
| TcaGSTN1 | GST-NTER, GST-CTER, EF1G-CTER | 416 | 47.42 |
| TcaGSTN2 | GST-NTER, GST-CTER | 221 | 24.60 |
| TelGSTN1 | GST-NTER, GST-CTER | 222 | 24.89 |
| TelGSTN2 | GST-NTER, GST-CTER, EF1G-CTER | 412 | 46.78 |
| TemGSTN1 | GST-NTER, GST-CTER | 221 | 24.61 |
| TemGSTN2 | GST-NTER, GST-CTER, EF1G-CTER | 421 | 47.28 |
| TmaGSTN1 | GST-NTER, GST-CTER, EF1G-CTER | 413 | 46.84 |
| TmaGSTN2 | GST-NTER, GST-CTER | 221 | 24.72 |
| TpvGSTN1 | GST-NTER, GST-CTER | 201 | 22.82 |
| TpvGSTN2 | GST-NTER, GST-CTER, EF1G-CTER | 417 | 47.70 |
| TpvGSTN3 | GST-NTER, GST-CTER, EF1G-CTER | 408 | 46.85 |
| TpyGSTN1 | GST-NTER, GST-CTER | 221 | 24.61 |
| TshGSTN1 | GST-NTER, GST-CTER | 225 | 25.17 |
| TshGSTN2 | GST-NTER, GST-CTER, EF1G-CTER | 475 | 53.36 |
| TvoGSTN1 | GST-NTER, GST-CTER, EF1G-CTER | 413 | 47.04 |
| TvoGSTN2 | GST-NTER, GST-CTER | 224 | 24.82 |
| PteGSTN1 | GST-NTER, GST-CTER | 169 | 19.80 |
| PteGSTN2 | GST-NTER, GST-CTER, EF1G-CTER | 424 | 49.08 |
| EvaGSTN1 | GST-NTER, GST-CTER | 244 | 27.42 |
| EvaGSTN2 | GST-NTER, GST-CTER, EF1G-CTER | 416 | 47.47 |
|  |  |  |  |
| TbrGSTN1 | GST-NTER | 351 | 38.14 |
| TcrGSTN1 | 2 GST-NTER, 2 GST-CTER | 445 | 50.68 |
| PfaGSTN1 | GST-NTER, GST-CTER | 213 | 25.03 |
| PfaGSTN2 | GST-NTER, GST-CTER | 111 | 13.15 |
| PfaGSTN3 | GST-CTER | 204 | 24.02 |
| PfaGSTN4 | GST-NTER, GST-CTER | 110 | 12.81 |
| PfaGSTN5 | GST-NTER | 212 | 24.91 |

GST-NTER: Soluble glutathione S-transferase N-terminal domain profile. GST-CTER: Soluble glutathione S-transferase C-terminal domain profile. EF1G-CTER: Elongation factor 1 (EF-1) gamma C-terminal domain profile. Tth: *T. thermophila*, Tbo: *T. borealis*, Tca: *T. canadensis*, Tel: *T. elliotti*, Tem: *T. empidokyrea*, Tma: *T. malaccensis*, Tpv: *T. paravorax*, Tpy: *T. pyrimorfis*, Tsh: *T. shanghaiensis*, Tvo: *T. vorax*, Pte: *Paramecium tetraurelia*, Eva: *Euplotes vanus*, Tbr: *Trypanosoma brucei*, Tcr: *T. cruzi*, Pfa: *Plasmodium falciparum*.
