# Supplementary material for: Tetrahymena thermophila glutathione-S-transferase superfamily: an eco-paralogs gene network differentially responding to various environmental abiotic stressors and an update on this gene family in ciliates
Source: Front Genet. 2025 Mar 7;16:1538168. doi: 10.3389/fgene.2025.1538168 (PMC11925944; doi:10.3389/fgene.2025.1538168)
Supplement: Supplementary file 7 [file DataSheet11.pdf]

```
              ....|....| ....|....| ....|....| ....|....| ....|....|
                      10         20         30         40         50
TthGSTZ1      MAESSKKITL YSYFRSSTSW RVRIALNLKK IDYNIIPINL LKSEQTSEEY
TthGSTZ2      MSENNQNIIL YGNYRSSPSW RVRLALGLKK IEYKLVSIDL FKNEQKSEVY
                           G-site

              ....|....| ....|....| ....|....| ....|....| ....|....|
                      60         70         80         90        100
TthGSTZ1      TKINPNQGVP ALKYGDEVII ESSAILEFLE EVFPEHPLLP QDAVKRAQIR
TthGSTZ2      FKVNPNQRIP ALIYGDQTLI ESTAIIEFLE ENFPQYPLLP EDRIKRAQIR
```

FIGURE S11

Partial alignment of Zeta class TthGSTs. Shaded in yellow: identical amino acid residues. Inside red box: conserved motif in the GST-NTER domains (putative G-site). Shaded in green: cis-Proline-loop (see text). Shaded in light blue serine residues (C). Gray shading tyrosine residues (Y).
