# Supplementary figures and images for: Tetrahymena thermophila glutathione-S-transferase superfamily: an eco-paralogs gene network differentially responding to various environmental abiotic stressors and an update on this gene family in ciliates
Source: Front Genet. 2025 Mar 7;16:1538168. doi: 10.3389/fgene.2025.1538168 (PMC11925944; doi:10.3389/fgene.2025.1538168)

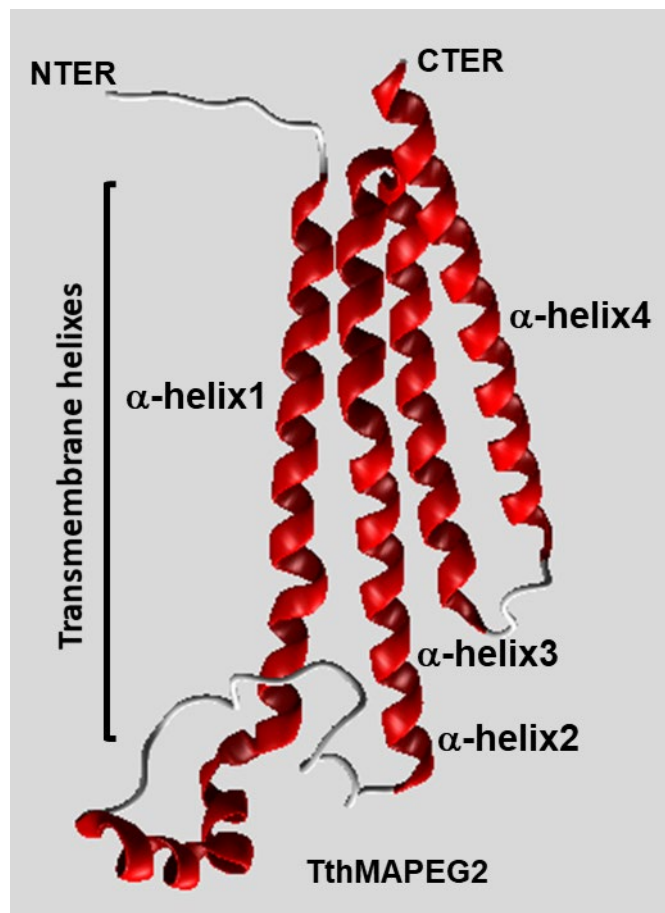

**FIGURE S3**

3D structure of TthMAPEG2 (for further explanations see text).

Supplement: Supplementary file 8 [file DataSheet3.pdf]
