# Supplementary material for: Tetrahymena thermophila glutathione-S-transferase superfamily: an eco-paralogs gene network differentially responding to various environmental abiotic stressors and an update on this gene family in ciliates
Source: Front Genet. 2025 Mar 7;16:1538168. doi: 10.3389/fgene.2025.1538168 (PMC11925944; doi:10.3389/fgene.2025.1538168)
Supplement: Supplementary file 9 [file DataSheet1.pdf]

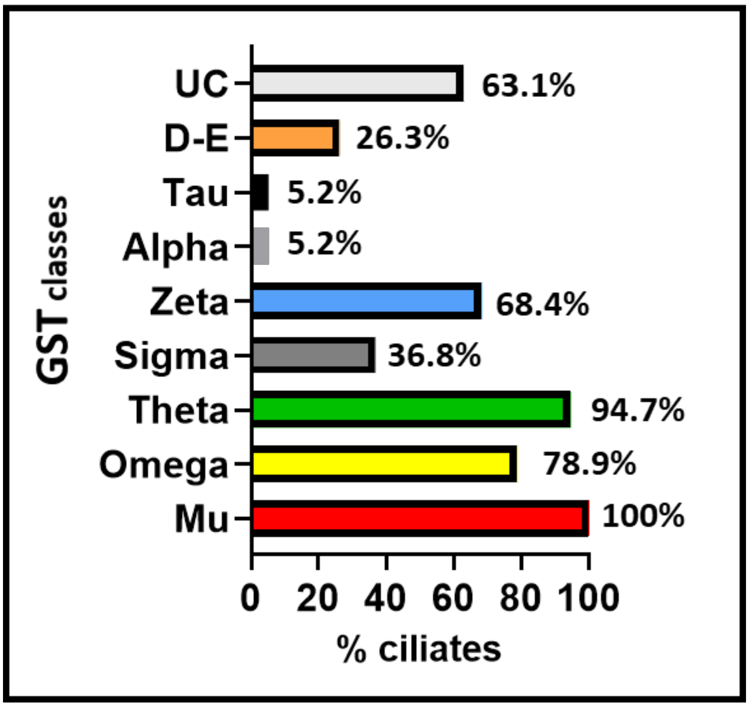

FIGURE S1

Histogram showing the percentage of cGST classes in ciliates. D-E (Delta-Epsilon). UC (unclassified).
