# Supplementary material for: Tetrahymena thermophila glutathione-S-transferase superfamily: an eco-paralogs gene network differentially responding to various environmental abiotic stressors and an update on this gene family in ciliates
Source: Front Genet. 2025 Mar 7;16:1538168. doi: 10.3389/fgene.2025.1538168 (PMC11925944; doi:10.3389/fgene.2025.1538168)
Supplement: Supplementary file 10 [file DataSheet5.pdf]

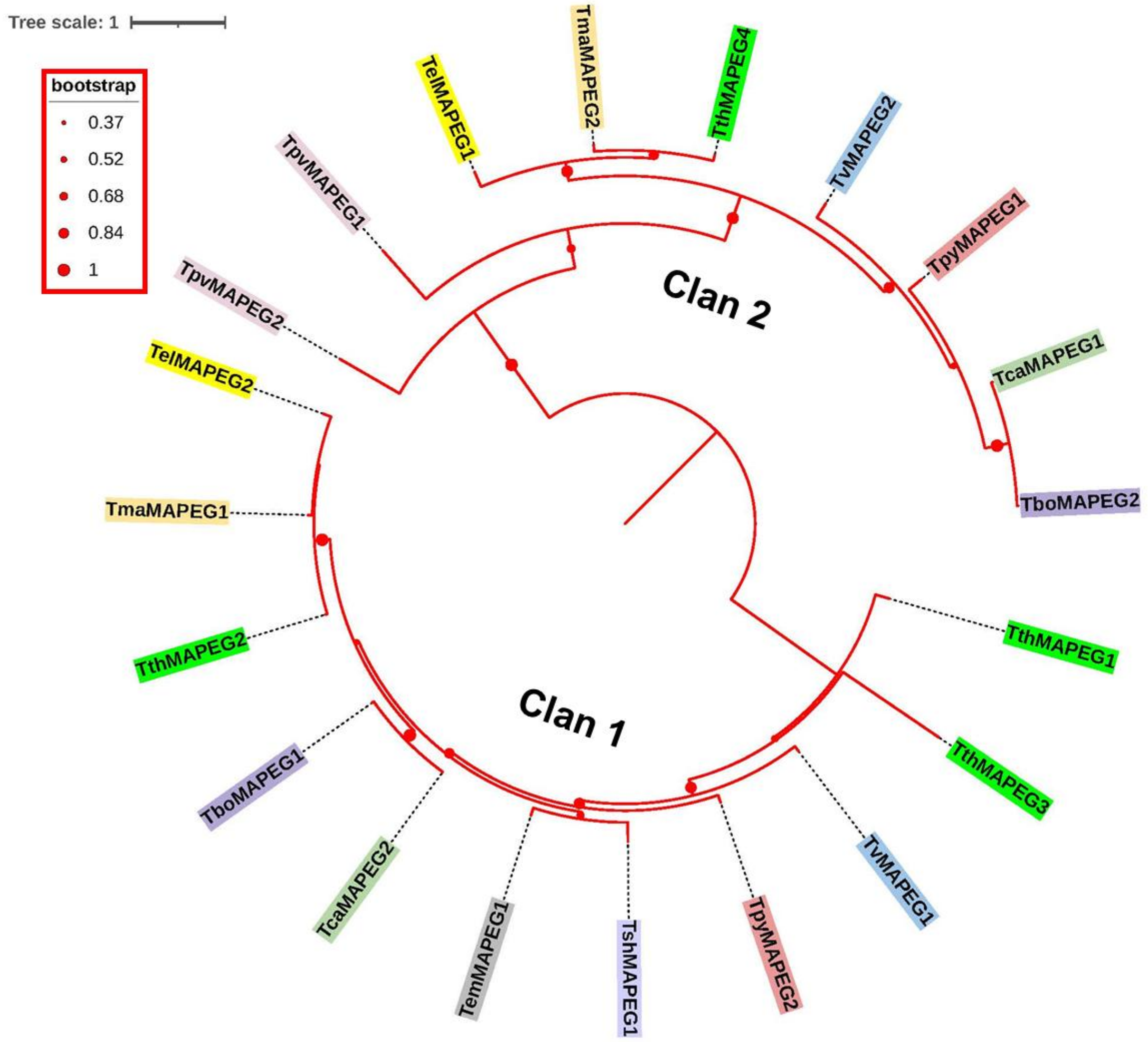

FIGURE S5

Phylogran of the MAPEG proteins from *Tetrahymena* species. Members of each species are shaded with the same color. For further information see text. Each branch length follows the scale. Calculated bootstrap values, from 2000 replicates, are indicated as spheres of different sizes (values from 0.52 to 1).
