# Supplementary material for: Tetrahymena thermophila glutathione-S-transferase superfamily: an eco-paralogs gene network differentially responding to various environmental abiotic stressors and an update on this gene family in ciliates
Source: Front Genet. 2025 Mar 7;16:1538168. doi: 10.3389/fgene.2025.1538168 (PMC11925944; doi:10.3389/fgene.2025.1538168)
Supplement: Supplementary file 11 [file DataSheet8.pdf]

```
                ....|....| ....|....| ....|....| ....|....| ....|....|
                         410        420        430        440        450
TthGSTM1        ---------- ---------- ---------- ---------- ---MDSSNTT
TthGSTM2        ---------- ---------- ---------- ---------- ---MDSSNTT
TthGSTM3        ---------- ---------- ---------- ---------- ---MGCSNST
TthGSTM4        ---------- ---------- ---------- ---------- ---MGCQNST
TthGSTM5        ---------- ---------- ---------- ---------- ---MGCQNST
TthGSTM6        --------MI KLKKYNQSIN KTNKLINKKH QKSKKNKNKK IFQMGCQNST
TthGSTM7        ---------- ---------- ---------- ---------- ---MSSQTIN
TthGSTM8        ---------- ---------- ---------- ---------- ---MSSQTIN
TthGSTM9        ---------- ---------- ---------- ---------- ---MSSLTLN
TthGSTM10       ---------- ---------- ---------- ---------- ---MSSQTIN
TthGSTM11       ---------- ---------- ---------- ---------- ---MSSLTLN
TthGSTM12       ---------- ---------- ---------- ---------- ---MSSNTIT
TthGSTM13       ---------- ---------- ---------- ---------- ---MGCSGSS
TthGSTM14       ---------- ---------- ---------- ---------- ---MGCNQSS
TthGSTM15       ---------- ---------- ---------- ---------- ---MGCG-SS
TthGSTM16       ---------- ---------- ---------- ---------- ----------
TthGSTM17       ---------- ---------- ---------- ---------- ---MSN----
TthGSTM18       ---------- ---------- ---------- ---------- ---MSEL---
TthGSTM19       ---------- ---------- ---------- ---------- ----------
TthGSTM20       ---------- ---------- ---------- ---------- ----------
TthGSTM21       ---------- ---------- ---------- ---------- ----------
TthGSTM22       ---------- ---------- ---------- ---------- ----------
TthGSTM23       ---------- ---------- ---------- ---------- ----------
TthGSTM24       ---------- ---------- ---------- ---------- ----------
TthGSTM25       ---------- ---------- ---------- ---------- ----------
TthGSTM26       ---------- ---------- ---------- ---------- ----------
TthGSTM27       ---------- ---------- ---------- ---------- ----------
TthGSTM28       ---------- ---------- ---------- ---------- ----------
TthGSTM29       ---------- ---------- ---------- ---------- ----------
TthGSTM30       ---------- ---------- ---------- ---------- ----------
TthGSTM31       VLGFIWVIHQ AAIKRSEYLI AESITDVKMH MKLTVIYIAL ICLDSCVLRY
TthGSTM32       ---------- ---------- ---------- ---------- ----------
TthGSTM33       ---------- ---------- ---------- ---------- ----------
TthGSTM34       ---------- ---------- ----MCLYIY IYPPKRYNFQ KQQQINHKQN
TthGSTM35       ---------- ---------- ---------- ---------- ----------
TthGSTM36       -------MNS LSCDLCLPNF YYNEKNSQVH VDRNQVPVCI ECPQGSGTNQ
TthGSTM37       ---------- ---------- ---------- ---------- ----------
TthGSTM38       ---------- ---------- ---------- ---------- ----------
TthGSTM39       ---------- ---------- ---------- ---------- ----------
TthGSTM40       LINLVSVNIM RNIGYGLNFI FDFKVLVQRY MSIMSIENVQ MKSIDSNQTA
TthGSTM41       ---------- ---------- ---------- ---------- ----------
TthGSTM42       ---------- ---------M LVVIIYIYFY FIKFNKIQKA KINKQIIIQI
TthGSTM43       ---------- ---------- ---------- ---------- ----------
TthGSTM44       ---------- ---------- ---------- ---------- ----------
TthGSTM45       ---------- ---------- ---------- ---------- ----------
TthGSTM46       ---------- ---------- ---------- ---------- ----------
TthGSTM47       ---------- ---------- ---------- ---------- ----------
TthGSTM48       ---------- ---------- ---------- ---------- ----------
TthGSTM49       ---------- ---------- ---------- ---------- ----------
TthGSTM50       ---------- ---------- ---------- ---------- ----------
TthGSTM51       ---------- ---------- ---------- ---------- ----------
TthGSTM52       ---------- ---------- ---------- ---------- ---MDSSNTT
TthGSTM53       ---------- ---------- ---------- ---------- ----------
TthGSTM54       ---------- ---------- ---------- ---------- ----------
```

```
                ....|....| ....|....| ....|....| ....|....| ....|....|
                       460        470        480        490        500
TthGSTM1        NQIGKKQTQE SKELIIGYLE NASRGQ---- ---------- ----------
TthGSTM2        NQIGKKQTNE SKELIIGYLE NASRGQ---- ---------- ----------
TthGSTM3        GQVNDIQVKN KDNLVLGYWG LPLRGQ---- ---------- ----------
TthGSTM4        SLVNDIQAKN NDNLVLGYWG LPLRGQ---- ---------- ----------
TthGSTM5        GQVNDIQAKN SANLVLGYWG LPLRGQ---- ---------- ----------
TthGSTM6        GQVSTNEIKQ VEKVVLGYWA FPLRGQ---- ---------- ----------
TthGSTM7        ---------- -----LGYWG FPLRAQ---- ---------- ----------
TthGSTM8        ---------- -----LGYWG FPLRAQ---- ---------- ----------
TthGSTM9        ---------- -----LGFWA LPLRAQ---- ---------- ----------
TthGSTM10       ---------- -----LGFWA LPLRAQ---- ---------- ----------
TthGSTM11       ---------- -----LGYWA YPLRGQ---- ---------- ----------
TthGSTM12       ---------- -----LGYWA LPLRGQ---- ---------- ----------
TthGSTM13       AVKDKDCI-- NEKIVLGYWA VHLQGQ---- ---------- ----------
TthGSTM14       SENTIISSNA RKQLLLGYWN IPLRAQ---- ---------- ----------
TthGSTM15       SIQVAEVPNC NKQLLLGYWD IPLRAQ---- ---------- ----------
TthGSTM16       ---MDNKTCA QKQVTLGYWN IPLRAQ---- ---------- ----------
TthGSTM17       ---------- NDTLILGYWA QPVRAQ---- ---------- ----------
TthGSTM18       ---------- ----ILGYWG LPLKGQ---- ---------- ----------
TthGSTM19       --------MA D-KIVLGYWA GPGKAQ---- ---------- ----------
TthGSTM20       --------MA Y-KIVLGYWA GPGKAQ---- ---------- ----------
TthGSTM21       --------MS D-KIILGYWN TPGKAQ---- ---------- ----------
TthGSTM22       --------MS Q-KITIGYWK GPGKVQ---- ---------- ----------
TthGSTM23       --------MS --KIIIGQWA SAGKLV---- ---------- ----------
TthGSTM24       --------MT DNKLTLGYWE GYGKAQ---- ---------- ----------
TthGSTM25       ---------- ---MILGYWE IPGKCQ---- ---------- ----------
TthGSTM26       -MSKQSQENQ QTDLVLGYWP FAGRNM---- ---------- ----------
TthGSTM27       ---------- --MIILGYWN LRGYAQ---- ---------- ----------
TthGSTM28       ---------- --MIVLGYWN LRGYAQ---- ---------- ----------
TthGSTM29       ---------- --MIILGYWN LRGYGQ---- ---------- ----------
TthGSTM30       ---------- --MIILGYWT HRGFAQ---- ---------- ----------
TthGSTM31       MIKAFTNTSV DSYLILFFLE YLLISLN--- ---------- ----------
TthGSTM32       ---------- --MITLGYWN VRGLGQ---- ---------- ----------
TthGSTM33       ---------- --MITLGYWN ARGLGQ---- ---------- ----------
TthGSTM34       KTSLRNLIQY KQMTTLGYWG IRGLAQ---- ---------- ----------
TthGSTM35       ---------- --MITFGYWN IRGFGQ---- ---------- ----------
TthGSTM36       ANIDNFQISS CSICLQNYYM TNPSSQSPTL NSAQCLPCPN GTLSSGAQTV
TthGSTM37       ---------- --MITFGYWN IRGFGQ---- ---------- ----------
TthGSTM38       ---------- --MATLGYWG FRGLAQ---- ---------- ----------
TthGSTM39       ---------- -MQGTFGYWW VRGSAQ---- ---------- ----------
TthGSTM40       NSHPSGNTLV LMKDFYAYWA TENLDSK--- ---------- ----------
TthGSTM41       ---------- --MITLGYWG VRGLGE---- ---------- ----------
TthGSTM42       GIQINLQMAE KNQITLGYWN IRGLGQ---- ---------- ----------
TthGSTM43       --------MS EDKLILGYWP FRGVAQ---- ---------- ----------
TthGSTM44       ---------M TSKLIFGYWN GRGRGQ---- ---------- ----------
TthGSTM45       ---------M ASKLVYGYWN TRGRGQ---- ---------- ----------
TthGSTM46       ------MSNT EDKIILGYWD YRGRPQ---- ---------- ----------
TthGSTM47       ---------M KSQLLFGSWI QRGNAM---- ---------- ----------
TthGSTM48       -----MQQQQ QSKIKLGYWD VRGRGE---- ---------- ----------
TthGSTM49       ---------- MSNLILSYWN LRGRTE---- ---------- ----------
TthGSTM50       ---------- --MITFGYWN IRGFGQ---- ---------- ----------
TthGSTM51       ---------- --MFILGYWE SRGIAQ---- ---------- ----------
TthGSTM52       NQIGKKQIQE SQELIIGYLE NASIGQ---- ---------- ----------
TthGSTM53       ---------- --MNIFNIKL NLILAV---- ---------- ----------
TthGSTM54       ---------- --MNILKNNF KLACAI---- ---------- ----------
```

```
              ....|....| ....|....| ....|....| ....|....| ....|....|
                     510        520        530        540        550
TthGSTM1     ---------TV RYILDLVGFP YTEHKYTST- ---------- -----STEWE
TthGSTM2     ---------TV RYILDLVGYP YAEHKYTSS- ---------- -----STEWE
TthGSTM3     ---------PL RYILELANYP YTETKYTLSQ A--------- -----TDWFG
TthGSTM4     ---------PL RYILELANYP YTETKYTLSQ A--------- -----TDWFG
TthGSTM5     ---------PL RYILELANYP YTETKYTLSQ A--------- -----TDWFG
TthGSTM6     ---------PI RYILELANYP YTETKYTPST A--------- -----TEWFG
TthGSTM7     ---------PI RYIFELAQYP YQQTNYTFEG A--------- -----KDWFE
TthGSTM8     ---------PI RYIFELAQYP YQQTNYTFEG A--------- -----KDWFE
TthGSTM9     ---------PI RYIFELAQYP YQQTNYTFKE S--------- -----KEWFE
TthGSTM10    ---------PI RYIFELAQYP YQQTNYTLEE S--------- -----KEWFE
TthGSTM11    ---------PI RYLLELAQYP YQQTNYTFEG A--------- -----NDWFE
TthGSTM12    ---------PI RYIFELAKFP YQQTLYTSAT A--------- -----SNWFG
TthGSTM13    ---------PA RYVLELAGIP YEDRLYTMQN R--------- -----ADWFE
TthGSTM14    ---------PI RYILELAQYP YSEKKYSQKE A--------- -----QEWFG
TthGSTM15    ---------PI RYLLELGHFP YTEKRYAQKD A--------- -----QEWFG
TthGSTM16    ---------PI RYLLELGHYP YVEKKYTQQD A--------- -----SEWFG
TthGSTM17    ---------PI RYILEIGKYP YKENQYKTP- ---------- -----AEWFE
TthGSTM18    ---------PI RYLLELGKQA YQDKKYSNKD ---------- ------EWFQ
TthGSTM19    ---------PS RYLLEISGVK YEEVRYTNP- ---------- -----ADWFG
TthGSTM20    ---------PA RYLLEISGVK YQDVRYSKP- ---------- -----ADWFG
TthGSTM21    ---------PS RYLLELSGVK YEEVRYSYPA ---------- -----AEWFG
TthGSTM22    ---------PS RYLLEISGVE YQETLYTDP- ---------- -----AQWFG
TthGSTM23    ---------PI KLLLELAGAQ YEVVNYSKP- ---------- -----DEWYA
TthGSTM24    ---------PA RYLLELTKTP YNNVQYVED- ---------- -----DKWFK
TthGSTM25    ---------AI RFLLEILKVE YTEKRYTFKN S--------- -----REWFE
TthGSTM26    ---------PV IFMLEILNIP YQVNIFDQN- ---------- ------TWFG
TthGSTM27    ---------PI RLLLEYLQVD YKEKLYNQDG ---------- -----EEWLN
TthGSTM28    ---------PI RLLLEYLQVE YKDKLYHENG ---------- -----EEWFN
TthGSTM29    ---------SI RLLLEYLQVE YQDKLYHENG ---------- -----EEWFG
TthGSTM30    ---------PI RLLLEYLEVG YQEKLYAEGG ---------- -----DEWYN
TthGSTM31    ---------II FPAIKYFTIA FEMFTYSHFQ NKN--NFFMV LEFVLTVCKL
TthGSTM32    ---------SI RFLLAYLGVE YNSKVYSTA- ---------- -----EEWFG
TthGSTM33    ---------SI RFLLAYLGVE YTNKAYSTP- ---------- -----EEWFG
TthGSTM34    ---------PI RFLLAYLGVQ YTNKAYANP- ---------- -----EEWFG
TthGSTM35    ---------PI RFLLAYLGVK YTNKTYASL- ---------- -----EEWFG
TthGSTM36    DQSVCNSCAI NYYMAQSSIA ASQNNNNQGV AAQCLVCPQG SGTLEDLNIQ
TthGSTM37    ---------PI RFLLAYLGVK YTNKTYASL- ---------- -----EEWFG
TthGSTM38    ---------PI RFLLAYLGVQ YTDKHYTKG- ---------- -----EDWFE
TthGSTM39    ---------PI RFLLAYLKIE HNSKIYTNF- ---------- -----DDWFA
TthGSTM40    --------PPV LSQINFQITK GETISFIGQI GSGKTSILYA IMKEIPRYKG
TthGSTM41    ---------SV RYLLAYLNVD YKHQAYYNP- ---------- -----QDWFA
TthGSTM42    ---------LS RYLLEYTGLK YKEKRYQKL- ---------- -----EEWFQ
TthGSTM43    ---------TI RYLLEYLEVP YEQKSYMTY- ---------- -----EEWFG
TthGSTM44    ---------QI RFLLEYVEAD YEEKTYIFSE PEQ------- -----DEWFK
TthGSTM45    ---------QI RFLLEYVEAS YEEKIYHFNN P--------- -----DEWFQ
TthGSTM46    ---------PL KFLLEYMGIP FEQKYYSYDN P--------- -----DEWYE
TthGSTM47    ---------PI RFLLEYTQTN YNEKIYYSEN E--------- -----SEWFQ
TthGSTM48    ---------PI RLLLNYLKLE YEDEIYPLSD R--------- -----EKWFN
TthGSTM49    ---------PI RMLLNYLQLP YTYKGYDLSS Y--------- -----NQWKQ
TthGSTM50    ---------PI RFLLAYLGVK YTNKTYASL- ---------- -----EEWFG
TthGSTM51    ---------AI RLLLEYLEVE YTEKQYYET- ---------- -----EQWFE
TthGSTM52    ---------TV RYILDLVDFP YFEHKYTSTS T--------- -----STEWE
TthGSTM53    ---------GL GVTGIFSGLY WYHGAFSHVE F--------- ----------
TthGSTM54    ---------GA GITGIVSGIY WYHGAFSDIE F--------- ----------
```

```
                ....|....| ....|....| ....|....| ....|....| ....|....|
                        560        570        580        590        600
TthGSTM1        KKKFELGLDF PNL-PYLIYG ---------- DFSISESQNI VNYLIELTN-
TthGSTM2        KKKSELGLDF PNL-PYLIHG ---------- DFSISESQNI VNYLIELTN-
TthGSTM3        KDKQELELDF PNL-PYLIHG ---------- DFSITESSNI ANYLIQLTN-
TthGSTM4        KDKQELELDF PNL-PYLIHG ---------- DFSITESSNI ANYLIQLTN-
TthGSTM5        KDKQELELDF PNL-PYLIHG ---------- DFSITESSNI ANYLIQLTK-
TthGSTM6        KDKLELGLDF PNL-PYLIHG ---------- DFSITESQNI VNYLIQLTN-
TthGSTM7        KDKKDLGLDF PNL-PYLIHG ---------- DFKITESQNI INYALDVTK-
TthGSTM8        KDKKELGLDF PNL-PYLIHG ---------- DFKITESQNI ISYALDITK-
TthGSTM9        KDKKELGLDF PNL-PYLIHG ---------- DFKITETQNI INYALDITK-
TthGSTM10       KDKKELGLDF PNL-PYLIHG ---------- DFKITESQNI VNYALDATK-
TthGSTM11       KDKKELGLDF PNL-PYLIHG ---------- DFKITESQNI VDYVLDLTK-
TthGSTM12       KDKQELGLDF PNL-PYLIKG ---------- DFKITESQNI VNYAIDITK-
TthGSTM13       KDKQTLGFDY PNL-PYIIHG ---------- DFKITESQNV VNYVIEVTN-
TthGSTM14       NDKQNLGLEF PNL-PYIFHG ---------- DYHLTEASNI ANYVLEITC-
TthGSTM15       KDKQNLGLEF PNL-PYITHG ---------- DYHLTEAANI ANYVIEITC-
TthGSTM16       KDKQKLGLEY PNL-PYLIQG ---------- DFHITEASNI ANYVIEITH-
TthGSTM17       KDSMSLGLQF PNL-PYIIKG ---------- DLKITESHNV AQYAIEVSN-
TthGSTM18       QDKLNLGLDF PNL-PYIIHG ---------- DVKMTESQNI VAYIIDLTK-
TthGSTM19       KDKYALGLSF PNL-PYLLDG ---------- DVKITESETI FDYLIHRLN-
TthGSTM20       KDKYALGLPF PNL-PYLIDG ---------- DVKITESETI FDYLIHKLN-
TthGSTM21       RDKYALGLPF PNL-PYLLDG ---------- EVKITESETI FDYLIQRLN-
TthGSTM22       KDKYSLDLPF PNL-PYLIDG ---------- DVKLTESETI FDYLVNKLN-
TthGSTM23       KDKLILGLPF PNL-PYLIEG ---------- DIKLTETETI FDYLVYKLK-
TthGSTM24       QDKLNLGLDF PNL-PYVIDG ---------- EFKLTEFVAI FDYLSEKYG-
TthGSTM25       EDKLKIGLDF PNL-PYFIDG ---------- NIKLSESNSI VTYILDKYS-
TthGSTM26       KEKEDLNLDF PNL-PFLIDN S--------N GMKITEIHNI VNYILYKYE-
TthGSTM27       VDKQQLKTNF PNL-PYIIDG ---------- DIVVTESKVI PIYLAKKFK-
TthGSTM28       TDKQELKTNF PNL-PYLIDG ---------- DVVVTESIVI PIYLAKKFK-
TthGSTM29       TDKKNLNTNF PNL-PYVIDG ---------- DVVVTESKVI PIYIIKKFK-
TthGSTM30       KDKRELKSNF PNL-PYLLDG ---------- DNVITESKVI PIYLIKKFK-
TthGSTM31       CIQLYMRIKY MLLFPFIVDL IENLIQLYNT IVKFFSSVKL LRLLNKLPDV
TthGSTM32       KDKNNLGLEF PNI-PYIIDG ---------- EFKLTESSAI PIYLLRKYK-
TthGSTM33       KDKNNLGLEF PNL-PYIIDG ---------- EFKLTESQAI PIYLLKKYK-
TthGSTM34       KDKNELGFDF PNI-PYLIDG ---------- DLKLTESSAI PIYLIRKHK-
TthGSTM35       KDKDNLGLEF PNL-PYLIDG ---------- DVKLTESFAI PVYLIKKYK-
TthGSTM36       GDISQCSICL ENY--FMVSP AIQS-----A QGIQPSAAQC ISCPNNSYNP
TthGSTM37       NDKDNLGLEF PNL-PYLIDG ---------- DVKLIESFAI PVYLIKKYK-
TthGSTM38       NDKKNLGMDF PNI-PYFID- ---------N DIKISESSAI PFYIIKKYK-
TthGSTM39       IDKQNLGLDL PNI-PFWID- ---------K DVKLTESAAI PVYLIKKCN-
TthGSTM40       QFFSTSNLAY VEQEPYILQG TV----RDNI LFGKTYDEDF YQQVVSACCL
TthGSTM41       KDKAQLKIEF SNL-PYLIDG ---------- EQKITDSYAI SIYIIRKYH-
TthGSTM42       KDKQGLGIEF ANL-PYLIDG ---------- DLKLTESHAV NLYIIRKSG-
TthGSTM43       KDKKELGADF PNL-PYIKQG ---------- DFILTESYAI IIYLCKKYN-
TthGSTM44       KDKKALKP-F PNL-PYIIDG ---------- DFYLSEHDVV IKYIVKKHPK
TthGSTM45       KDKKTLKP-F PNL-PYIIDG ---------- DFYLSEHDSV IKYIVKKHPK
TthGSTM46       KDKKTIKP-F SNL-PYLQTK ---------- DGILTETCSI IKYLLKRFPQ
TthGSTM47       KDQKQFKQ-F ANL-PYIIDG ---------- DLKLTDVQTI MRYIAKRNSN
TthGSTM48       FKR-NSQELF INL-PYVQIE SK----ETQS QSIYVESDSI SIFICQNFG-
TthGSTM49       VDKPALQSDF PNL-PYLKDG ---------- DYVLTESDAI AQYVCVKAN-
TthGSTM50       KDKDNLGLEF PNL-PYLIDG ---------- DVKLTESFAI PVYLIKKYK-
TthGSTM51       KDKKELNTNF PNL-PYLIDG ---------- EVVVTESIVI PIYLIKKLK-
TthGSTM52       K--------- ---------- ---------- -------QNV S---LDQTS-
TthGSTM53       TDSKYGPYDF VYF-NRIGSY ---------- ETLDSDWIKI KDEVENQFN-
TthGSTM54       RDSKYGPYDF VYY-KRVGSY ---------- QTLGSEWSKM SNEVQKQFS-
```

G-site

FIGURE S8

Partial alignment of the Mu class TthGSTs. TthGSTM names shaded in light blue are those that we consider not to be true GSTs. Shaded in yellow: identical amino acid residues. Inside red boxes: conserved motifs in the GST-NTER domains. Shaded in green: cis-Proline-loop (see text). Gray shading tyrosine residues (Y).
