# Supplementary material for: Tetrahymena thermophila glutathione-S-transferase superfamily: an eco-paralogs gene network differentially responding to various environmental abiotic stressors and an update on this gene family in ciliates
Source: Front Genet. 2025 Mar 7;16:1538168. doi: 10.3389/fgene.2025.1538168 (PMC11925944; doi:10.3389/fgene.2025.1538168)
Supplement: Supplementary file 12 [file DataSheet10.pdf]

```
                ....|....| ....|....| ....|....| ....|....| ....|....|
                        10         20         30         40         50
TthGSTT1        MTTK----PS TKLYIEWLSQ PSRAIVTFCL IENIPHELVE VRIKKLENRT
TthGSTT2        MQDQ---QQT LSLYMDWMSQ PSRCVAIFCM INKIPVDISE VKILKGQLRS
TthGSTT3        MSIKNLSQLN YEIFMDWGSQ PSRAVMTVVY FLKIPHKINE VRILKKQNVS
TthGSTT4        ---------- MKIFIDWISQ PSRAVVTYCL IENIPHEIIQ VRVNALEHRK
TthGSTT5        -------MNK LKLFYNSFSQ PSRAVKCLLK IGKVDYEEKF VNLAKGDQFK
                                   G-site

                ....|....| ....|....| ....|....| ....|....| ....|....|
                        60         70         80         90        100
TthGSTT1        PEYKKMFPTA KLPGMSETLE NGEQFNLFES HAIMRYLAD- --RYNKSNLY
TthGSTT2        QEYKRINPNM RVPTIKD--- --GKFVLYES HAILKYLIAS RAQYIPEHWY
TthGSTT3        EQYKKINPDQ KIPCIID--- -NENFFLNES HSIMRYFCQ- --LYGDNQLY
TthGSTT4        PEYIQINPSA KVPAISDRLE NGEIFNLFES HAIMRYLAD- --RYNKFKLY
TthGSTT5        PEVKSLNWNC QVPFIED--- --NGFVVFES HTIMRYIHQ- --RFNLDNSL
```

FIGURE S10

Partial alignment of Theta class TthGSTs. Shaded in yellow: identical amino acid residues. Inside red box: conserved motif in the GST-NTER domains (putative G-site). Shaded in green: cis-Proline-loop (see text). Shaded in light blue serine residues (S). Gray shading tyrosine residues (Y).
